# A High-Resolution Genetic Map of Yellow Monkeyflower Identifies Chemical Defense QTLs and Recombination Rate Variation

**DOI:** 10.1534/g3.113.010124

**Published:** 2014-03-13

**Authors:** Liza M. Holeski, Patrick Monnahan, Boryana Koseva, Nick McCool, Richard L. Lindroth, John K. Kelly

**Affiliations:** * Department of Entomology, University of Wisconsin, Madison, Wisconsin; † Department of Ecology and Evolutionary Biology, University of Kansas, Lawrence, Kansas

**Keywords:** inversions, mimulus, MSG, QTL, recombination

## Abstract

Genotyping-by-sequencing methods have vastly improved the resolution and accuracy of genetic linkage maps by increasing both the number of marker loci as well as the number of individuals genotyped at these loci. Using restriction-associated DNA sequencing, we construct a dense linkage map for a panel of recombinant inbred lines derived from a cross between divergent ecotypes of *Mimulus guttatus*. We used this map to estimate recombination rate across the genome and to identify quantitative trait loci for the production of several secondary compounds (PPGs) of the phenylpropanoid pathway implicated in defense against herbivores. Levels of different PPGs are correlated across recombinant inbred lines suggesting joint regulation of the phenylpropanoid pathway. However, the three quantitative trait loci identified in this study each act on a distinct PPG. Finally, we map three putative genomic inversions differentiating the two parental populations, including a previously characterized inversion that contributes to life-history differences between the annual/perennial ecotypes.

Advances in sequencing technology provide opportunities to simultaneously address multiple questions in evolutionary genomics. One goal is to discover genes that contribute to natural variation among populations or species, typically via quantitative trait loci (QTL) mapping. A second major goal is to characterize broad patterns and features of genomes, including linkage, recombination rate variation, and major chromosomal polymorphisms such as inversions. Both of these objectives rely on the construction of linkage maps, which in turn rely on genotyping many individuals at many marker loci. High-throughput genotyping based on next-generation sequencing techniques has dramatically increased our genotyping capacity, improving both accuracy and precision in estimating linkage maps (Elshire *et al.* 2011; Poland and Rife 2012; Slotte *et al.* 2012).

For QTL mapping, these techniques should lead to higher power to detect loci and a finer resolution of genomic locations of QTL. High-density maps also allow estimation of variation in recombination rate (cM/Mb) if the markers can be tied to a reference genome or a set of genomic contigs. This variation can be further dissected by the identification of chromosomal inversions, which exhibit highly reduced recombination, as well recombination hotspots, which show the opposite. In this paper, we use multiplexed shotgun genotyping (MSG, Andolfatto *et al.* 2011) on a large panel of recombinant inbred lines (RILs) from *Mimulus guttatus*. We map several QTL affecting secondary metabolite levels and also document both major and minor variation in recombination rate.

The dominant bioactive secondary metabolites of *Mimulus* consist of a suite of phenylpropanoid glycosides (PPGs) implicated in plant defense against herbivores (Holeski *et al.* 2013). PPGs are synthesized via the shikimic acid pathway, which is the source of a wide array of secondary compounds across higher plant species (Knaggs 2003; Fraser and Chapple 2011). PPGs typically consist of caffeoyl or hydroxytyrosol moieties bonded to a central β-glucopyranose sugar (Mølgaard and Ravn 1988). PPGs act as generalist herbivore feeding deterrents and as specialist herbivore feeding stimulants, and the production of these compounds is genetically variable within and among natural populations (Mølgaard 1986, Holeski *et al.* 2013). The RILs of this study are genetic mosaics of two parental genomes: Point Reyes (PR) is a low-elevation, perennial population located in California, whereas Iron Mountain (IM) is an alpine, annual population in Oregon (Holeski 2007). We previously mapped QTL for the structural defensive trait of trichome density in this RIL panel (Holeski *et al.* 2010). The identification of QTL for PPG production reported in this paper provides a first step toward understanding the genetic architecture of PPGs in *M. guttatus* as well as a list of candidate genes for further localization of the responsible polymorphisms.

We also use the high-density linkage map to estimate recombination rate and variation therein. Recombination rates vary greatly among organisms, which has important implications for many aspects of evolution. Recombination rate is expected to evolve in responses to changes in mating system (Roze and Lenormand 2005), selection patterns (Lenormand and Otto 2000), and epistasis (Feldman *et al.* 1980; Kondrashov 1982, 1988; Barton 1995). Starting with several thousand genomic scaffolds (the v1 genome build of *M. guttatus*; Hellsten *et al.* 2013), we use the RIL genotypes to assemble scaffolds into 14 linkage groups. With map position estimates for markers, we then estimate recombination rate both within scaffolds, where we have an estimate of the physical distance between marker, and between scaffolds, where we do not.

## Materials and Methods

### Derivation of the mapping population

We developed RILs from a cross between plants derived from two natural populations, IM and PR. IM is an annual population in the Cascade Mountains of central Oregon, whereas PR is a low-elevation, perennial population in the fog belt of coastal northern California. An IM plant from the inbred line IM 767 (father) was crossed to a PR plant (mother). A single F_1_ individual from this cross was self-fertilized to form 1000 F_2_ individuals, each of which founded a distinct recombinant lineage. These lines were continued through single seed descent for six subsequent generations, resulting in approximately 500 RILs (after line loss) in the F_8_ generation, used in the work described herein.

### Genotyping by sequencing

We harvested leaf and bud tissue from adult plants from each of 481 RILs into 96-well plates with two stainless steel balls in each well. For each plate, we froze tissue with liquid nitrogen and homogenized using a modified reciprocating saw (Alexander 2007). We added cell lysis buffer (0.1 M Tris, 55.9 mM CTAB, 20 mM EDTA, 0.5 mM PVP, 1.4M NaCl, 5 mM ascorbic acid) at 60° for 20 min followed by the addition of phenol:chloroform:isoamyl alcohol (25:24:1) and gentle mixing for 20 min on a nutating platform. After extracting the aqueous layer, we incubated the samples with RNAse (50 µL at 10 mg/mL) for 20 min at 37°. We performed a second extraction by adding chloroform:isoamyl alcohol (24:1), mixing, and extracting the aqueous layer. We then added 100 µL of 2M NaCL with 4% PEG and incubated at 4° for 15 min. After centrifugation and extracting the aqueous layer, we precipitated the DNA with cold absolute isopropanol followed by two washes with 70% ethanol. We dried the DNA pellets, rehydrated with TE, and quantified the DNA using a Qubit fluorometer.

We generated genomic libraries for genotyping individuals using the multiplexed shotgun genotyping method described in Andolfatto *et al.* (2011). Briefly, we pooled 96 individuals into an Illumina library by using a set of 96 unique bar-coded-adapters (BCAs). Each BCA contains the Illumina sequencing primer followed by a unique 6-bp barcode that is used to delineate samples after sequencing. In a 96-well plate, we digested 50 ng of genomic DNA (3 U *Mse*I; NEB Biolabs) in a 20-µL reaction for 3 hr at 37° followed by heat inactivation at 65° for 20 min. To each well, we added 5 pmol of a unique BCA followed by ligation master mix containing 400 U of T4 DNA ligase (NEB Biolabs) for a total volume of 50 µL. We performed the ligation in a thermocycler at 16° for 3 hr followed by heat inactivation at 65° for 10 min. We precipitated DNA in each well by adding 5 µL of sodium acetate and 50 µL of isopropanol. We pooled the entire contents of the plate into a single tube, added 1 µL of glycogen, and refrigerated overnight at 4°. Following resuspension with TE, we removed linker-dimers using Agencourt AMPure beads at 1.5 bead-mixture:sample-volume ratio.

We size-selected our library for fragments between 250 and 300 bp using a 2% GTG agarose (NuSieve) gel with an adjacent 50-bp ladder as a guide. We performed gel extraction using the QIAquick Gel Extraction kit and eluted with 35 µL of EB buffer. To obtain a sequencable quantity of our size-selected library, we performed 8 polymerase chain reactions (PCRs) at 14 cycles using Phusion High-Fidelity PCR Master Mix and primers that bind to common regions in the BCAs. We pooled these reactions and did two rounds of PCR cleanup using Agencourt AMPure beads at a 0.8 bead-mixture:sample-volume ratio. We concentrated the sample by eluting with 200 µL of TE in the first round of cleaning and 35 µL of QIAGEN EB buffer after the second round.

We prepared eight 96-plex libraries, distributing DNA from many RILs into multiple different libraries. We also included 12 samples of DNA from the IM767 line. As IM767 is a highly inbred line, this DNA should be nearly identical to that of the sire for the RIL population. Sequencing was performed on an Illumina HiSequation 2000 for single-end, 100-bp reads. Five of the libraries were sequenced at the Duke Genomics facility and three at the University of Kansas medical center.

### Determining RIL genotypes from sequence data

After demultiplexing, we processed reads from the RILs and IM767, first with Scythe (https://github.com/vsbuffalo/scythe/) to remove adaptor contamination, and then with Sickle (https://github.com/najoshi/sickle/) to trim low-quality sequence. Using BWA with default parameter values (Li and Durbin 2009), we mapped the processed reads, one sample at a time, to the v1.1 draft of the *Mimulus guttatus* genome (http://www.phytozome.net/). This build consists of 2216 scaffolds ranging from a maximum size of 4.9 Mb (approximately 15% of an average *M. guttatus* chromosome) down to 1 kb. Highly repetitive regions of the reference sequences were masked prior to mapping. Following read mapping, we identified putative SNPs using the UnifiedGenotyper algorithm in the Genome Analysis ToolKit (GATK; McKenna *et al.* 2010).

We filtered SNPs based on several criteria using custom python scripts. We considered only those SNPs where (1) two bases segregated, (2) the IM767 samples yielded an unambiguous base call, (3) the total read depth across all RILs was between 50 and 1000, (4) the frequency of alternative bases across RILs was between 0.2 and 0.8, and (5) the fraction of RILs called as heterozygous was less than 0.25. The genotype of a RIL at a SNP was called as IM/IM if the sample frequency of the IM base was greater than 0.9, PR/PR if that frequency was less than 0.1, and IM/PR (heterozygous) otherwise. Criterion (3) was chosen to bracket the median depth of 338 across RILs for all SNPs called by GATK. The application of these filters reduced the number of SNPs from 1.27 million to 264,226. We then filtered SNPs based on consistency of genotype calls among neighboring markers within RILs. If the genotype call for a SNP differed from the calls at other SNPs within 50 kb on the scaffold more than half the time, it was excluded as spurious.

*Mse*I cuts very frequently in the *Mimulus* genome. Interrogating the reference genome, we estimate that approximately 40–45% of genome is 100- to 500-bp intervals between two *Mse*I recognition sequences (such fragments could be sampled into our libraries). The consequence is that we have a very large number of informative SNPs, with relatively low coverage per SNP. For a typical SNP, a minority of RILs had a substantial number of reads. For this reason, we implemented a window-based method to make genotype calls on RILs, aggregating information across SNPs within 50 kb windows within each RIL. A 5 Mb scaffold of the reference genome was thus characterized by 100 contiguous markers. Within each window of each RIL, we counted the number of SNPs with each genotype. As expected, the typical window contained SNPs called nearly all as IM/IM or all as PR/PR, with adjacent windows exhibiting the same predominant genotype. However, the data also clearly indicate regions of residual heterozygosity within many RILs—strings of 10 or more windows with substantial representation of alleles from each parent. Denoting p the frequency of the IM base across called SNPs within a window, we called the marker IM/IM if p > 0.9, PR/PR if p < 0.1, IM/PR if 0.4 < p < 0.6, and missing data otherwise.

**Figure 1:**
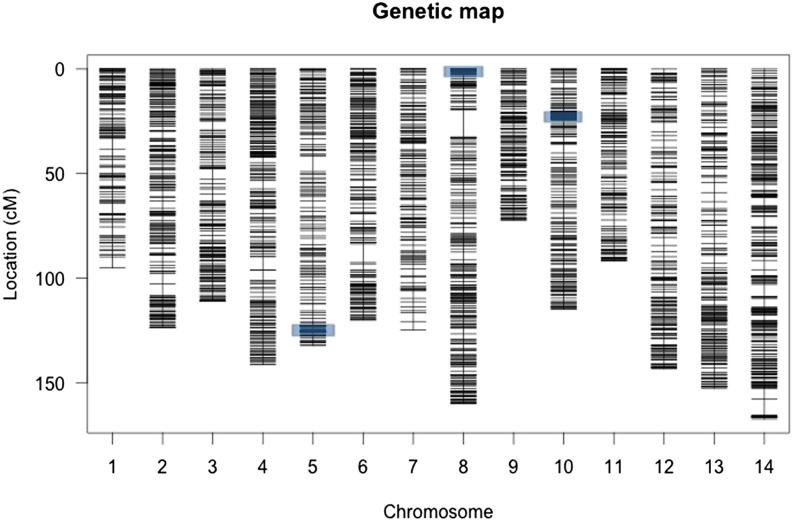
The genetic map with each marker identified with a horizontal line. The location of hypothesized inversions, where many markers have the same map positions are indicated by blue shading.

We built the linkage map using the 100 RILs with the most complete genotyping data and restricted attention to those markers that were called in at least 75% of these RILs. We formed linkage groups using AntMap version 1.2 (Iwata and Ninomiya 2006) with a threshold of 0.15 in the “nearest-neighboring locus” grouping method and with recombination as the grouping criterion. These settings yielded 14 linkage groups, each with a large number of markers. To order markers within linkage groups, we used the ‘Order Markers’ option of AntMap with default settings (Haldane mapping function), except allowing a greater number of runs (20 instead of 3) to confirm an optimal solution. In the great majority of cases, we found that markers predicted to be contiguous because they were located on the same scaffold of the v1 genome build mapped together in the linkage map. However, we did ‘break’ some scaffolds when different parts of these scaffold mapped to different linkage groups. We preserved the marker ordering predicted by the v1 scaffolds at small genomic scales by manually reordering the map. We then input the marker-ordered genotype set into R/qtl (v. 1.26-14; Broman *et al.* 2003) to estimate the map length of each linkage group, specifying cross type as ‘RILs produced via selfing.’ Finally, we matched our 14 linkage groups to those identified in previous *M. guttatus* mapping studies (Fishman *et al.* 2002; Hall and Willis 2006) by locating the MgSTS markers of these previous studies to the v1 genome build. As a consequence, our Chromosome 1 (Figure 1) is the same as LG1 of Fishman *et al.* (2002), and so on.

### Recombination rate

To estimate recombination rate per base pair, we estimated the map length of each unbroken scaffold of the genome build from the R/qtl output. Assuming that the physical length of these genomic intervals is correctly predicted by the genome sequence, the map length divided by the segment size yields a recombination rate (cM/Mb) point estimate for each scaffold. Additionally, the total genetic and physical map length accounted for by this within-scaffold analysis was used to determine the global rate of recombination within mapped portions of the genome. Using the genetic distance estimated from the assembled linkage map described previously, we inferred the genome-wide recombination rate as well as the rate pertaining to genomic regions between scaffolds. We obtained confidence intervals on the chromosome-specific recombination rates by block bootstrapping entire scaffolds (block sizes simulated from a geometric distribution with a mean of 3; 10,000 bootstrap replicates). This procedure was used to accommodate potential non-independence of recombination rate estimates among adjacent scaffolds.

### Plant materials and phytochemistry measurements

Seed from 211 RILs was grown in three rounds (“grow-ups”) in the greenhouse at the University of Wisconsin, Madison. We used Fafard 3B potting soil. Plants received supplemented lighting (16-hr days with high pressure sodium lamps), were bottom-watered daily and fertilized weekly (with Blossom Booster, J.R. Peters, Allentown, Pennsylvania). Twelve days after seeding, we transplanted each seedling (three replicates per RIL per grow-up) into a two-inch pot. We randomized pot positions within flats and rotated flats daily on the greenhouse bench to minimize environmental effects. When the leaves of the fourth leaf pair of each plant were fully expanded, we harvested the third leaf pair of each plant. We flash-froze leaf tissue using liquid nitrogen, then freeze-dried and finely ground the tissue in a small-capacity ball mill (dental amalgamator with steel bearings). Ground samples were stored at −20**°**C until chemically analyzed.

We extracted PPGs from the dried, ground samples with methanol (10 min sonication, 12 hr at 21° in the dark). We vacuum dried 100 µL of the extract and redissolved it in 100 µL of a water/catechol mixture for analysis. We quantified the PPG content of each sample via high-performance liquid chromatography [HPLC; Hewlett Packard 1090 HPLC with a diode array detector and Vydac C18 analytical column (4.6 × 250 mm, 5 µm particle size; W.R. Grace & Co., Columbia, MD) maintained at 30°] or via ultra-high pressure liquid chromatography [UHPLC; Waters Acquity I-Class UHPLC with an Acquity photodiode array detector and a Waters C18 CSH analytical column (2.1 × 100 mm, 1.7-µm particle size; Waters Corporation, Milford, MA) maintained at 30°]. HPLC-run conditions are described in Holeski *et al.* (2013). UHPLC-run conditions included a binary mobile phase gradient with water (0.1% formic acid) as mobile A and acetonitrile (0.1% formic acid) as mobile B at a constant total flow rate of 0.5 mL/min. The gradient for each run consisted of B initially set at 1%, 20% at 4 min, 40% at 6 min, and 95% from 9 to 10 min. From 10 to 11 min, mobile B returns to 1% to re-equilibrate the column. We injected 2 µL of the standards and samples, monitored ultraviolet signals at 274 (catechol) and 340 nm (PPGs), and used a diode array detector to collect ultraviolet data from 190 to 400 nm.

For all samples (regardless of run method), we calculated PPG quantities as verbascoside equivalents, using a standard solution of pure verbascoside (isolated from *Plantago lanceolata* by M.D. Bowers, University of Colorado, Boulder, CO) prepared in a similar fashion as the samples with the internal standard solution. We compared peak areas of the verbascoside standard to all sample PPGs, all of which were normalized by the catechol internal standard peak area from each chromatogram. All sample PPG peaks were within a linear range established for the verbascoside standard and PPG concentrations were calculated as mg/g dry weight.

### RIL variation and QTL mapping

The distributions of concentrations for each PPG were right-skewed (Figure 3). For this reason, we log-transformed [log (PPG concentration +1)], each PPG prior to statistical analysis. We assessed differences in PPG levels among RILs, among grow-ups, and between quantification methods using General Linear Models (GLM ANOVAs, Minitab 14; Minitab Inc., State College, PA). RIL (random) was nested within grow-up. Grow-up and quantification method were fixed factors. The variance components from each analysis of variance were used to calculate the variance within and among RILs for each PPG. RIL means for each PPG were calculated as residual values after correcting for any effects of grow-up and quantification method.

For each trait, we performed standard interval mapping (“scanone” function with default settings) using the multiple-imputations method with 32 imputations. The multiple-imputations method alleviates the issue of missing genotype data by imputing all missing genotypes numerous times conditional on observed genotype data at neighboring markers and storing these imputations. The final result of standard interval mapping is a combination of the results from mapping each imputation separately. We used these results to establish an initial QTL model consisting of the markers that exceeded the 5% LOD (logarithm of the odds) threshold. These LOD/significance thresholds were determined using 1000 permutations (Churchill and Doerge 1994). Increasing the permutation number to 10,000 had very minimal effects on LOD thresholds.

After establishing the initial QTL model for each trait, we searched for additional QTL while controlling for the QTL in the model (“addqtl” function). With the finalized QTL model, we extracted effect sizes, explained phenotypic variance, LOD scores, and 1.8-LOD intervals for each QTL. Effect sizes are reported in terms of phenotypic SDs, which were found by taking the square root of the estimated within-RIL variance in the analysis of variance table. After delineating QTL, we used Blast2Go (Conesa and *et al.* 2005; Conesa and Götz 2008; Götz *et al.* 2008, 2011) and Phytozome (v. 9.0; Goodstein *et al.* 2012) to determine the functional basis of genes in other plant species that share high sequence similarity, and are likely homologous, to *M. guttatus* genes underlying each region.

## Results

### Genetic map

**Figure 2:**
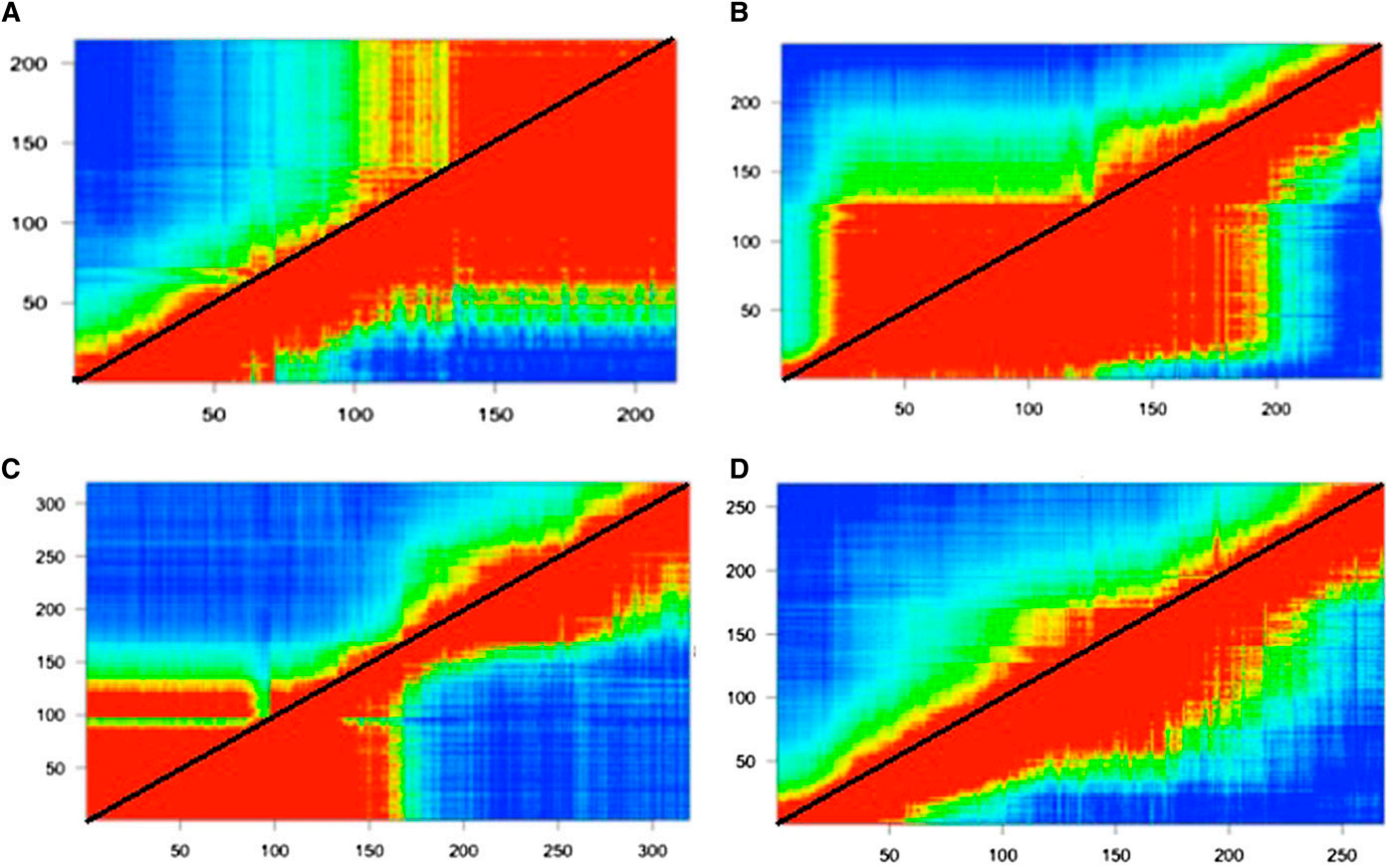
Heat map of the estimated recombination fraction between markers (below diagonal) and the corresponding LOD scores (above diagonal). The LOD score is for a test of the null hypothesis that the true recombination fraction between markers is 0.5 (free recombination). Low estimates of recombination fraction and corresponding high LOD scores are shaded red while blue represents the converse. (A) Chromosome 5, (B) Chromosome 10, (C) Chromosome 8, and (D) Chromosome 4. Putative inversions are apparent in (A), (B), and (C). (D) illustrates a chromosome lacking recombination suppression.

Our genetic map based on 3073 markers has a total length of 1750 cM with an average spacing of 0.6 cM (Figure 1). Supporting information, Table S1 provides the full genetic map (rows 1−3) and the genotype calls for 480 RILs (rows 4−483). Chromosomes range from 72 cM (chromosome 9) to 167 cM (chromosome 14). There is little variation in the average spacing per chromosome (range 0.4–0.8), but significant variation in the maximum spacing per chromosome. The maximum intermarker distance (13.2) is on chromosome 8, flanking a previously discovered inversion (Lowry and Willis 2010; Lowry *et al.* 2012). There are three sections of the map where a large number of physically dispersed markers map to the same location. This is illustrated by the estimated recombination fractions between all markers on a chromosome (Figure 2). Chromosome 4 exhibits the typical pattern (Figure 2D): Recombination fraction increases continuously with the physical distance between markers. The ‘block structure’ evident on chromosomes 5, 8, and 10 is caused by many consecutive markers with little to no recombination (Figure 2, A–C).

**Figure 3:**
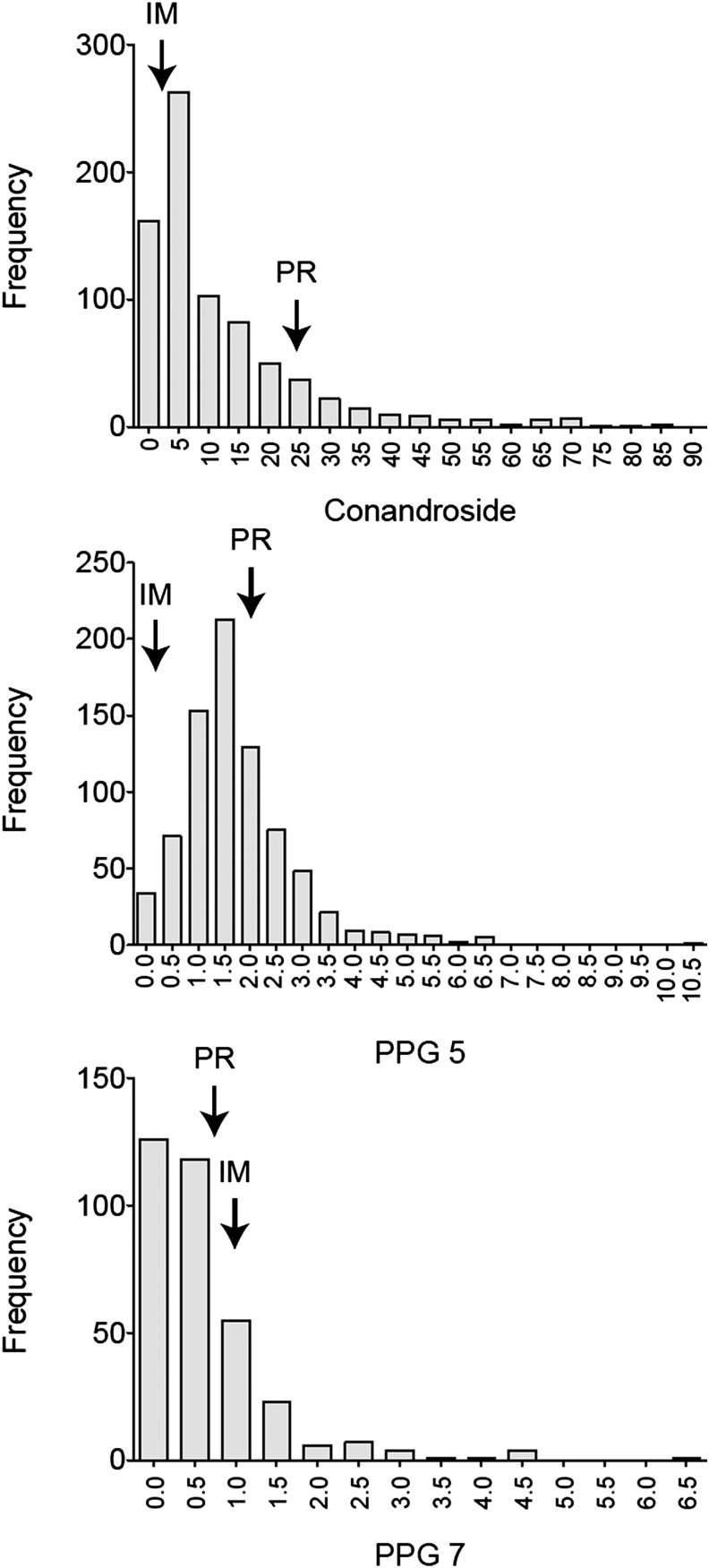
The frequency distributions of untransformed recombinant inbred line (RIL) values for Conandroside, PPG 5, and PPG 7. Approximate values for parent plants [IM 767 (IM) and Point Reyes (PR)] are indicated with arrows in each histogram.

### Recombination rate

Approximately 1200 cM of the map is within 277 unbroken scaffolds of the v1.1 genome build, which represents ~185 Mb of the genome. The remaining 550 cM is between scaffolds which represents about 265 Mb (assuming a total haploid genome size of 450 Mb, http://www.mimulusevolution.org/). Thus, the average recombination rate within the mapped portion of the genome, 6.6 cM/Mb, is substantially greater than the average rate in the unmapped portion, 2.1 cM/Mb. The genome-wide estimate is (1750 cM/450 Mb) ~3.9 cM/Mb. Chromosome mean recombination rate estimates (and confidence intervals) within scaffolds are reported in Table 1. Confidence intervals for all chromosomes contain the estimate of 6.6 cM/Mb, indicating no significant differences in intra-scaffold recombination rate across chromosomes.

**Table 1:** A chromosome by chromosome summary of intrascaffold recombination rate estimates

Chromosome	cM/Mb	Confidence Interval (95th Percentile)
1	7.2	(4.7−8.9)
2	6.2	(5.0−7.4)
3	6.6	(5.1−8.1)
4	6.8	(5.7−7.7)
5	7.6	(5.3−10.3)
6	5.8	(5.0−6.5)
7	8.1	(5.6−10.1)
8	7.1	(3.5−9.8)
9	5.9	(4.9−7.4)
10	6.7	(5.5−8.9)
11	5.2	(4.1−6.1)
12	7.6	(5.2−8.9)
13	6.9	(5.8−8.1)
14	5.7	(4.3−7.2)
Global	6.6	(6.1−7.1)

The recombination rate estimates from each scaffold exhibit appreciable variation (Figure S1), although estimates from smaller scaffolds, which yield the most extreme values, are subject to high estimation error. Focusing on scaffolds in the upper 50th percentile of the physical size distribution, we see recombination rates ranging from near zero up to ~15 cM/Mb. In line with expectations, the scaffolds that map to putative inversions exhibit minimal recombination. Considering scaffolds with physical length >500kb, we find no correlation between recombination rate and gene density (Figure S2). Finally, Figure S3 illustrates patterns of segregation distortion and residual heterozygosity across chromosomes in the RIL population (the detailed information about line specific genotypes is in Table S1).

### QTL mapping

Individual PPG concentrations differed substantially across RILs, for each of the three PPGs. The full summary of tests is reported in Table S2. RIL mean PPG concentrations were positively correlated, with Pearson correlation coefficients of 0.390, 0.419, and 0.361 for correlations between conandroside and PPG 5, conandroside and PPG 7, and PPG 5 and PPG 7, respectively (all associated p-values were less than 0.001).

**Figure 4:**
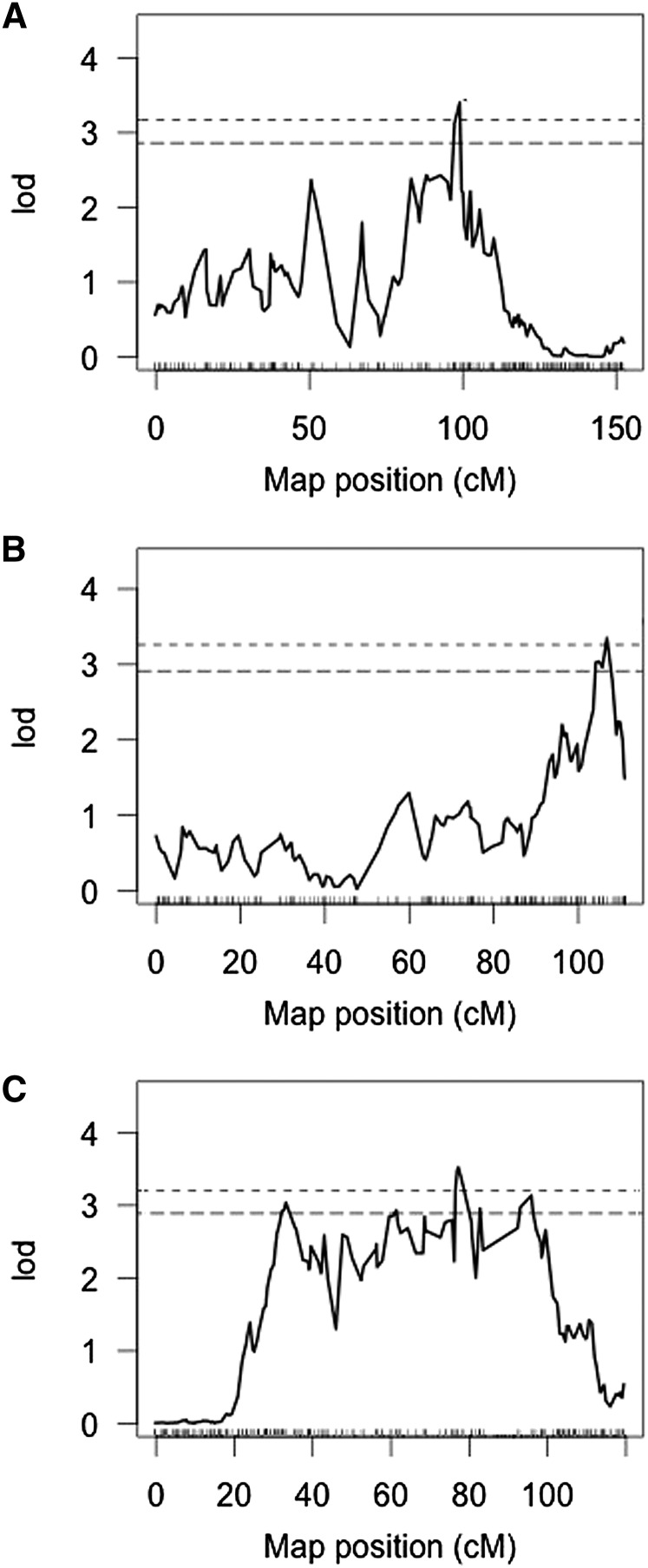
LOD profiles for each trait. Only showing the chromosomes containing a significant QTL. Upper dashed line represents 0.05 significance threshold, and lower dashed line represents 0.10 significance. (A) QTL for PPG7 on Chromosome 13. (B) QTL for Conandroside on Chromosome 3. (C) QTL for PPG5 on Chromosome 6.

QTL mapping results are summarized in Table 2. We discovered three significant (genome wide 5% level) QTL for PPGs. Conandroside, PPG5, and PPG7 each had a single underlying QTL detected, with no overlap of QTL across traits. The LOD profiles in Figure 4 provide the locations of each QTL on linkage groups 3, 6, and 13. A search for additional QTL after controlling for the effects of the initially identified QTL showed no evidence for additional QTL. LOD intervals for chemistry traits ranged from 19 cM (~15% of the chromosome) to 74 cM (55% of the chromosome). Using the genome-wide recombination rate, this corresponds to roughly 5-Mb and 20-Mb intervals for the QTL, respectively. The QTL exhibited relatively small effects and explained a minor portion of variance when fitting a linear model with QTL as predictor and chemistry trait as response. Power to detect our observed QTL was generally low as a result of limited phenotype data (of the 481 RILs genotyped, only 169 were phenotyped), high phenotypic error variance, and non-normal distributions of the phytochemical traits (Beavis 1998). This also prevented fine-scale localization of QTL, despite high marker density.

**Table 2:** QTL mapping results.

Trait	Chr.	LOD-Interval, cM	% Chr.	LOD	P-Value	% Var
Conandroside	3	91.7−111.0	15.8	3.36	0.039	8.75
PPG7	13	45.5−105.1	34.4	3.40	0.035	13.16
PPG5	6	17.0−91.5	55.3	3.52	0.023	9.15

LOD-Interval is for a 1.8 LOD drop. % Chr. is the percentage of the chromosome contained in the LOD-interval. % var is the percentage of variance explained (using sums of squares) by fitting a linear model with QTL as predictor and chemistry traits as response. LOD, logarithm of the odds; QTL, quantitative trait loci.

We identify several putative defense response genes within the QTL regions. A gene identified within the QTL for conandroside on chromosome 3 shares 86% sequence similarity with the ACQ59091 gene in upland cotton (*Gossypium hirsutum*; Fan *et al.* 2008), and 94% sequence similarity with the AGU43757 locus in Chinese white poplar (*Populus tomentosa*; Gai *et al.* 2013). A candidate gene underlying the PPG 5 QTL on chromosome 6 shares 77% sequence similarity with XP_002325926 in black cottonwood (*Populus trichocarpa*; Tuskan *et al.* 2006). Finally, we identified a candidate gene underlying the PPG 7 QTL on chromosome 13 that shares 92% similarity to locus GRMZM2G033555 of *Zea mays* (Schnable *et al.* 2009).

## Discussion

### Genomic inversions

Large structural variants, such as inversions, are common between species (Stebbins 1958) and may play an important role in adaptive divergence of populations (King 1993; Dobzhansky 1970). A recent study in *M. guttatus* has shown that an inversion on chromosome 8 containing several hundred genes contributes to the differential adaptation of annual and perennial ecotypes (Lowry and Willis 2010). Loci affecting multiple life-history traits map to the inversion. Generally, annual plants flower much sooner than perennials, which devote almost the entire first season to vegetative growth. Here, we have identified this same inversion in the IMPR RIL panel: 54 distinct markers spanning 2.95 Mbp of genome sequence map to essentially the same position at the upper end of chromosome 8 (Figure 1 and Table S1). However, we find two other genomic regions that yield the same pattern (Figure 2). Sixty markers spanning at least 3.75 Mbp at the lower end of chromosome 5 map together. The region on chromosome 10 consists of 75 markers spanning at least 4.1 Mbp.

These inversions must be classified as putative until we have direct cytological evidence. However, it is still worth considering their genomic locations in relation to previous QTL mapping studies. We expected the chromosome 8 inversion to be segregating in this cross given that the parents were sampled from annual (IM) and perennial (PR) populations. Lowry and Willis (2010) showed that this inversion distinguishes numerous annual and perennial populations that are geographically proximal. It is not clear whether the inversions on chromosomes 5 and 10 are also segregating in previously published *M. guttatus* genetic maps (summarized in Fishman *et al.* 2014) because previous maps typically involved 100-200 markers which implies an average marker spacing of 2−5 Mbp. The inversions identified here are of this size (3-4 Mbp) and a much higher marker density (up to 20 markers per Mbp in the present study) is needed to reliably identify recombination suppression at this genomic scale.

At present, it is unclear whether important genetic differences between *Mimulus* populations map to the inversions on chromosomes 5 and 10. The only previous QTL study of the IMPR RIL panel (Holeski *et al.* 2010) did map trichome density QTL on chromosomes 5 and 10. However, neither locus is within the putative inversions. Recently, Friedman and Willis (2013) mapped a locus affecting vernalization, an important life history trait of *M. guttatus*, to a marker (MgSTS 122) that resides within the chromosome 5 inversion. It is not clear whether the inversion is segregating in this cross between two perennial populations. Fortunately, the use of genotyping-by-sequencing should provide much higher resolution in future mapping studies and we will consequently obtain a more complete picture of geographical variation in genome structure.

### Recombination rate variation

The RIL genotyping combined with the v1 genome build of *M. guttatus* yields two different recombination rates, a genome wide rate and a rate specific to assembled scaffolds. The genome-wide rate, 3.9 cM/Mb, is consistent with previous genetic maps from *M. guttatus* (Fishman *et al.* 2014). It is higher than estimates from animals, which tend to range from ~0.5 to 1.5 cM/Mb (Nachman 2002; Jensen-Seaman *et al.* 2004), but comparable to *Arabidopsis thaliana* (4.6 cM/Mb; Drouaod *et al.* 2006). The high rate of *A. thaliana* is predicted by population genetic theory demonstrating that higher recombination should evolve in self-fertilizing species (Wright *et al.* 2008). *M. guttatus* is a mixed-mating species with outcrossing frequency varying extensively among populations.

The average recombination rate within genomic scaffolds was about three times greater than the estimated rate within unmapped portions of the genome. This difference might reflect a higher recombination rate in euchromatic than in heterochromatic DNA (*e.g.*, Kim *et al.* 2005). About one-half of the *M. guttatus* genome consists of transposable elements (Hellsten *et al.* 2013), and we masked repetitive regions before mapping reads to scaffolds in order to reduce the rate of mismapping and consequent genotyping errors. Heterochromatin is often highly repetitive, and if it is overrepresented in the interscaffold portions of the genome, then a lower recombination rate for this component is predicted (Dooner and He 2008).

Genotyping of RILs, as done here, provides a coarse characterization of recombination rate variation, on the scale of hundreds of thousands of bp in *Mimulus*. A recent study of linkage disequilibrium (LD) in *M. guttatus* (Hellsten *et al.* 2013) provides much finer scale information. The advantage of inferring recombination from LD is that it integrates the many recombination events occurring in the history of a population, instead of the rather few that occur in the generation of a mapping population. The difficulty is that LD is also influenced by mutation, migration, and selection and thus estimates are contingent on a particular evolutionary model. These issues aside, Hellsten *et al.* (2013) found that recombination rate is highest in genic regions, particularly at the 5′ end of genes. Consistent with this, we find that recombination rate is higher in the mapped portion of the genome, which contains the bulk of expressed genes, than in the unmapped portion of the genome. However, within mapped scaffolds, we failed to find any association between gene density and local recombination rate (Figure S2).

### PPG QTL

Although we know a great deal about the organization and regulation of enzymatic pathways leading to secondary compounds in plants (*e.g.*, Grubb and Abel 2006; Halkier and Gershenzon 2006), we have only recently begun to characterize the pattern and effects of polymorphisms in nature (Kliebenstein *et al.* 2001). Our study is a first attempt to characterize the genetic architecture of multiple PPGs, the dominant group of bioactive secondary compounds in *M. guttatus*. Our results identify a few loci, each of modest effect, and each of which appear to affect a distinct branch in the PPG pathway.

The absence of QTL affecting multiple PPGs (*e.g.*, via action on a common precursor in the pathway) is surprising, given the strong, positive correlations between certain PPGs as well as previous studies reporting mixtures of common and unique QTL affecting secondary compounds such as terpenes (Henery *et al.* 2007; O’Reilly-Wapstra *et al.* 2011) and glucosinolates (Kliebenstein *et al.* 2001; Feng *et al.* 2012). It should be noted that this might simply be due to a lack of statistical power and is not a true reflection of the underlying genetic architecture. That said, our results suggest that individual PPGs may be genetically controlled independently of one another, thus increasing the complexity and lability of the secondary compound ‘portfolios’ within *M. guttatus*.

Within detected QTL, we identified multiple defense response genes, a broad class of genes encoding diverse enzymatic activities. These genes share the feature that expression is induced by a secondary organism or by wounding (Bowles 1990; Li *et al.* 1999) which is in line with the role of PPG’s as herbivore deterrents. The genes identified within the conandroside QTL on chromosome 3 influence production of cinnamyl alcohol dehydrogenase (Fan *et al.* 2008; Gai *et al.* 2013), an enzyme catalyst in the phenylpropanoid pathway that plays a major role in plant defense against biotic stressors. Increased levels of activity in cinnamyl alcohol dehydrogenase have been found in response to both herbivory and pathogen infection (Smit and Dubery 1997; Barakat *et al.* 2010). The locus identified within the PPG 5 QTL on chromosome 6 affects expression of chalcone isomerase (Tuskan *et al.* 2006), a key enzyme of the phenylpropanoid pathway with functions related to biotic defense and response to abiotic stressors (Dao *et al.* 2011). Finally, the locus within the PPG 7 QTL affects activity of cinnamoyl-CoA-reductase (Schnable *et al.* 2009), another enzyme in the phenylpropanoid pathway (Lauvergeat *et al.* 2001). When the biosynthetic pathway of PPGs becomes more fully characterized, candidate gene network filtering (*e.g.*, Chan *et al.* 2011) should further advance our understanding of herbivory defense in *M. guttatus*.

Advances in sequencing technology and methods enabled us to construct a very dense linkage map for a panel of RILs derived from an interpopulational cross of *Mimulus guttatus*. We demonstrate the utility of such a map in quantifying genome-wide recombination rates, identifying genomic inversions, and QTL for ecologically relevant traits. Future work will determine whether life-history and other ecologically relevant traits will map to the newly-identified inversions, as well as the extent to which individual PPGs have independent genetic control.

## 

## Supplementary Material

Supporting Information

## References

[bib1] Alexander P. J., 2007 Recovery of plant DNA using a reciprocating saw and silica- based columns. Mol. Ecol. Notes 7: 5–9

[bib2] Anderson J. T. Mitchell-Olds T., 2011 Ecological genetics and genomics of plant defences: evidence and approaches. Funct. Ecol. 25: 312–324 21532968 10.1111/j.1365-2435.2010.01785.x PMC3082142

[bib3] Andolfatto P. Davison D. Erezyilmaz D. Hu T. T. Mast J., 2011 Multiplexed shotgun genotyping for rapid and efficient genetic mapping. Genome Res. 21: 610–617 21233398 10.1101/gr.115402.110 PMC3065708

[bib4] Barakat A. Bagniewska-Zadworna A. Frost C. J. Carlson J. E., 2010 Phylogeny and expression profiling of *CAD* and *CAD-like* genes in hybrid *Populus* (*P. deltoides* × *P. nigra*): evidence from herbivore damage for subfunctionalization and functional divergence. BMC Plant Biol. 10: 100 20509918 10.1186/1471-2229-10-100 PMC2887455

[bib5] Barton N. H., 1995 A general model for the evolution of recombination. Genet. Res. 62: 123–144 7605514 10.1017/s0016672300033140

[bib6] Beavis W. D., 1998 QTL analyses: power, precision, and accuracy, pp. 145–162 in Molecular Dissection of Complex Traits, edited by Paterson A. H. CRC Press, Boca Raton, FL

[bib7] Bowles D. J., 1990 Defense-related proteins in higher plants. Annu. Rev. Biochem. 59: 873–907 2197993 10.1146/annurev.bi.59.070190.004301

[bib8] Broman K. W. Wu H. Sen Ś. Churchill G. A., 2003 R/qtl: QTL mapping in experimental crosses. Bioinformatics 19: 889–890 12724300 10.1093/bioinformatics/btg112

[bib9] Chan E. K. Rowe H. C. Corwin J. A. Joseph B. Kliebenstein D. J., 2011 Combining genome-wide association mapping and transcriptional networks to identify novel genes controlling glucosinolates in *Arabidopsis thaliana*. PLoS Biol. 9: e1001125 21857804 10.1371/journal.pbio.1001125 PMC3156686

[bib10] Churchill G. A. Doerge R. W., 1994 Empirical threshold values for quantitative trait mapping. Genetics 138: 963–971 7851788 10.1093/genetics/138.3.963 PMC1206241

[bib11] Conesa A. Götz S. Garcia-Gomez J. M. Terol J. Talon M., 2005 Blast2GO: a universal tool for annotation, visualization and analysis in functional genomics research. Bioinformatics 21: 3674–3676 16081474 10.1093/bioinformatics/bti610

[bib12] Conesa A. Götz S., 2011 Blast2GO: A comprehensive suite for functional analysis in plant genomics. Int. J. Plant Genomics 2008: 1–13 10.1155/2008/619832 PMC2375974 18483572

[bib13] Dao T. T. H. Linthorst H. J. M. Verpoorte R., 2011 Chalcone synthase and its functions in plant resistance. Phytochem. Rev. 10: 397–412 21909286 10.1007/s11101-011-9211-7 PMC3148432

[bib14] Dobzhansky T., 1970 Genetics of the Evolutionary Process. Columbia University Press, New York

[bib15] Dooner H. K. He L. M., 2008 Maize genome structure variation: Interplay between retrotransposon polymorphisms and genic recombination. Plant Cell 20: 249–258 18296625 10.1105/tpc.107.057596 PMC2276454

[bib16] Drouaud J. Camilleri C. Bourguignon P.-Y. Canaguier A. Bérard A., 2006 Variation in crossing-over rates across chromosome 4 of *Arabidopsis thaliana* reveals the presence of meiotic recombination “hot spots.” Genome Res. 16: 106–114 16344568 10.1101/gr.4319006 PMC1356134

[bib17] Elshire R. J. J. C., Glaubitz Q. Sun J. A. Poland K. Kawamoto, 2011 A robust, simple genotyping-by-sequencing (GBS) approach for high diversity species. PLoS ONE 6: e19379 21573248 10.1371/journal.pone.0019379 PMC3087801

[bib18] Fan, L., Z.-Y. Ni, and X.-Y. Hao, 2008 *Genes of Phenylpropanoid Pathway Cloning and Expression in Developing Cotton Fiber* Direct submission, Institute of Nuclear and Biological Technologies, Xinjiang Academy of Agricultural Sciences, Wulumoqi, China.

[bib19] Feldman M. W. Christiansen F. B. Brooks L. D., 1980 Evolution of recombination in a constant environment. Proc. Natl. Acad. Sci. USA 77: 4838–4841 16592864 10.1073/pnas.77.8.4838 PMC349943

[bib20] Feng J. Long Y. Shi L. Shi J. Q. Barker G., 2012 Characterization of metabolite quantitative trait loci and metabolic networks that control glucosinolate concentration in the seeds and leaves of *Brassica napus*. New Phytol. 193: 96–108 21973035 10.1111/j.1469-8137.2011.03890.x

[bib21] Fishman L. Kelly A. J. Willis J. H., 2002 Minor quantitative trait loci underlie floral traits associated with mating system divergence in *Mimulus guttatus*. Evolution 56: 2138–2155 12487345 10.1111/j.0014-3820.2002.tb00139.x

[bib22] Fishman L. Willis J. Wu C. Lee Y.-W., 2014 Comparative linkage maps reveal that fission, not polyploidy, underlies chromosome number doubling within the monkeyflowers (Mimulus; Phrymaceae). Heredity (Edinb). (in press). 10.1038/hdy.2013.143 PMC3998785 24398885

[bib64] Fraser C. M. Chapple C., 2011 The phenylpropanoid pathway in Arabidopsis. The Arabidopsis Book 9: e0152, /10.1199/tab.0152 22303276 10.1199/tab.0152 PMC3268504

[bib23] Friedman J. Willis J. H., 2013 Major QTLs for critical photoperiod and vernalization underlie extensive variation in flowering in the *Mimulus guttatus* species complex. New Phytol. 199: 571–583 23600522 10.1111/nph.12260

[bib24] Gai, Y., S. Liu, N. Chao, and X. Jiang, 2013 *Monolignon Biosynthesis Key Gene* Direct submission, College of Biological Sciences and Biotechnology, Beijing Forestry University, Beijing, China.

[bib25] Götz S. Garcia-Gomez J. M. Terol J. Williams T. D. Nagaraj S. H., 2008 High-throughput functional annotation and data mining with the Blast2GO suite. Nucleic Acids Res. 36: 3420–3435 18445632 10.1093/nar/gkn176 PMC2425479

[bib26] Götz S. Arnold R. Sebastian-Leon P. Martin-Rodriguez S. Tischler P., 2011 B2G-FAR, a species centered GO annotation repository. Bioinformatics 27: 919–924 21335611 10.1093/bioinformatics/btr059 PMC3065692

[bib27] Goodstein D. M. Shu S. Howson R. Neupane R. Hayes R. D., 2012 Phytozome: a comparative platform for green plant genomics. Nucleic Acids Res. 40: D1178–D1186 22110026 10.1093/nar/gkr944 PMC3245001

[bib28] Grubb C. D. Abel S., 2006 Glucosinolate metabolism and its control. Trends Plant Sci. 11: 89–100 16406306 10.1016/j.tplants.2005.12.006

[bib29] Halkier B. A. Gershenzon J., 2006 Biology and biochemistry of glucosinolates. Annu. Rev. Plant Biol. 57: 303–333 16669764 10.1146/annurev.arplant.57.032905.105228

[bib30] Hall M. C. Willis J. H., 2006 Divergent selection on flowering time contributes to local adaptation in *Mimulus guttatus* populations. Evolution 60: 2466–2477 17263109

[bib31] Hellsten U. Wright K. M. Jenkins J. Shu S. Yuan Y., 2013 Fine-scale variation in meiotic recombination in *Mimulus* inferred from population shotgun sequencing. Proc. Natl. Acad. Sci. USA 110: 19478–19482 24225854 10.1073/pnas.1319032110 PMC3845195

[bib32] Henery M. L. Moran G. F. Wallis I. R. Foley W. J., 2007 Identification of quantitative trait loci influencing foliar concentrations of terpenes and formylated phloroglucinol compounds in *Eucalyptus nitens*. New Phytol. 176: 82–95 17696979 10.1111/j.1469-8137.2007.02159.x

[bib33] Holeski L. M., 2007 Within and between generation phenotypic plasticity in trichome density of *Mimulus guttatus*. J. Evol. Biol. 20: 2092–2100 17903186 10.1111/j.1420-9101.2007.01434.x

[bib34] Holeski L. M. Chase-Alone R. Kelly J. K., 2010 The genetics of phenotypic plasticity in plant defense: trichome production in *Mimulus guttatus*. Am. Nat. 175: 391–400 20180699 10.1086/651300

[bib35] Holeski L. M. Keefover-Ring K. Bowers M. D. HarnEnz Z. T. Lindroth R. L., 2013 Patterns of phytochemical variation in *Mimulus guttatus* (yellow monkeyflower). J. Chem. Ecol. 39: 525–2536 23468225 10.1007/s10886-013-0270-7

[bib36] Iwata H. Ninomiya S., 2006 AntMap: constructing genetic linkage maps using an ant colony optimization algorithm. Breed. Sci. 56: 371–377

[bib37] Jensen-Seaman M. I. Furey T. S. Payseur B. A. Lu Y. Roskin K. M., 2004 Comparative recombination rates in the rat, mouse, and human genomes. Genome Res. 14: 528–538 15059993 10.1101/gr.1970304 PMC383296

[bib38] Kim J.-S. Islam-Faridi M. N. Klein P. E. Stelly D. M. Price H. J., 2005 comprehensive molecular cytogenetic analysis of sorghum genome architecture: distribution of euchromatin, heterochromatin, genes and recombination in comparison to rice. Genetics 171: 1963–1976 16143604 10.1534/genetics.105.048215 PMC1456119

[bib39] King M., 1993 Species Evolution: The Role of Chromosomal Change. Cambridge University Press, Cambridge, England

[bib40] Kliebenstein D. J. Gershenzon J. Mitchell-Olds T., 2001 Comparative quantitative trait loci mapping of aliphatic, indolic and benzylic glucosinolate production in *Arabidopsis thaliana* leaves and seeds. Genetics 159: 359–370 11560911 10.1093/genetics/159.1.359 PMC1461795

[bib41] Knaggs A. R., 2003 The biosynthesis of shikimate metabolites. Nat. Prod. Rep. 20: 119–136 12636087 10.1039/b100399m

[bib42] Kondrashov A. S., 1982 Selection against harmful mutations in large sexual and asexual populations. Genet. Res. 40: 325–332 7160619 10.1017/s0016672300019194

[bib43] Kondrashov A. S., 1988 Deleterious mutations and the evolution of sexual reproduction. Nature 336: 435–440 3057385 10.1038/336435a0

[bib44] Lauvergeat V. Lacomme C. Lacombe E. Lasserre E. Roby D., 2001 Two cinnamoyl-CoA reductase (CCR) genes from *Arabidopsis thaliana* are differentially expressed during development and in response to infection with pathogenic bacteria. Phytochemistry 57: 1187–1195 11430991 10.1016/s0031-9422(01)00053-x

[bib45] Lenormand T. Otto S. P., 2000 The evolution of recombination in a heterogenous environment. Genetics 156: 423–438 10978305 10.1093/genetics/156.1.423 PMC1461255

[bib46] Li H. Durbin R., 2009 Fast and accurate short read alignment with Burrows-Wheeler transform. Bioinformatics 25: 1754–1760 19451168 10.1093/bioinformatics/btp324 PMC2705234

[bib47] Li W. L. Faris J. D. Chittoor J. M. Leach J. E. Hulbert S. H., 1999 Genomic mapping of defense response genes in wheat. Theor. Appl. Genet. 98: 226–233

[bib48] Lowry D. B. Willis J. H., 2010 A widespread chromosomal inversion polymorphism contributes to a major life-history transition, local adaptation, and reproductive isolation. PLoS ONE 8: e1000500 10.1371/journal.pbio.1000500 PMC2946948 20927411

[bib49] Lowry D. B. Sheng C. C. Lasky J. R. Willis J. H., 2012 Five anthocyanin polymorphisms are associated with an R2R3-MYB cluster in *Mimulus guttatus* (Phrymaceae). Am. J. Bot. 99: 82–91 22186184 10.3732/ajb.1100285 PMC11061637

[bib50] McKenna A. Hanna M. Banks E. Sivachenko A. Cibulskis K., 2010 The Genome Analysis Toolkit: a MapReduce framework for analyzing next-generation DNA sequencing data. Genome Res. 20: 1297–1303 20644199 10.1101/gr.107524.110 PMC2928508

[bib51] Mølgaard P., 1986 Population genetics and geographical distribution of caffeic acid esters in leaves of *Plantago major* in Denmark. J. Ecol. 74: 1127–1137

[bib52] Mølgaard P. Ravn H., 1988 Evolutionary aspects of caffeoyl ester distribution in dicotyledons. Phytochemistry 27: 2411–2421

[bib53] Nachman M. W., 2002 Variation in recombination rate across the genome: evidence and implications. Curr. Opin. Genet. Dev. 12: 657–663 12433578 10.1016/s0959-437x(02)00358-1

[bib54] O’Reilly-Wapstra J. M. Freeman J. S. Davies N. W. Vaillancourt R. E. Fitzgerald H., 2011 Quantitative trait loci for foliar terpenes in a global eucalypt species. Tree Genet. Genomes 7: 485–498

[bib55] Poland J. A. Rife T. W., 2012 Genotyping-by-sequencing for plant breeding and genetics. Plant Genome 5: 92–102

[bib56] R Core Team, 2012 R: A language and environment for statistical computing. R Foundation for Statistical Computing. R Core Team, Vienna, Austria.

[bib57] Roze D. Lenormand T., 2005 Self-fertilization and the evolution of recombination. Genetics 170: 841–857 15781706 10.1534/genetics.104.036384 PMC1450426

[bib58] Schnable P. S. Ware D. Fulton R. S. Stein J. C. Wei F., 2009 The B73 maize genome: complexity, diversity, and dynamics. Science 326: 1112–1115 19965430 10.1126/science.1178534

[bib59] Slotte T. Hazzouri K. M. Stern D. Andolfatto P. Wright S. I., 2012 Genetic architecture and adaptive significance of the selfing syndrome in capsella. Evolution 66: 1360–1374 22519777 10.1111/j.1558-5646.2011.01540.x PMC5063048

[bib60] Smit F. Dubery L. A., 1997 Cell wall reinforcement in cotton hypocotyls in response to a *Verticillium dahliae* elicitor. Phytochemistry 44: 811–815

[bib61] Stebbins G. L., 1958 The inviability, weakness, and sterility of interspecific hybrids. Adv. Genet. 9: 147–215 13520442 10.1016/s0065-2660(08)60162-5

[bib62] Tuskan G. A. DiFazio S. Jansson S. Bohlmann J. Grigoriev I., 2006 The genome of black cottonwood, *Populus trichocarpa* (Torr. & Gray). Science 313: 1596–1604 16973872 10.1126/science.1128691

[bib63] Wright S. I. Nano N. Foxe J. P. Dar V. U. N., 2008 Effective population size and tests of neutrality at cytoplasmic genes in *Arabidopsis*. Genet. Res. 90: 119–128 10.1017/S0016672307008920 18289406

