# Supplementary material for: A High-Resolution Genetic Map of Yellow Monkeyflower Identifies Chemical Defense QTLs and Recombination Rate Variation
Source: G3 (Bethesda). 2014 Mar 13;4(5):813–21. doi: 10.1534/g3.113.010124 (PMC4025480; doi:10.1534/g3.113.010124)
Supplement: Supporting Information [file supp_4_5_813__index.html]

A High-Resolution Genetic Map of Yellow Monkeyflower Identifies Chemical Defense QTLs and Recombination Rate Variation — Supporting Information 

# A High-Resolution Genetic Map of Yellow Monkeyflower Identifies Chemical Defense QTLs and Recombination Rate Variation

## Supporting Information for Holeski *et al.*, 2014

**Files in this Data Supplement:**

- Supporting Information - Figures S1-S3 and Tables S1-S2 (PDF, 1 MB)
- Figure S1 - Depiction of recombination rate variation along each chromosome. (PDF, 984 KB)
- Figure S2 - The number of genes plotted against the genetic distance for windows of ~500 kb. (PDF, 158 KB)
- Figure S3 - A graphical depiction of RIL Genotype proportions across the 14 chromosomes. (PDF, 167 KB)
- Table S2 - Results of GLM ANOVA. (PDF, 117 KB)
- Table S1 - Full genetic map and the genotype calls for 480 RILs. (.xlsx, 4 MB)
