# Supplementary material for: A High-Resolution Genetic Map of Yellow Monkeyflower Identifies Chemical Defense QTLs and Recombination Rate Variation
Source: G3 (Bethesda). 2014 Mar 13;4(5):813–21. doi: 10.1534/g3.113.010124 (PMC4025480; doi:10.1534/g3.113.010124)
Supplement: Supporting Information [file supp_g3.113.010124_010124SI.pdf]

G3·Genes | Genomes | Genetics

A high-resolution genetic map of yellow monkeyflower identifies chemical defense QTLs and recombination rate variation

Liza Holeski[*ξ†], Patrick Monnahan[*‡], Boryana Koseva[‡], Nick McCool[‡], Richard L. Lindroth[ξ], and John K. Kelly[‡]

[*] These authors contributed equally to the paper
[ξ] Dept. Entomology, University of Wisconsin, Madison
[†] Current address: Dept. Biological Sciences, Northern Arizona University
[‡] Dept. Ecology and Evolutionary Biology, University of Kansas

Corresponding author: John K. Kelly, University of Kansas, 1200 Sunnyside Ave, Lawrence, Kansas, 66045. Email: jkk@ku.edu

DOI: 10.1534/g3.113.010124

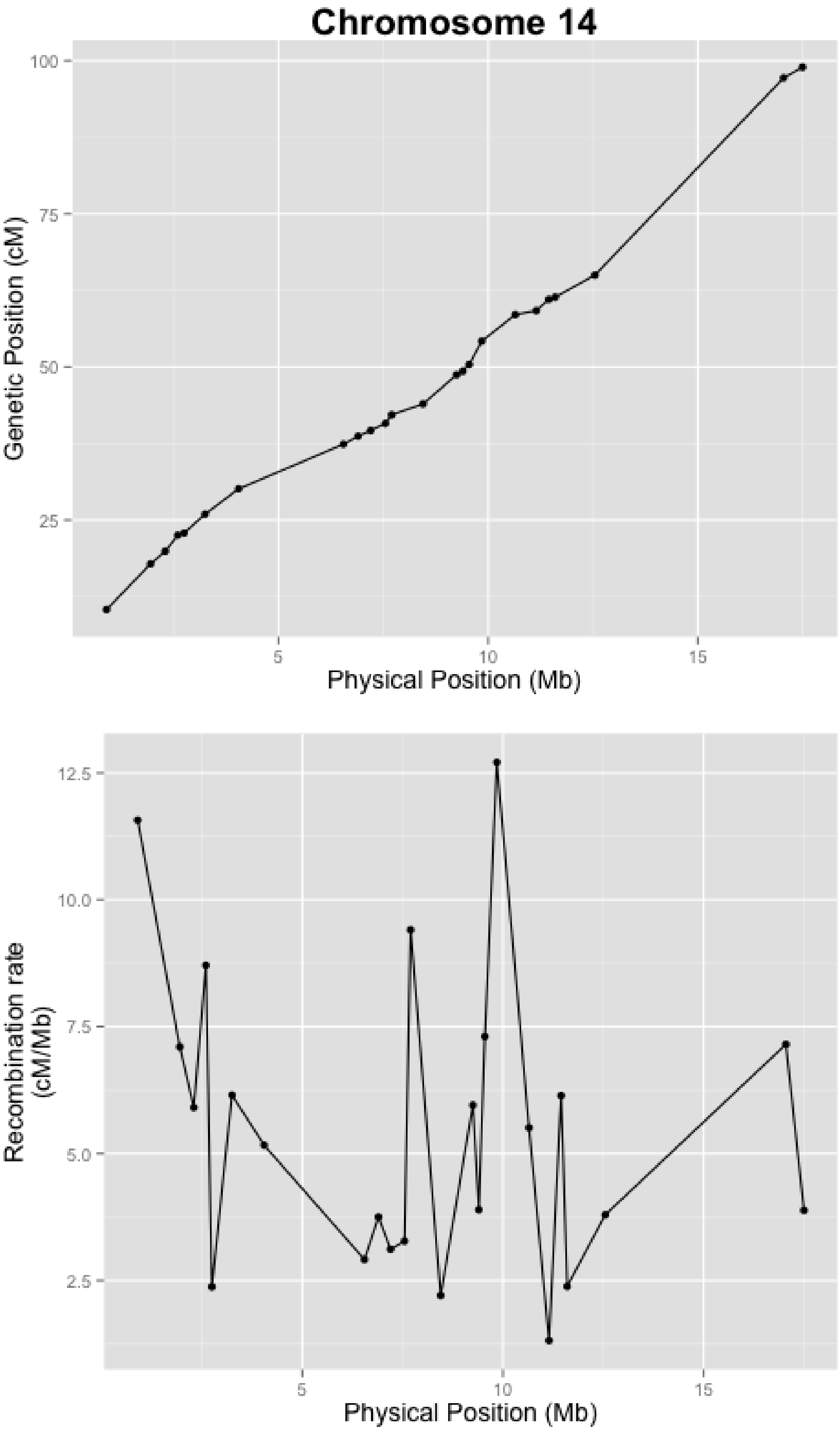
Chromosome 14
Genetic Position (cM)
100
75
50
25
5
10
15
Physical Position (Mb)
Recombination rate (cM/Mb)
12.5
10.0
7.5
5.0
2.5
5
10
15
Physical Position (Mb)

Chromosome 13

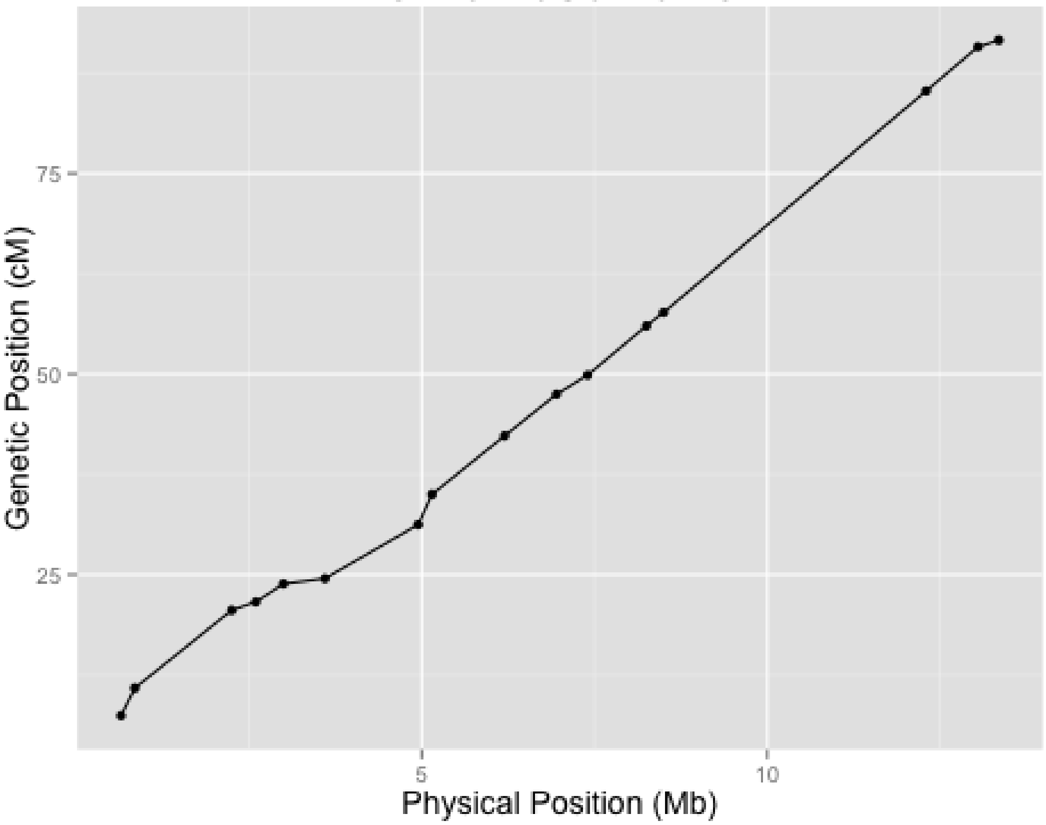

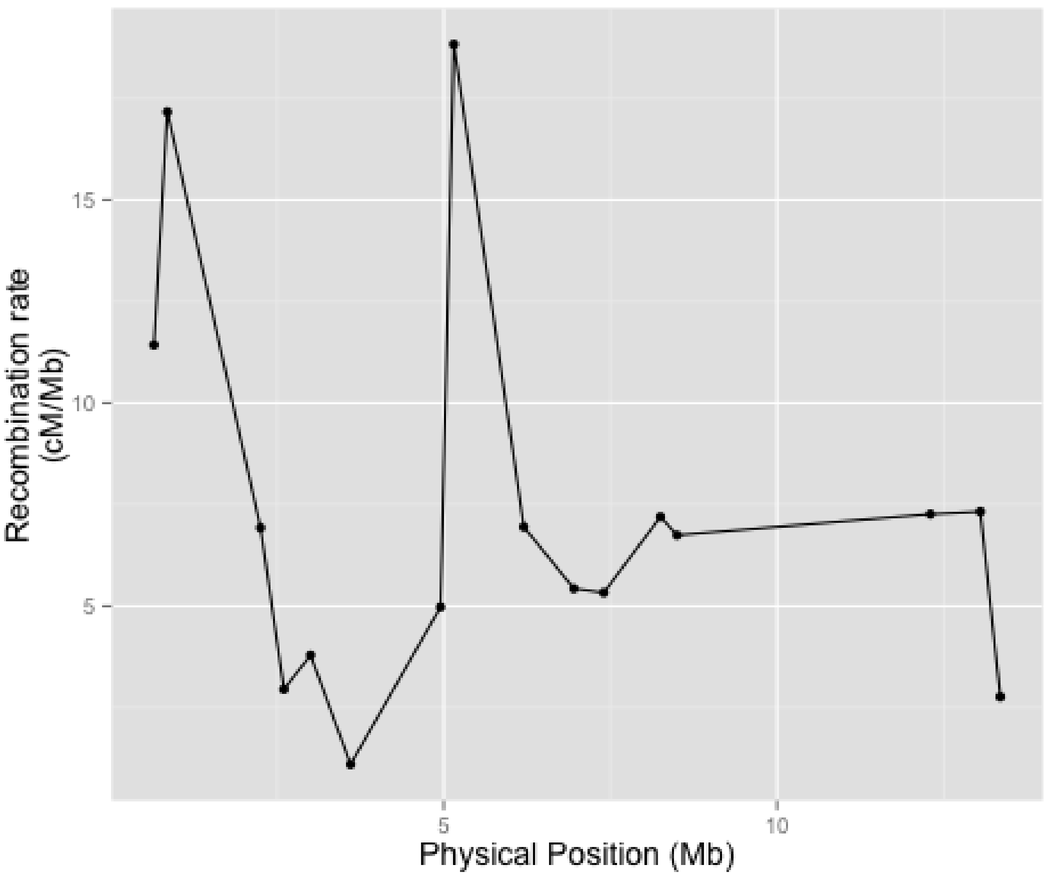

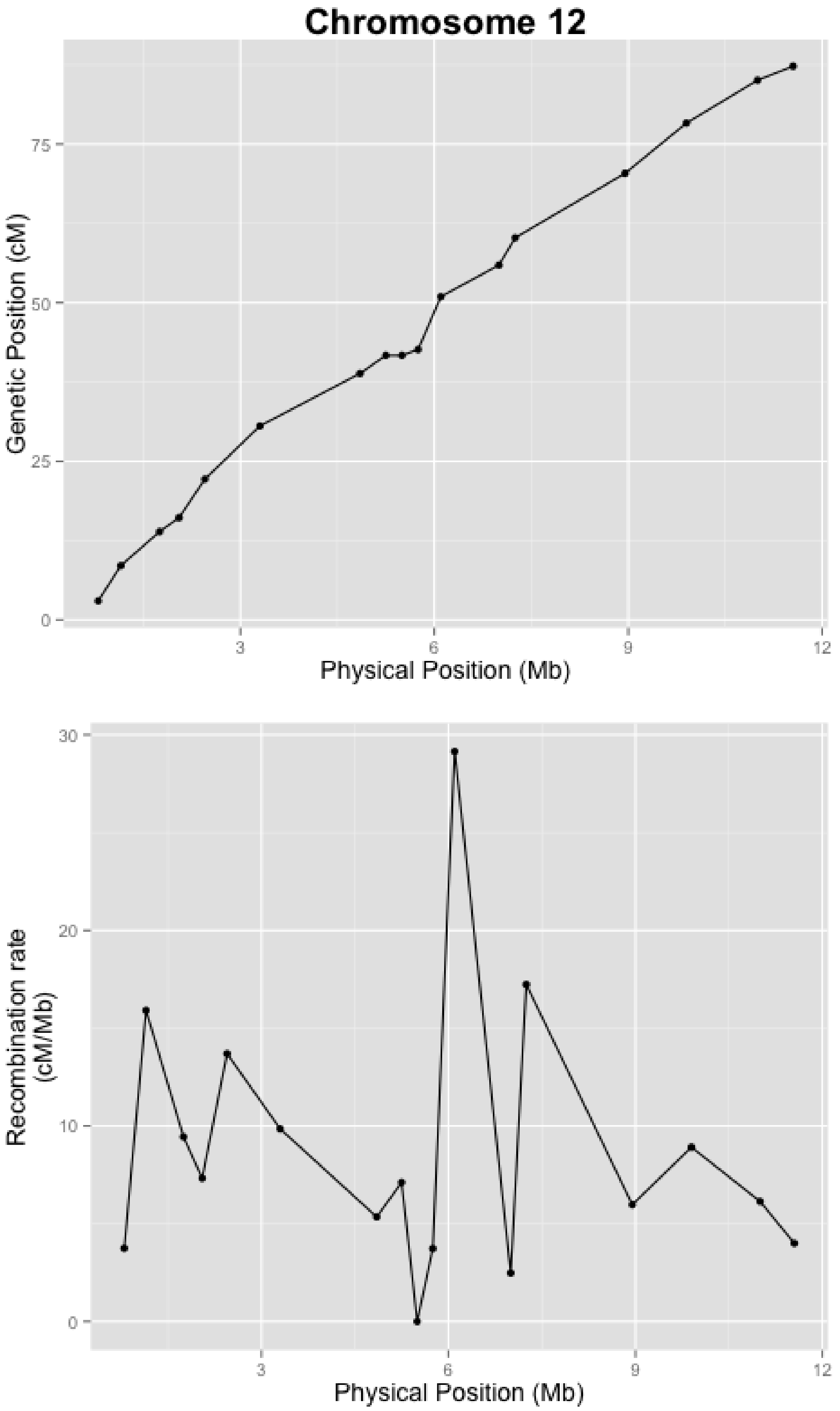
Chromosome 12
Genetic Position (cM)
0
25
50
75
Physical Position (Mb)
3
6
9
12
Recombination rate (cM/Mb)
0
10
20
30
Physical Position (Mb)
3
6
9
12

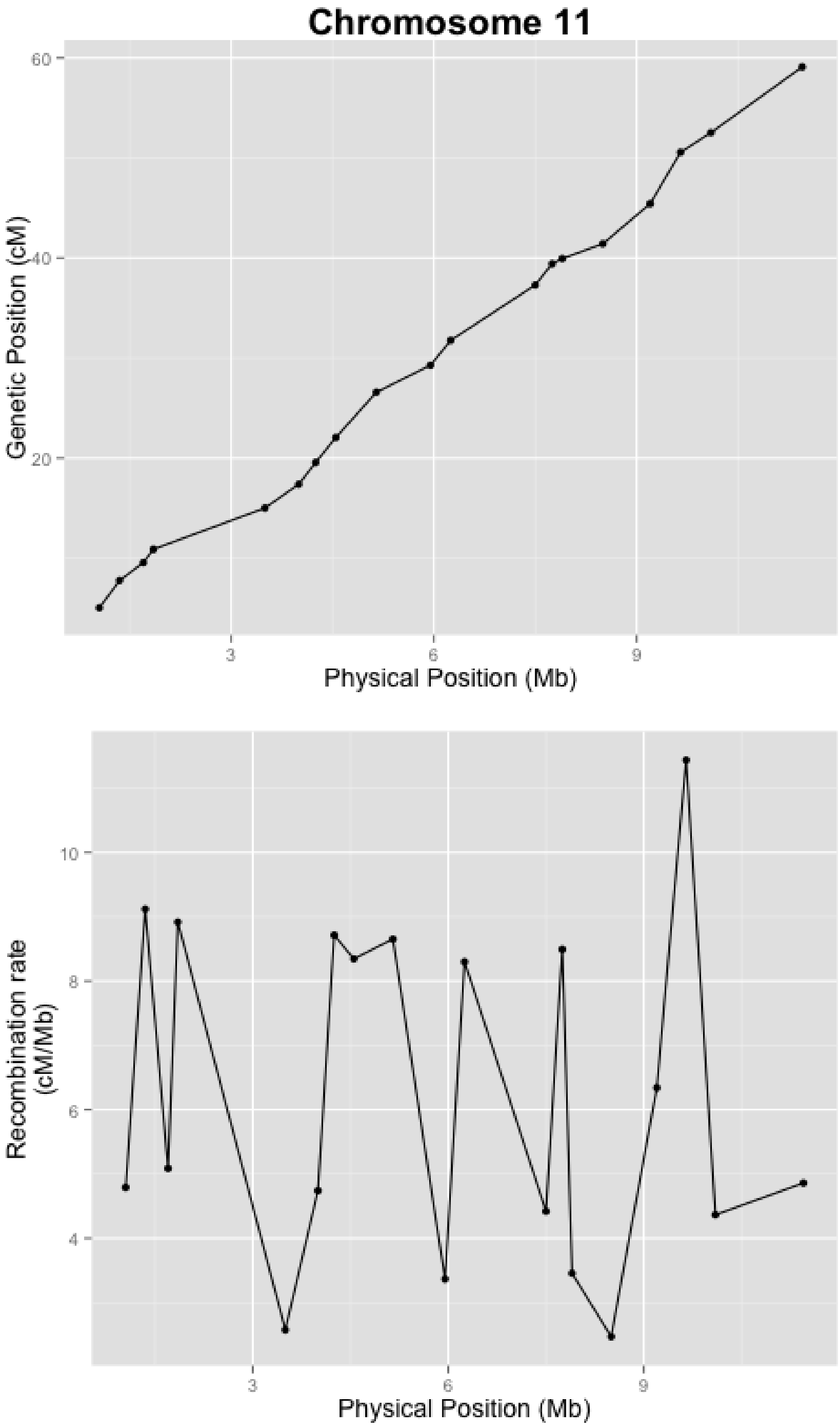
Chromosome 11
60
40
20
Genetic Position (cM)
3
6
9
Physical Position (Mb)
10
8
6
4
Recombination rate
(cM/Mb)
3
6
9
Physical Position (Mb)

Chromosome 10

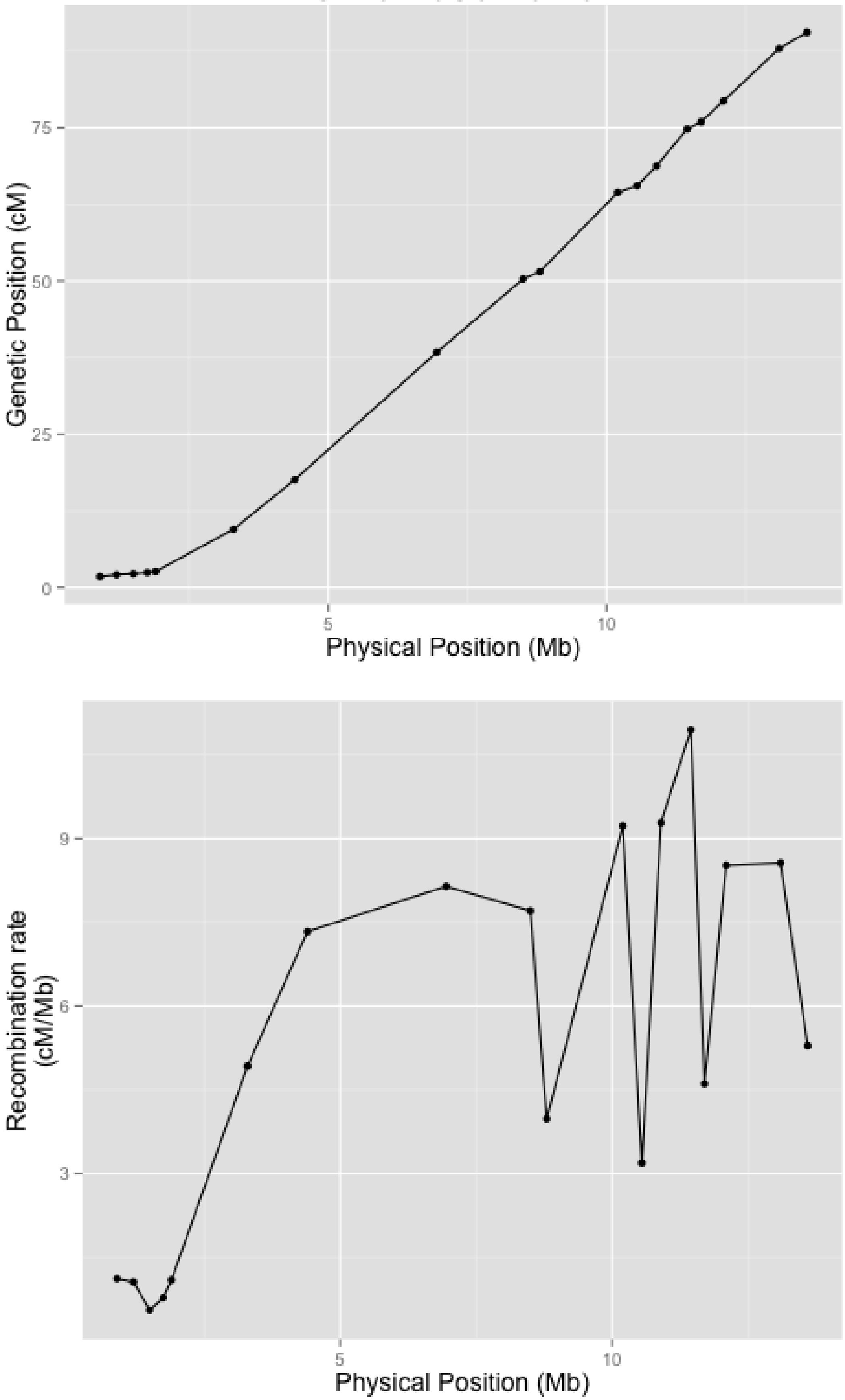

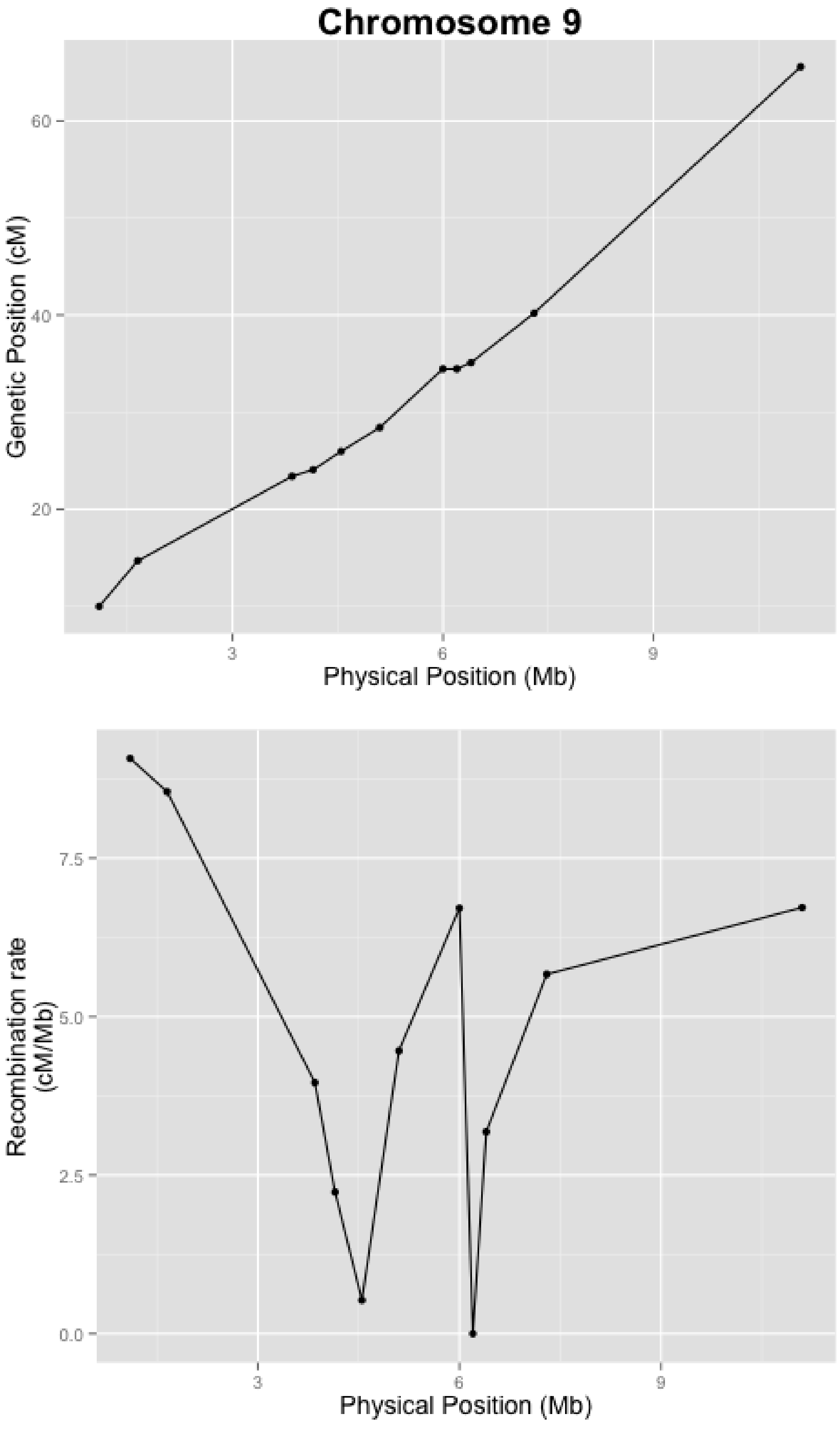
Chromosome 9
Genetic Position (cM)
60
40
20
3
6
9
Physical Position (Mb)
Recombination rate (cM/Mb)
7.5
5.0
2.5
0.0
3
6
9
Physical Position (Mb)

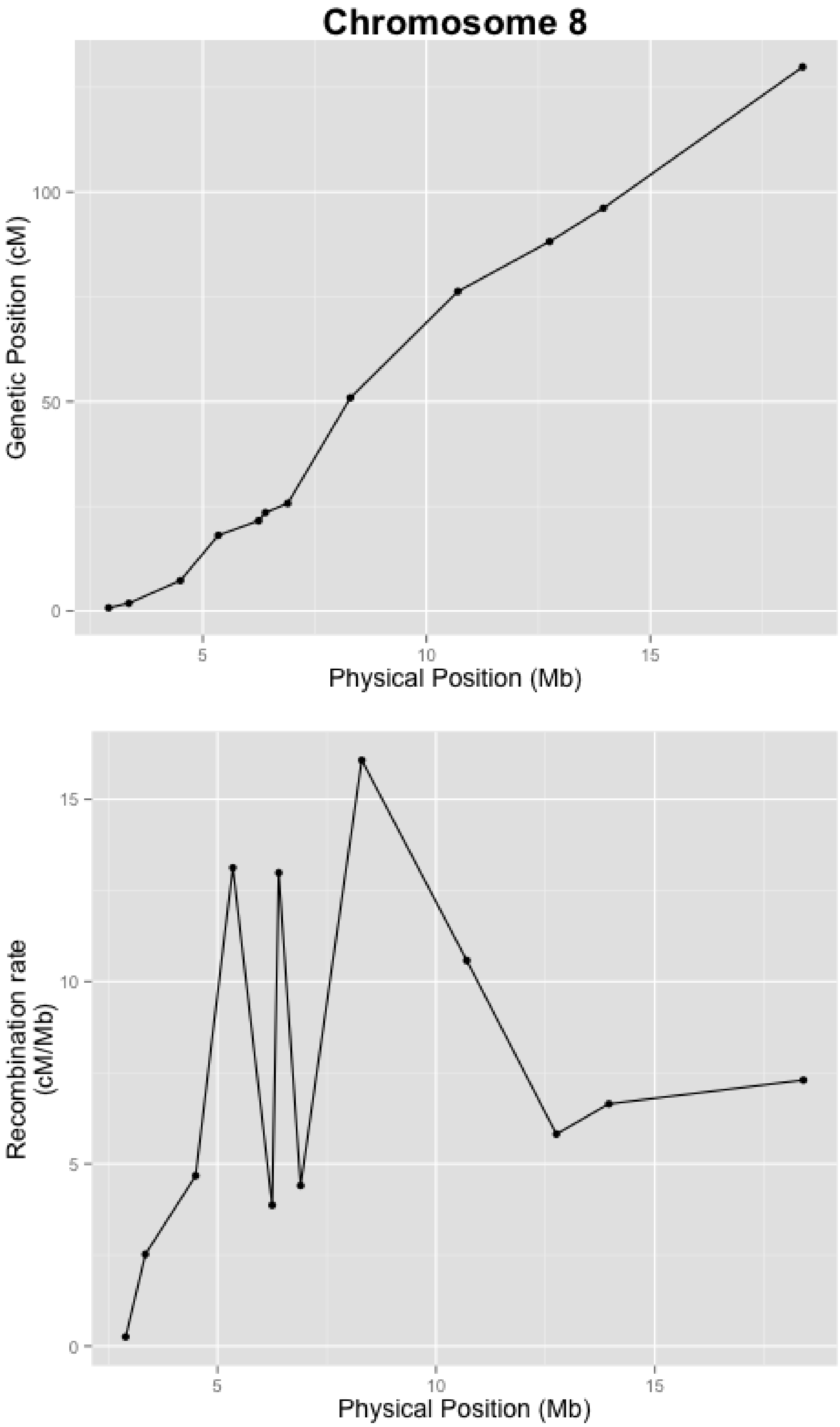
Chromosome 8
Genetic Position (cM)
100
50
0
5
10
15
Physical Position (Mb)
Recombination rate
(cM/Mb)
15
10
5
0
5
10
15
Physical Position (Mb)

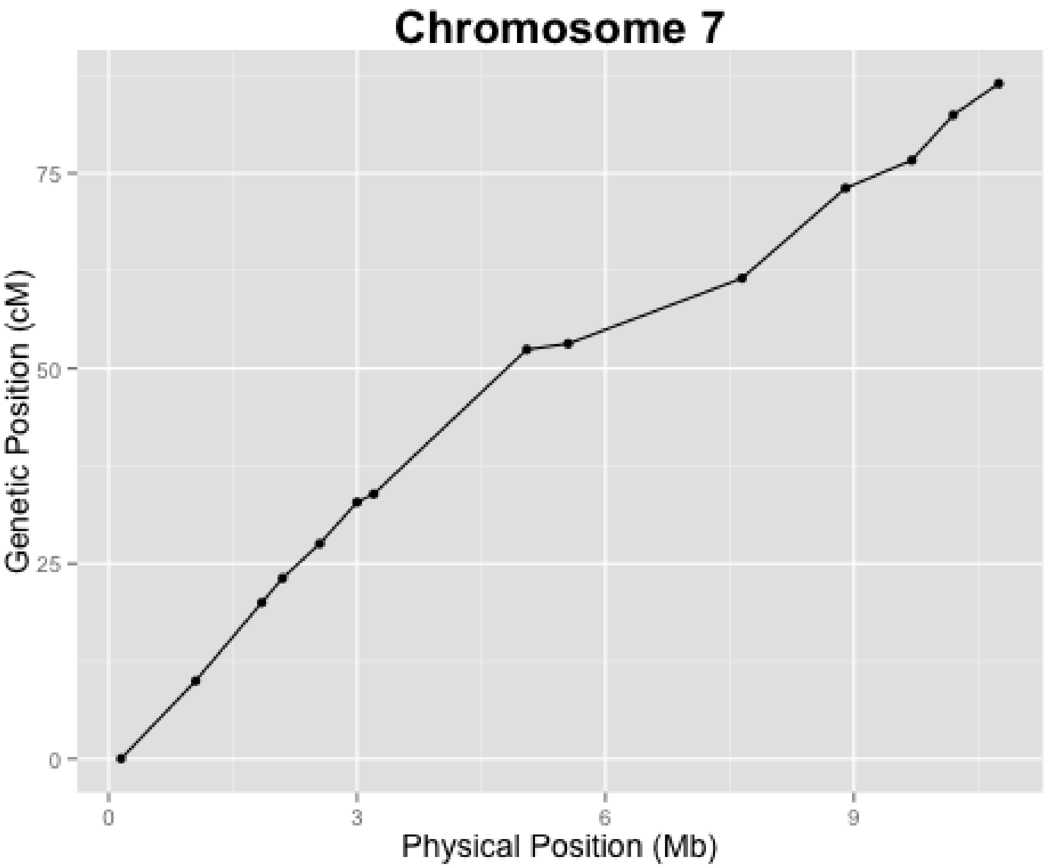
Chromosome 7
Genetic Position (cM)
75
50
25
0
0
3
6
9
Physical Position (Mb)

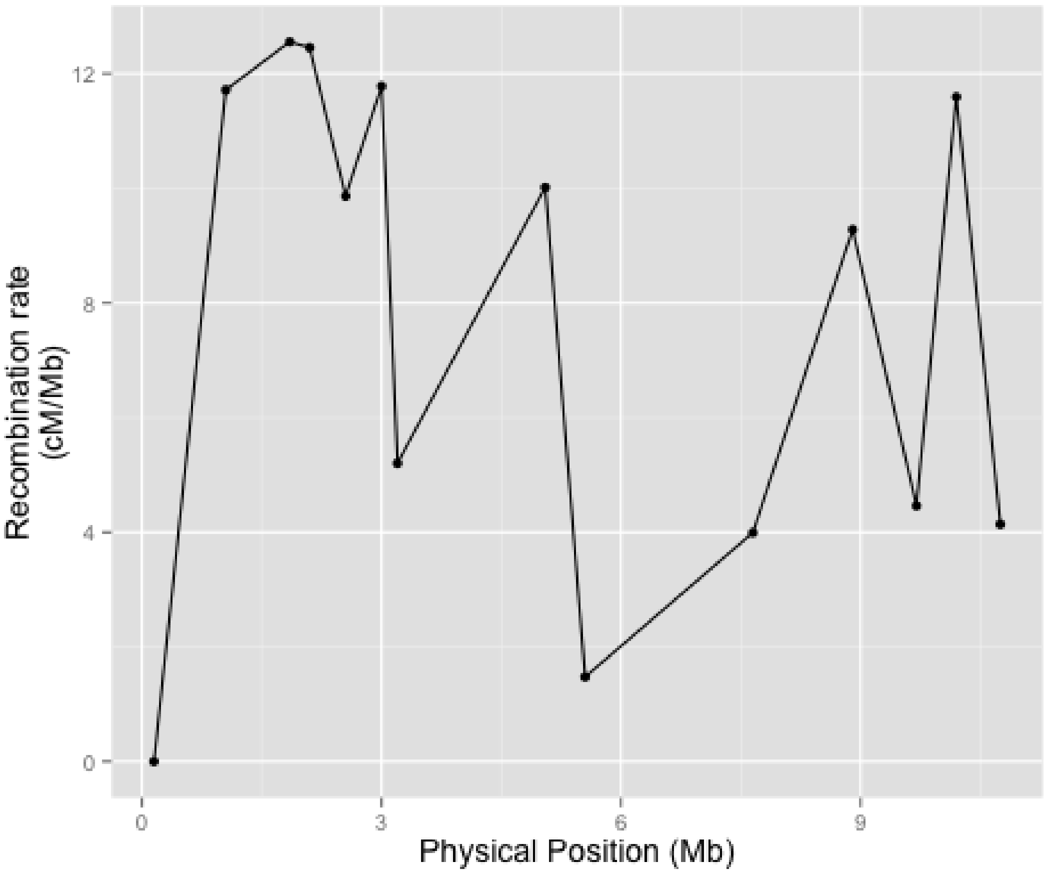
Recombination rate
(cM/Mb)
12
8
4
0
0
3
6
9
Physical Position (Mb)

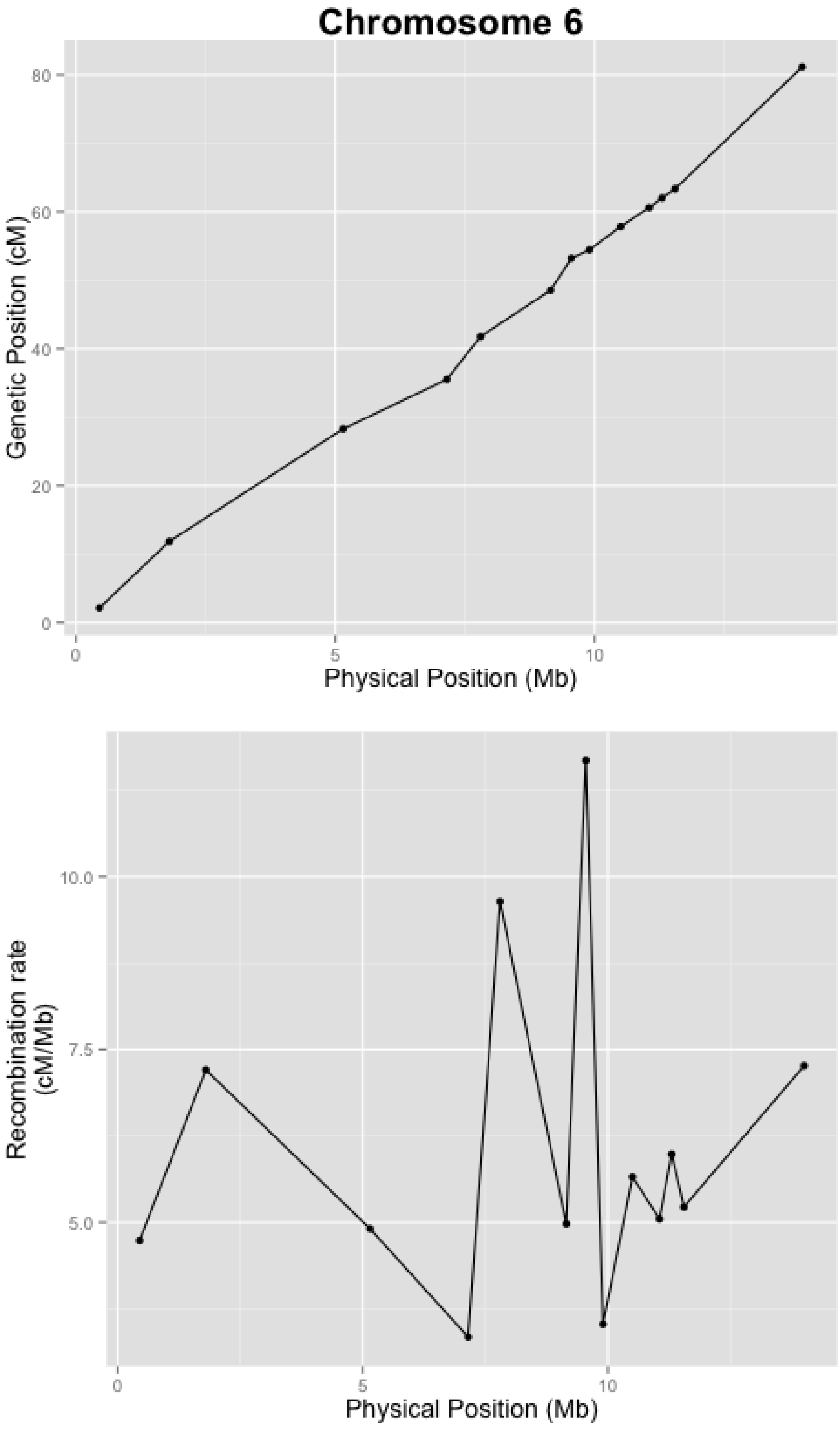
Chromosome 6
Genetic Position (cM)
0
20
40
60
80
0
5
10
Physical Position (Mb)
Recombination rate
(cM/Mb)
5.0
7.5
10.0
0
5
10
Physical Position (Mb)

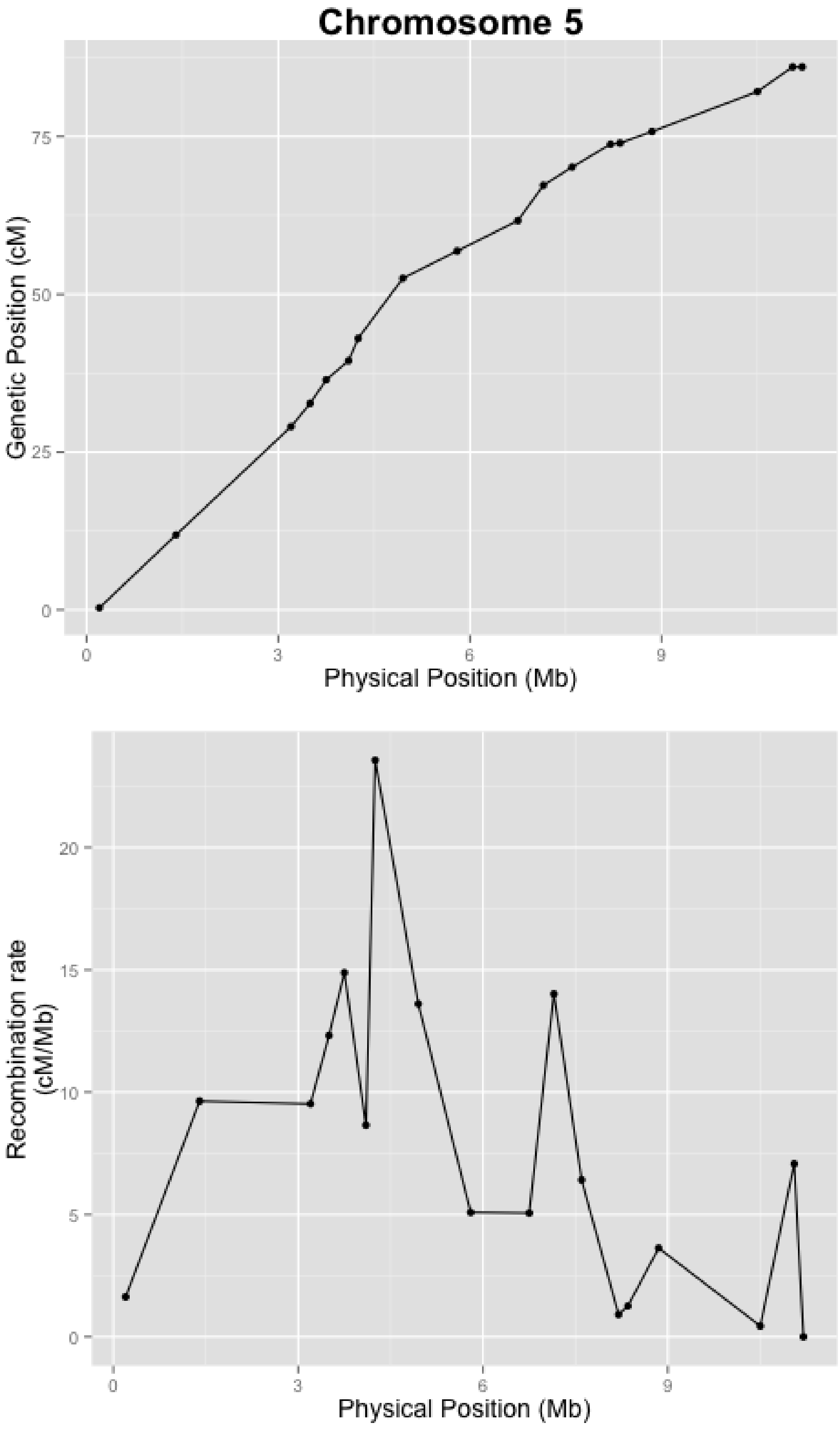
Chromosome 5
Genetic Position (cM)
0
25
50
75
0
3
6
9
Physical Position (Mb)
Recombination rate
(cM/Mb)
0
5
10
15
20
0
3
6
9
Physical Position (Mb)

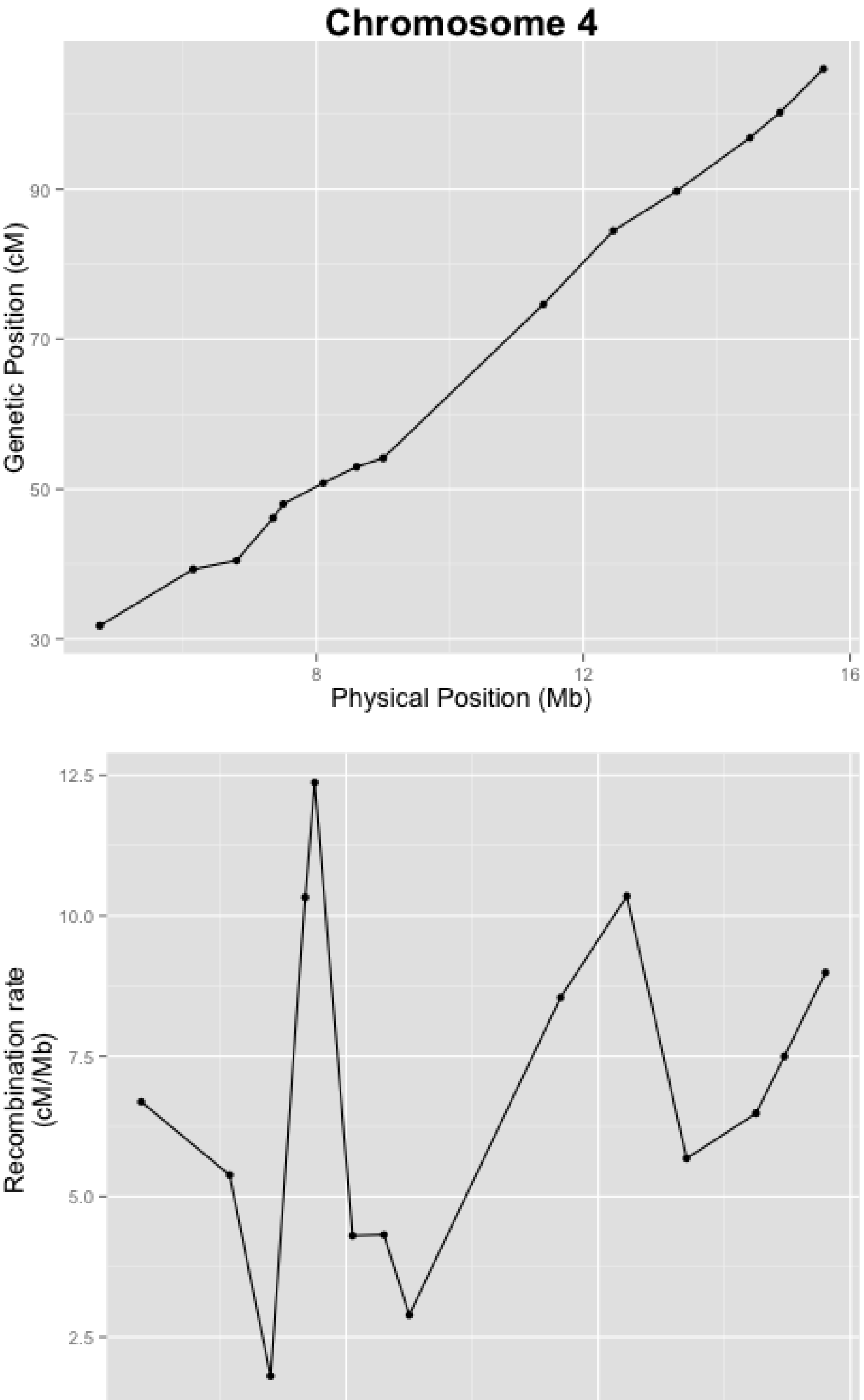
Chromosome 4
Genetic Position (cM)
30
50
70
90
Physical Position (Mb)
8
12
16
Recombination rate (cM/Mb)
2.5
5.0
7.5
10.0
12.5
Physical Position (Mb)
8
12
16

Chromosome 3

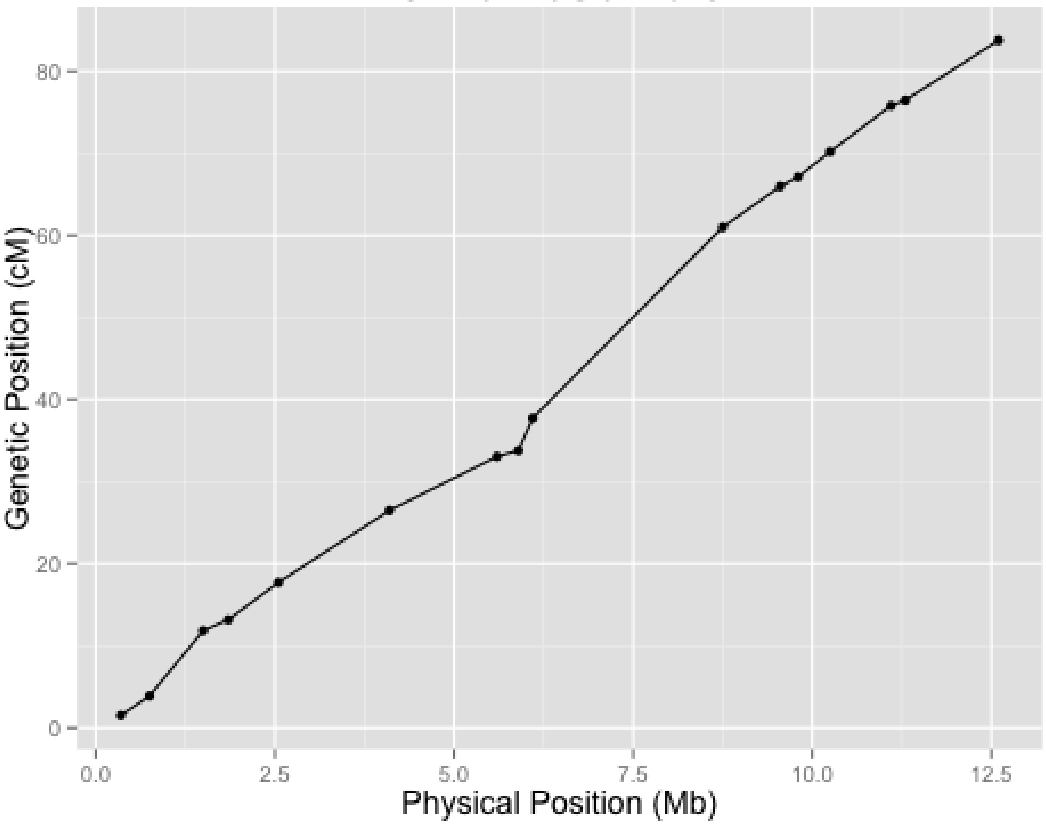

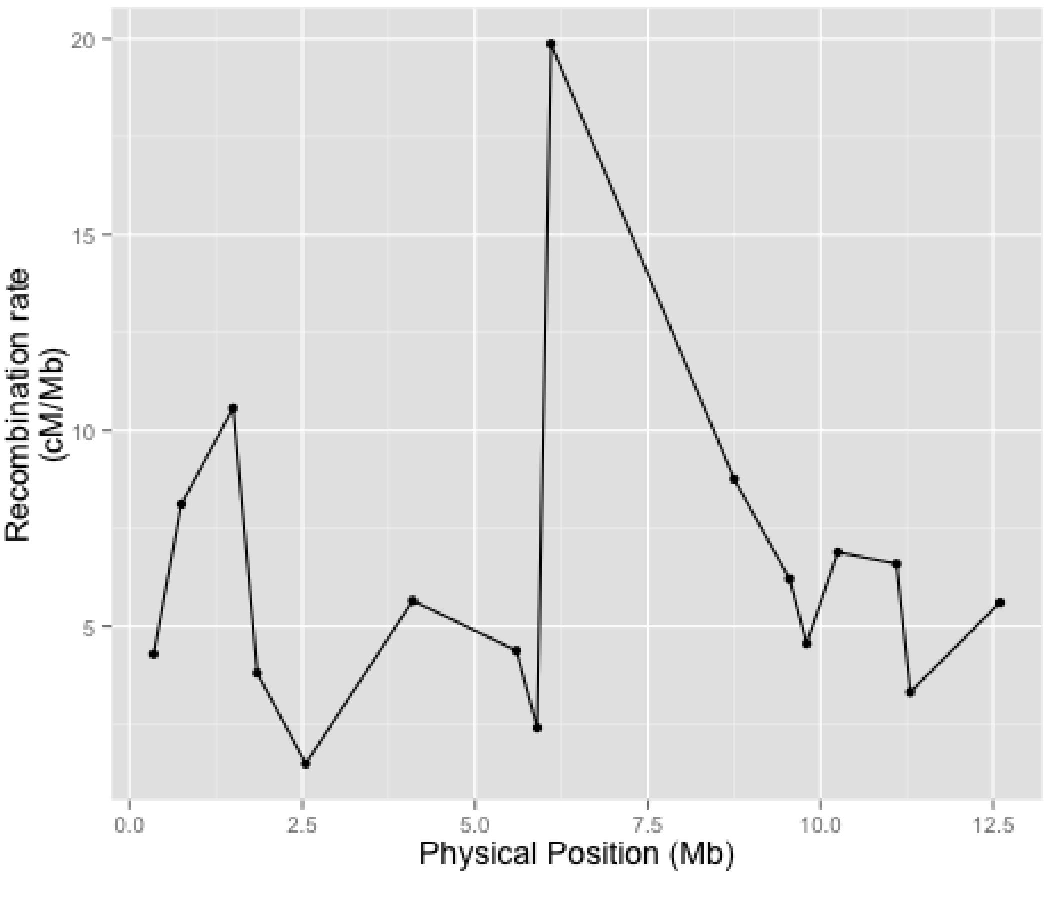

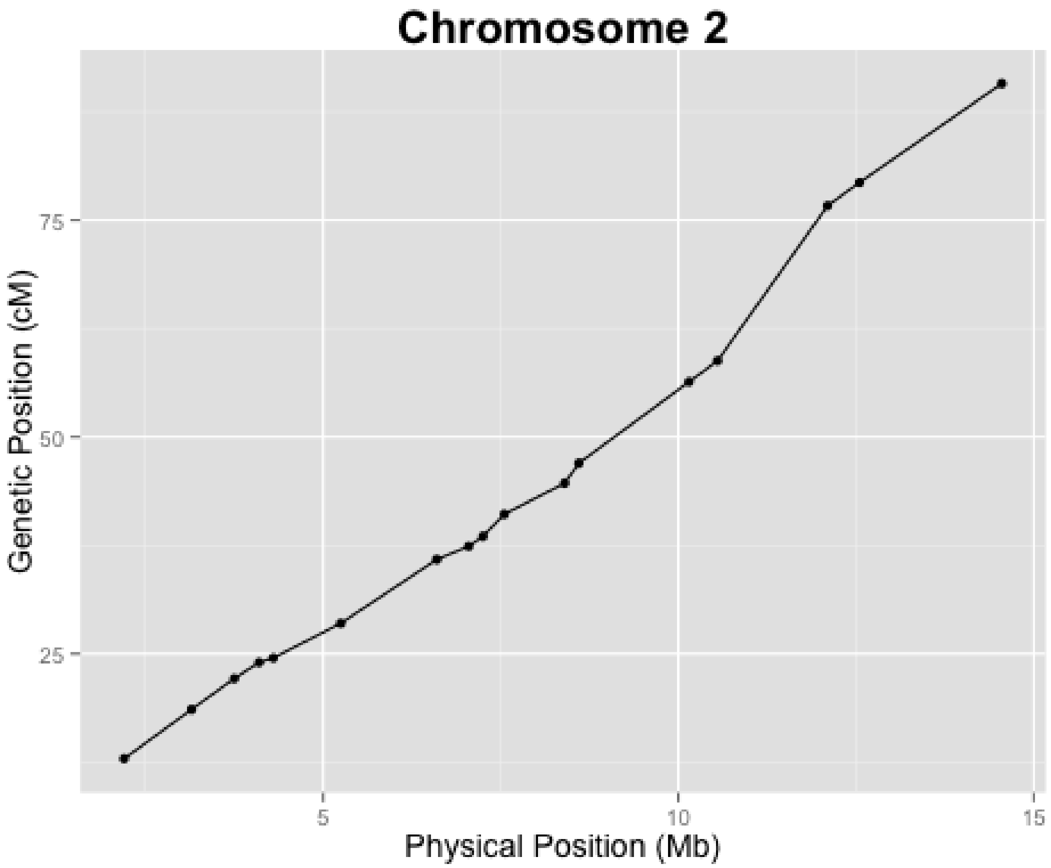

Chromosome 2
Genetic Position (cM)
75
50
25
5
10
15
Physical Position (Mb)

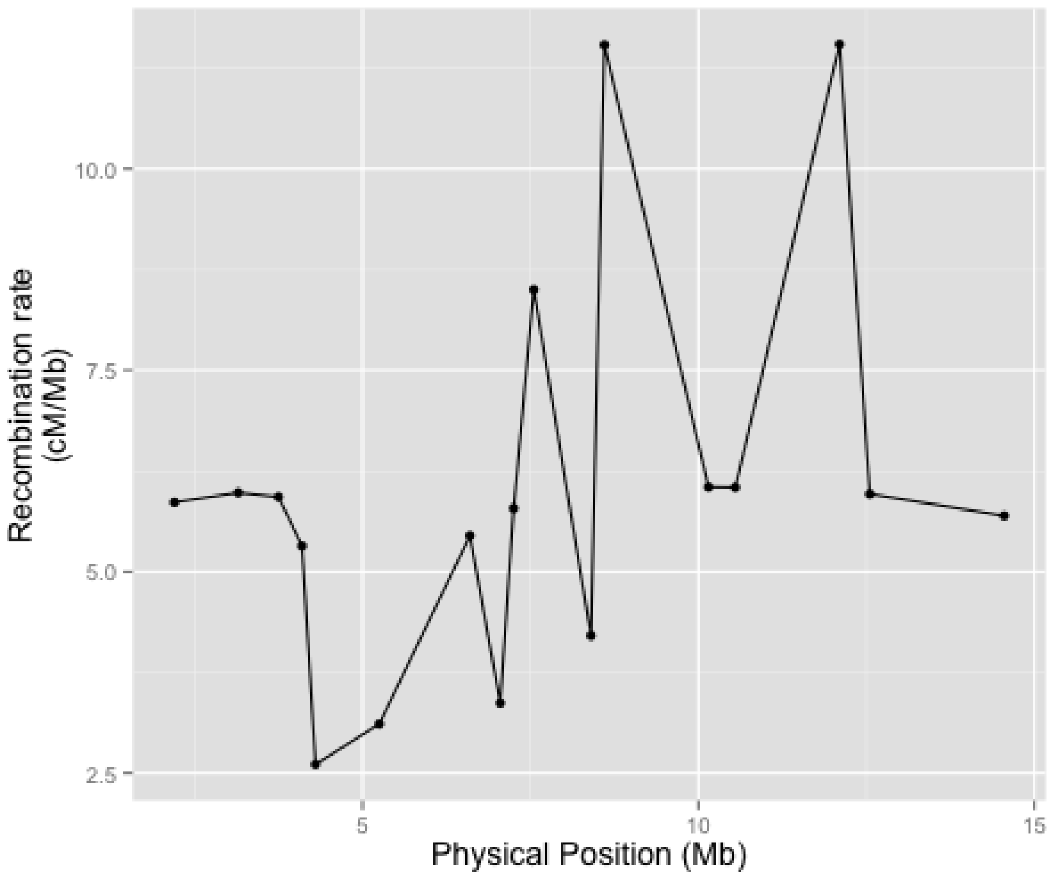

Recombination rate
(cM/Mb)
10.0
7.5
5.0
2.5
5
10
15
Physical Position (Mb)

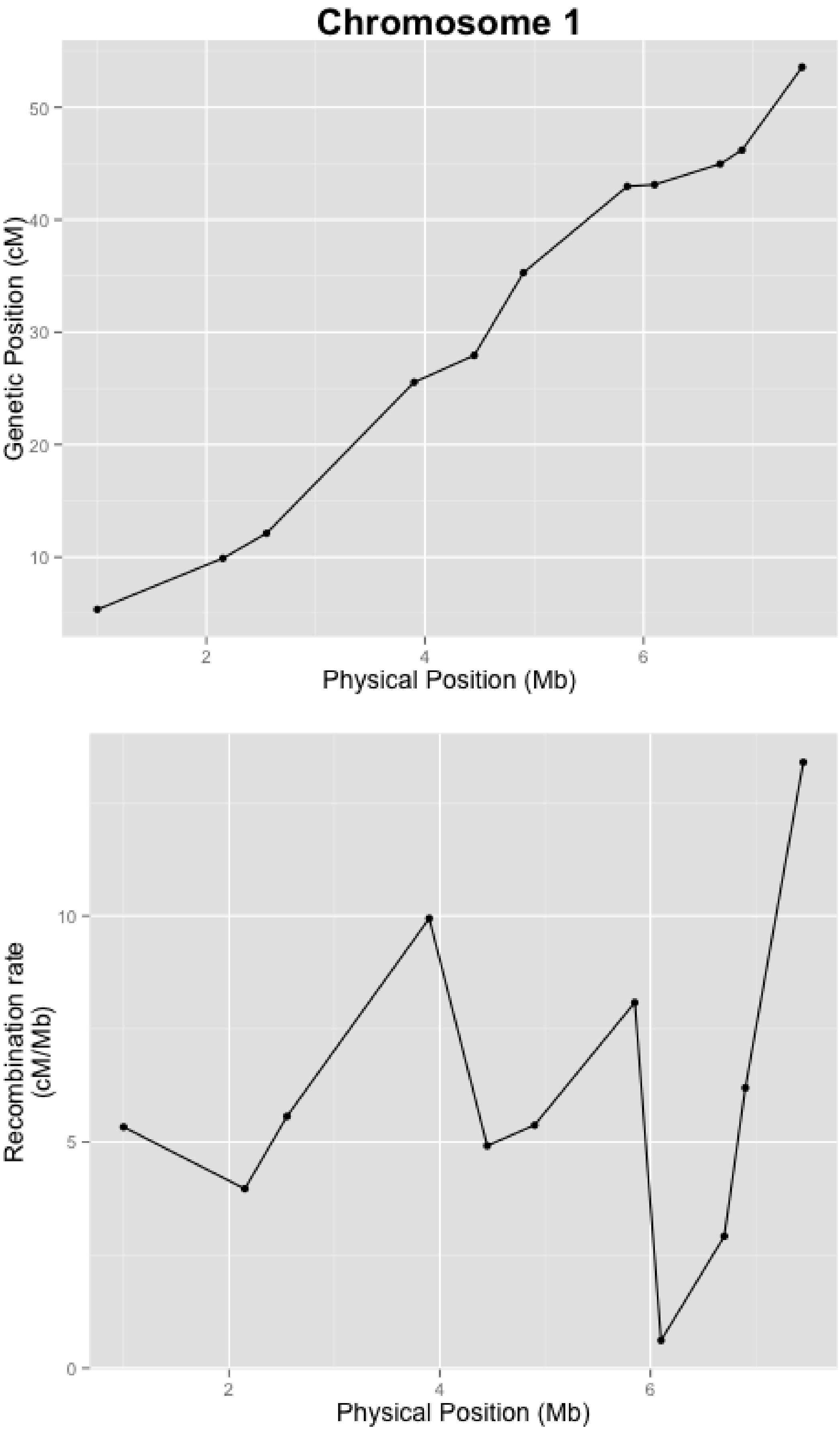
Chromosome 1
Genetic Position (cM)
50
40
30
20
10
2
4
6
Physical Position (Mb)
Recombination rate (cM/Mb)
10
5
0
2
4
6
Physical Position (Mb)

Figure S1 Depiction of recombination rate variation along each chromosome. Each point is an estimate from an entire scaffold. Scaffolds were aligned according to their order in the linkage map. Note that the apparently adjacent scaffolds are not physically adjacent as we do not account for distance between scaffolds. The distance between points corresponds to the length of the scaffolds. Only scaffolds >100 kb are depicted, and extreme observations were removed for better visualization.

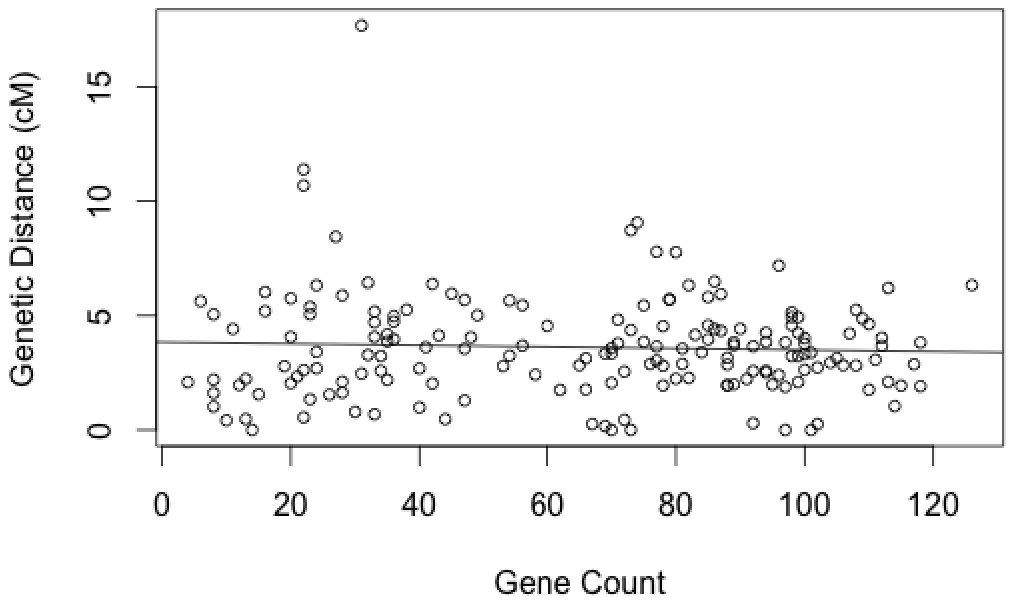

Figure S2 The number of genes plotted against the genetic distance for windows of ~500 kb. Only scaffolds >500 kb were included in the figure. The solid line is a least squares regression line indicating little evidence of a relationship.

Chromosome 1:

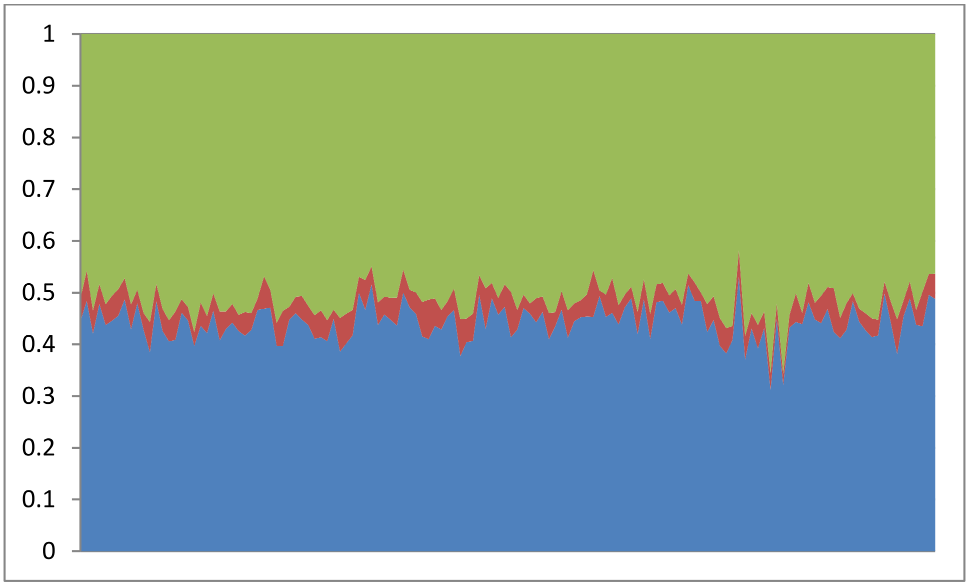

Chromosome 2:

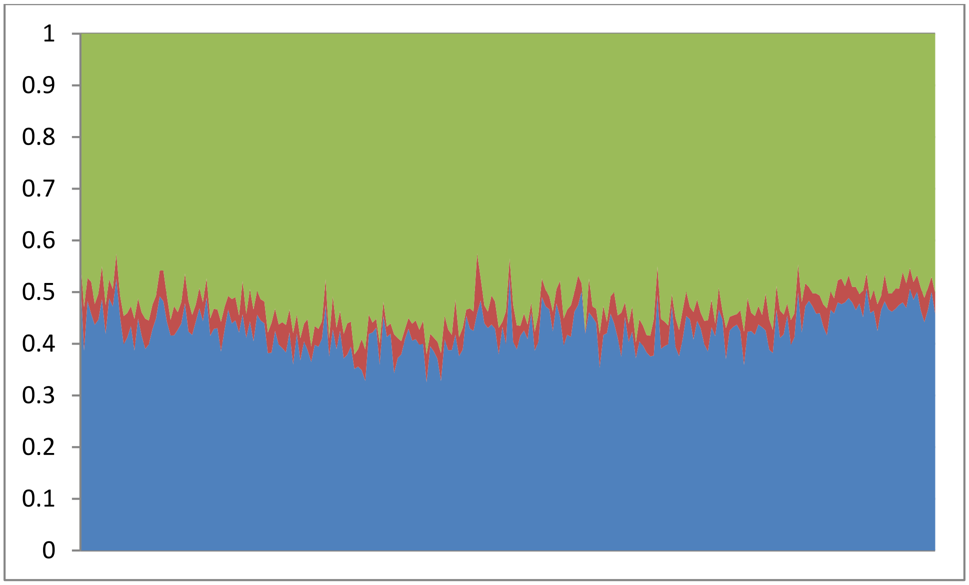

Chromosome3:

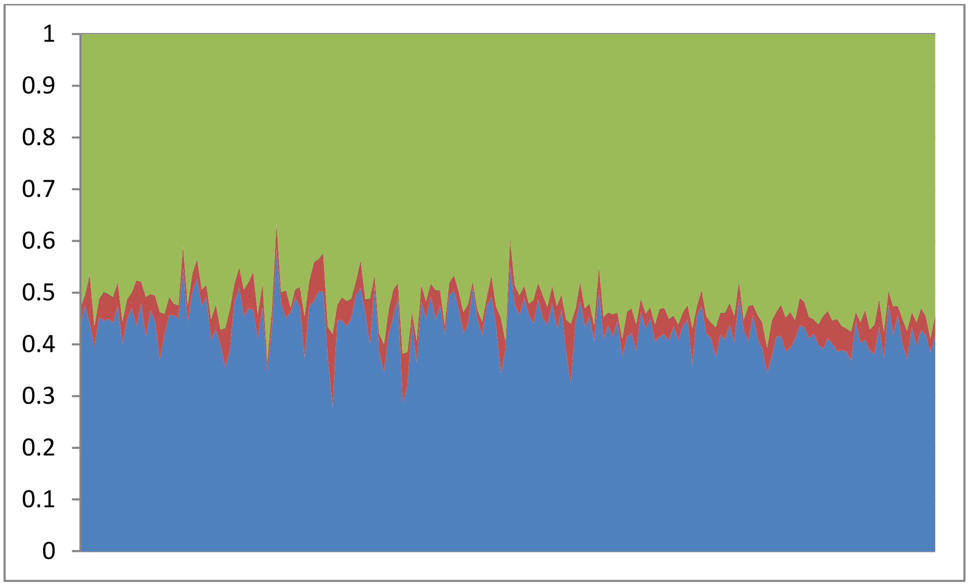

Chromosome 4:

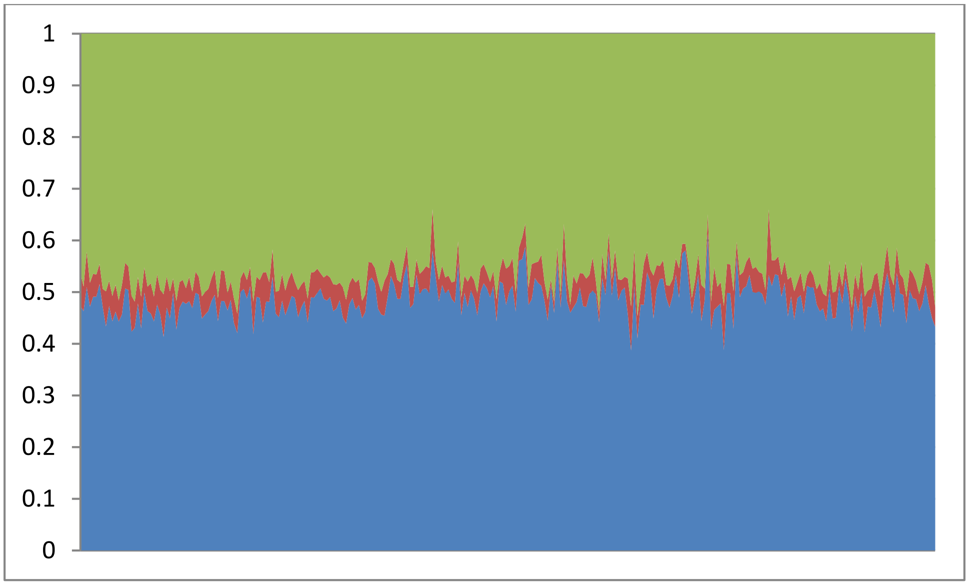

Chromosome 5:

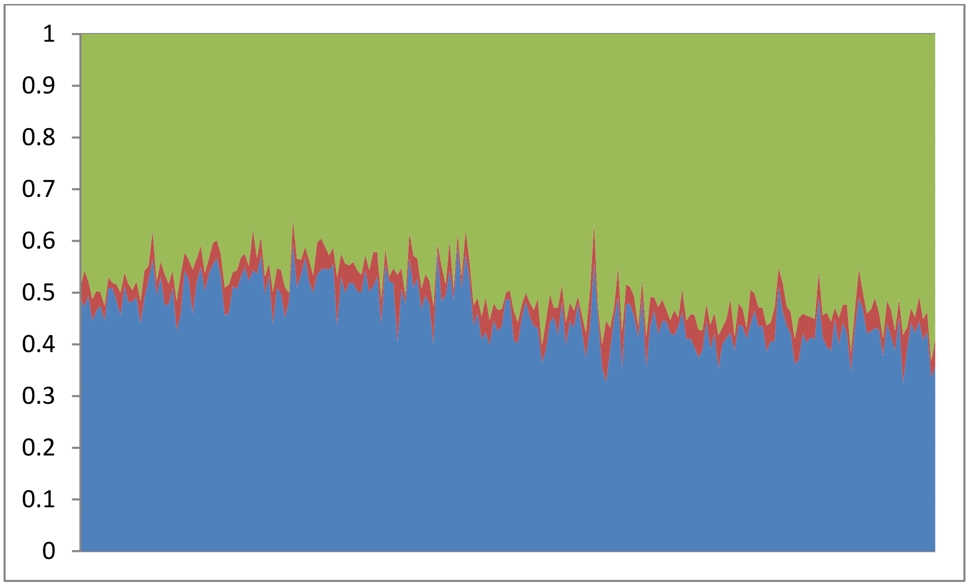

Chromosome 6:

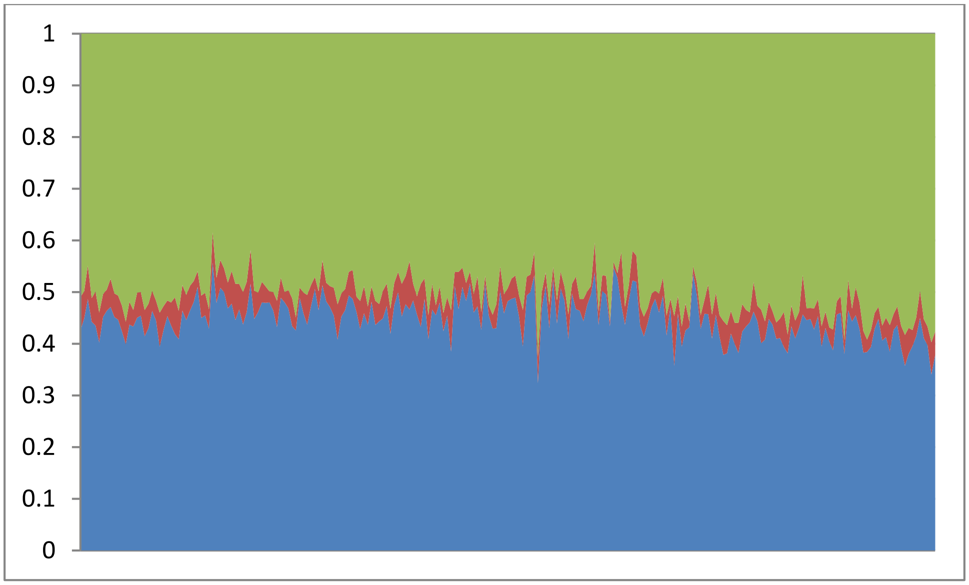

Chromosome 7:

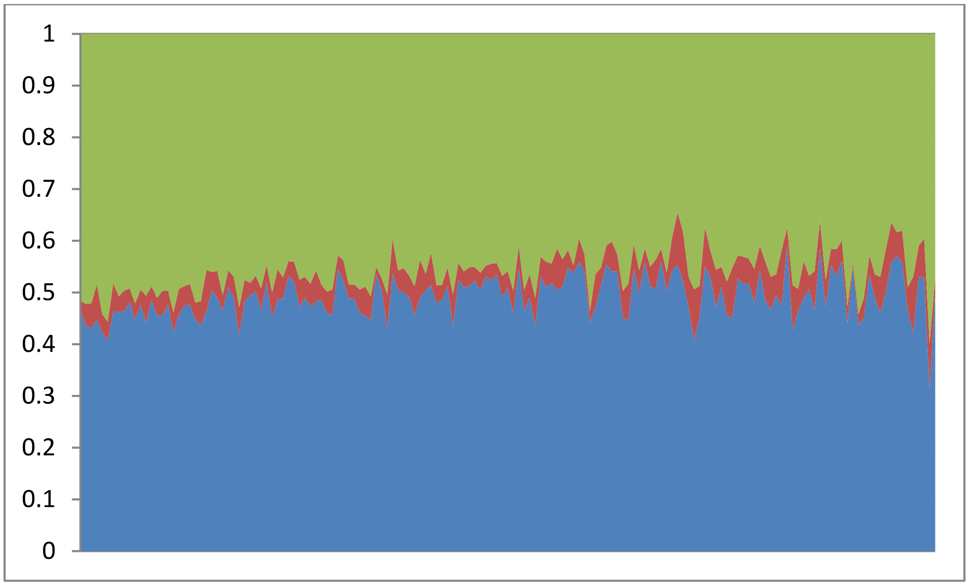

Chromosome 8:

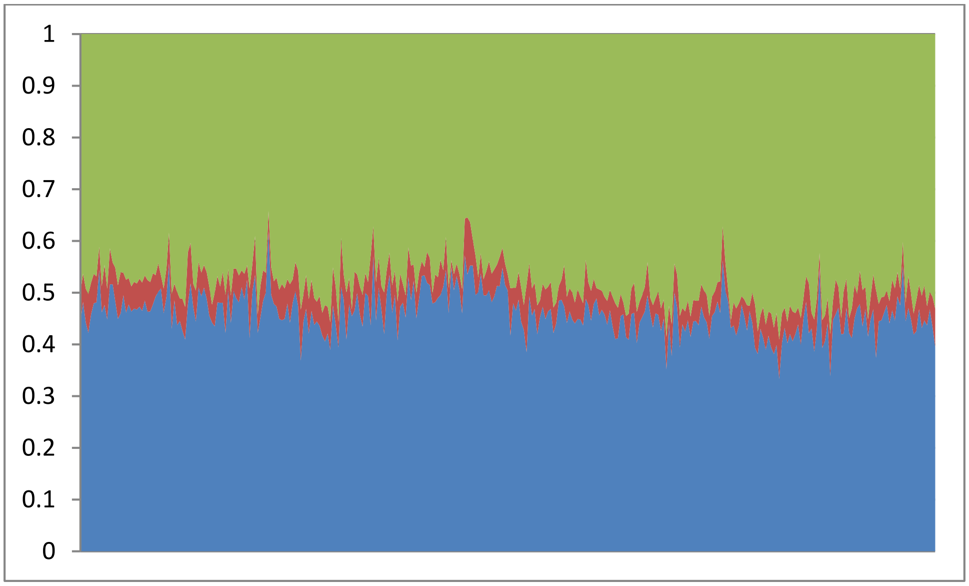

Chromosome 9:

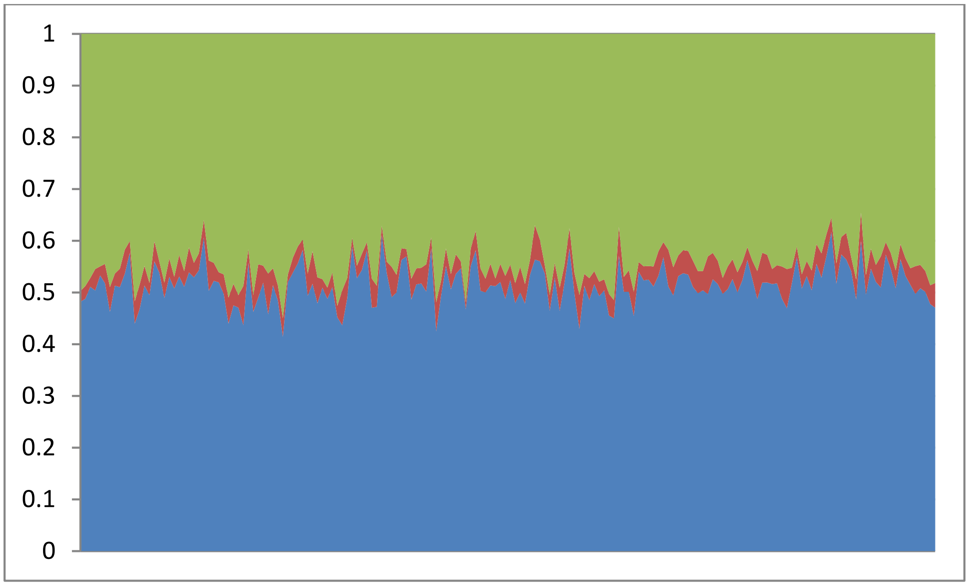

Chromosome 10:

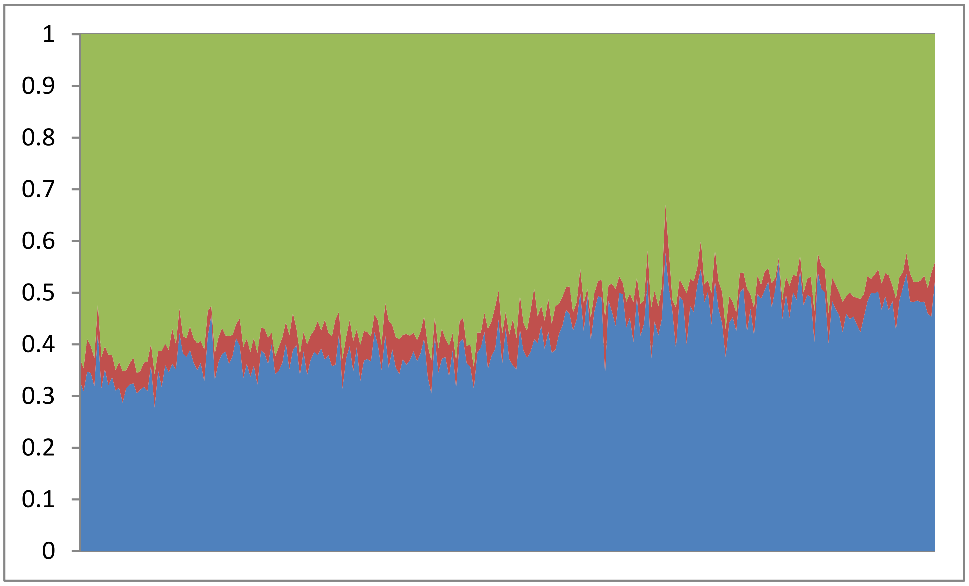

Chromosome 11:

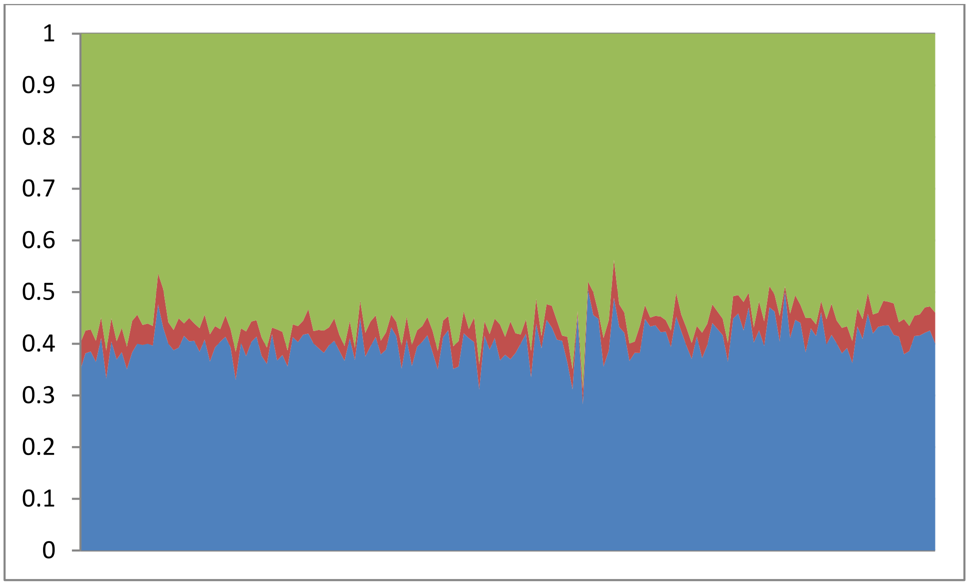

Chromosome 12:

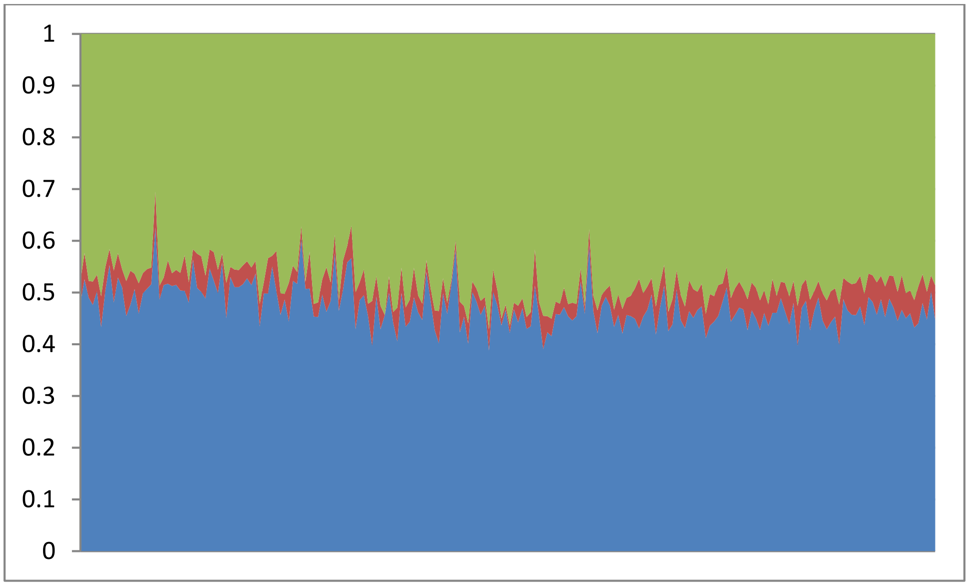

Chromosome 13:

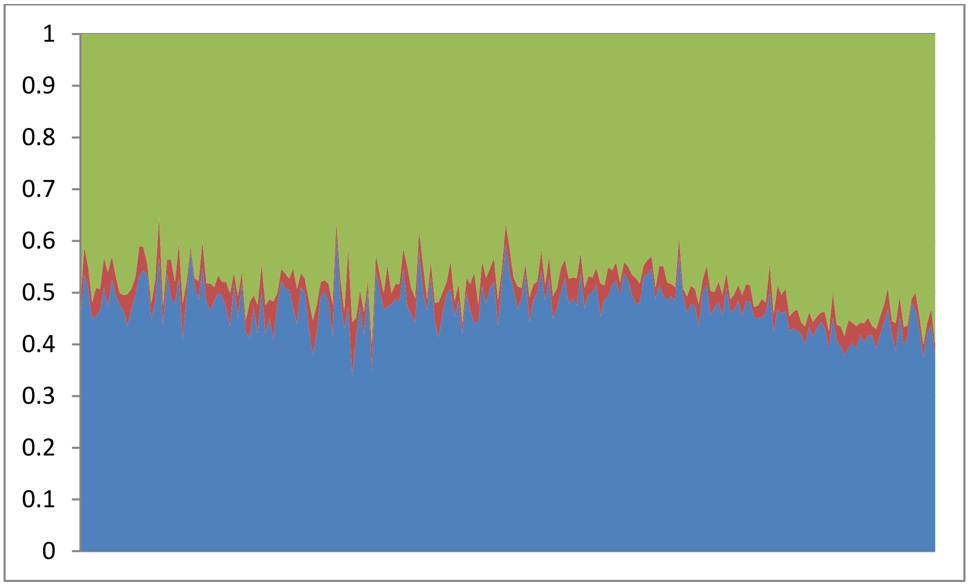

Chromosome 14:

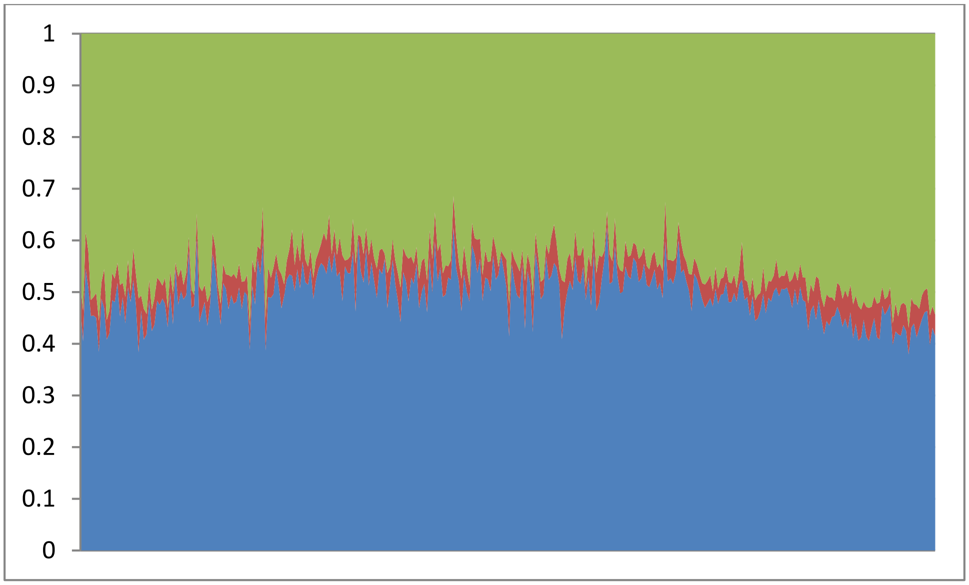

Figure S3 A graphical depiction of RIL Genotype proportions across the 14 chromosomes. Here blue = IM/IM, red = IM/PR (H), and green = PR/PR.

Table S1 Full genetic map and the genotype calls for 480 RILs. The markers are listed in row 1, the chromosome for each marker in row 2, and the map position within chromosome in row 3. RIL identity is given in column A. Genotypes at each marker are given in subsequent columns. Here, A = homozygous for IM767, B = homozygous for PR, H = heterozygote, and - indicates no call.

Available for download as an Excel file at http://www.g3journal.org/lookup/suppl/doi:10.1534/g3.113.010124/-/DC1

Table S2 Results of GLM ANOVA. Factors with a significant effect on levels of a particular PPG are in bold type.

PPG	Factor	F	df numerator, df denominator	p-value
Conandroside	**RIL (Grow-up)**	6.69	207, 571	<0.001
	Damage?	3.51	1, 571	0.061
	**Grow-up**	10.23	2, 571	<0.001
	**Quant. method**	8.25	1, 571	0.004
PPG 5	**RIL (Grow-up)**	2.45	207, 571	<0.001
	Damage?	0.10	1, 571	0.756
	**Grow-up**	3.17	2, 571	0.044
	Quant. method	0.22	1, 571	0.641
PPG 7	**RIL (Grow-up)**	5.32	149, 193	<0.001
	Damage?	0.01	1, 193	0.944
	**Grow-up**	8.02	2, 193	<0.001
	Quant. method	---	---	---
