# Supplementary material for: A High-Resolution Genetic Map of Yellow Monkeyflower Identifies Chemical Defense QTLs and Recombination Rate Variation
Source: G3 (Bethesda). 2014 Mar 13;4(5):813–21. doi: 10.1534/g3.113.010124 (PMC4025480; doi:10.1534/g3.113.010124)
Supplement: Supporting Information [file supp_g3.113.010124_FigureS1.pdf]

Chromosome 14

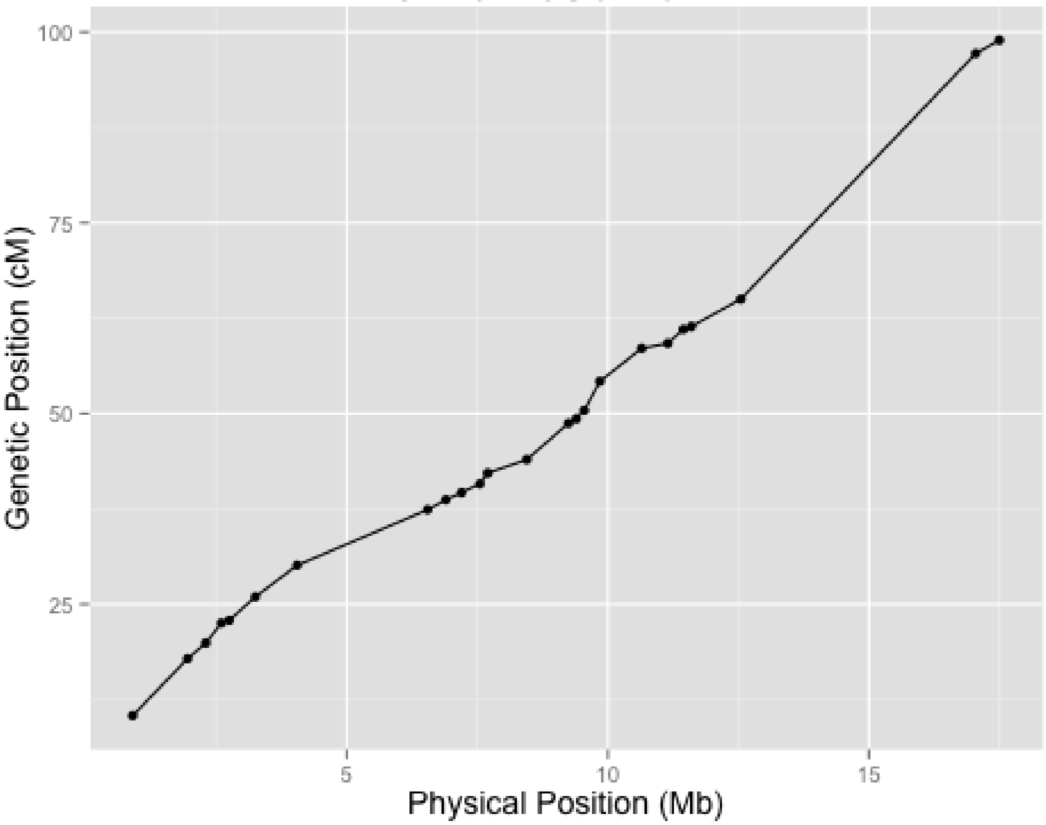

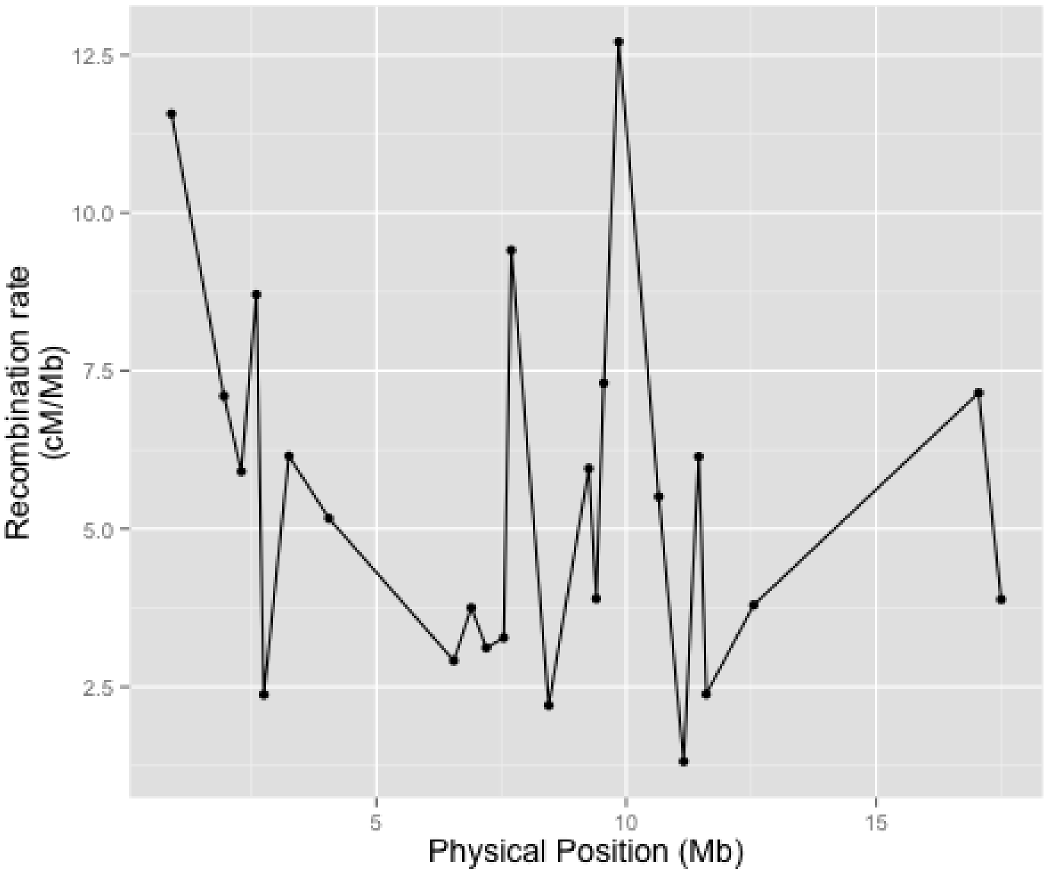

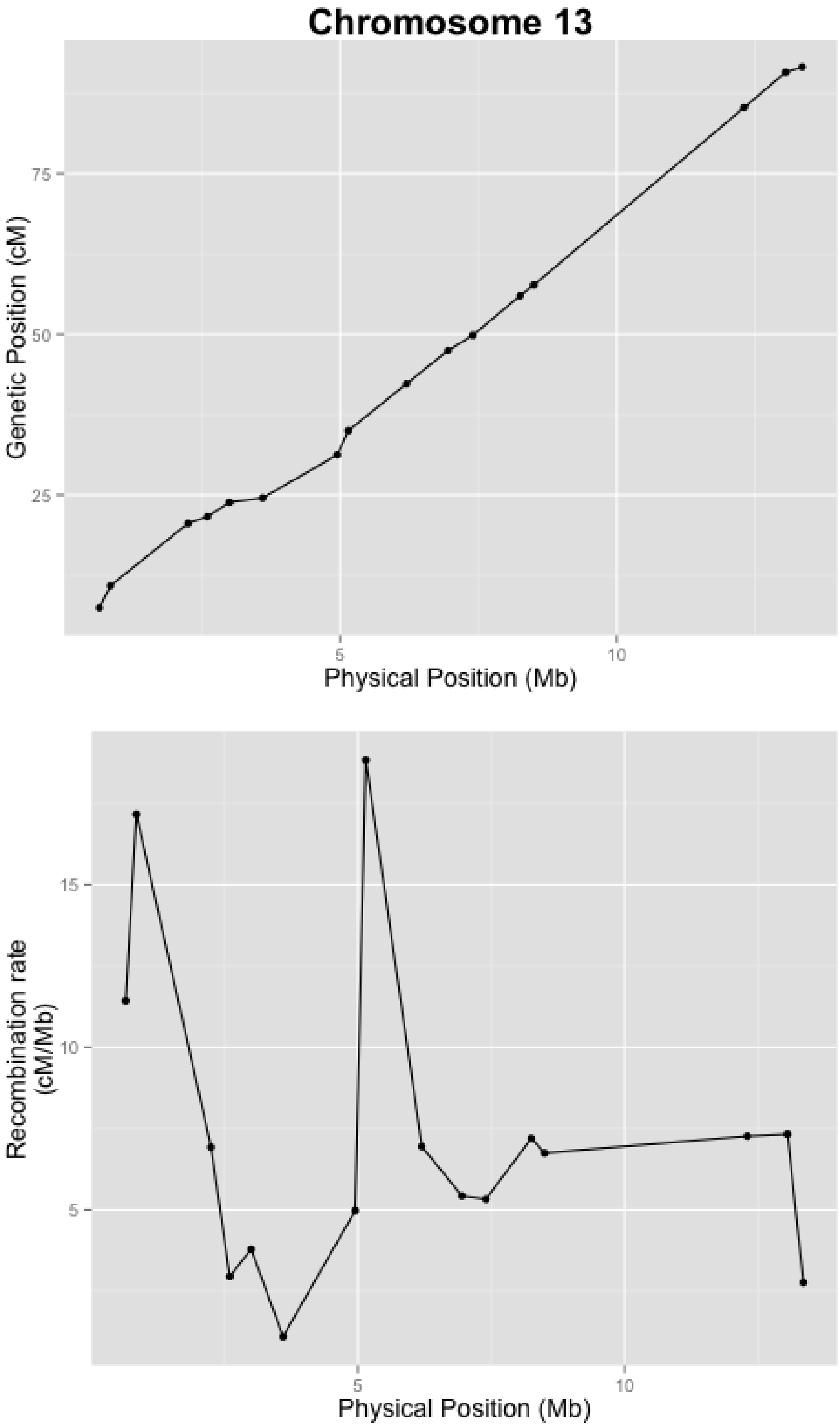
Chromosome 13
Genetic Position (cM)
75
50
25
5
10
Physical Position (Mb)
Recombination rate
(cM/Mb)
15
10
5
5
10
Physical Position (Mb)

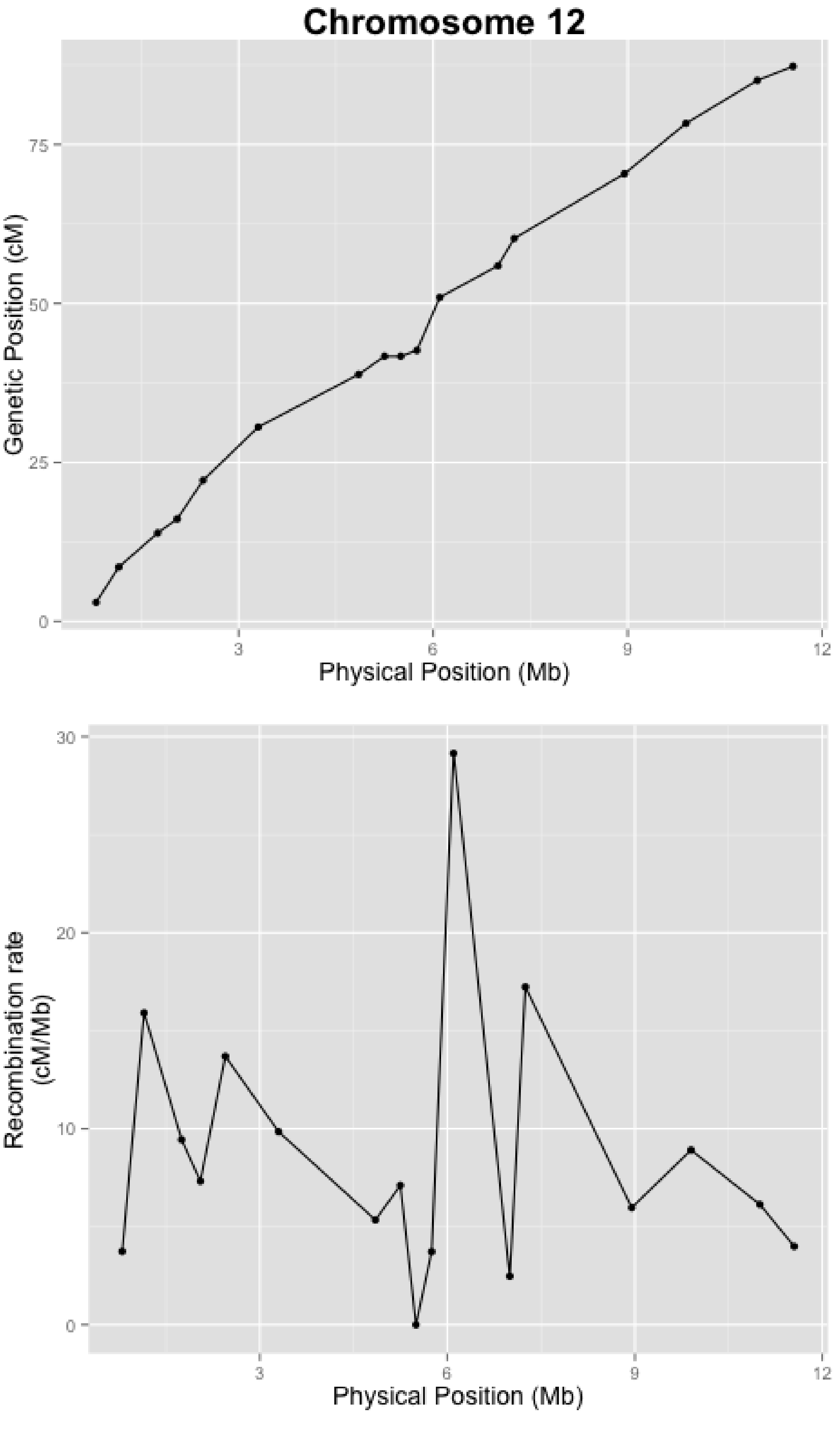

Chromosome 12
Genetic Position (cM)
0
25
50
75
3
6
9
12
Physical Position (Mb)
Recombination rate
(cM/Mb)
0
10
20
30
3
6
9
12
Physical Position (Mb)

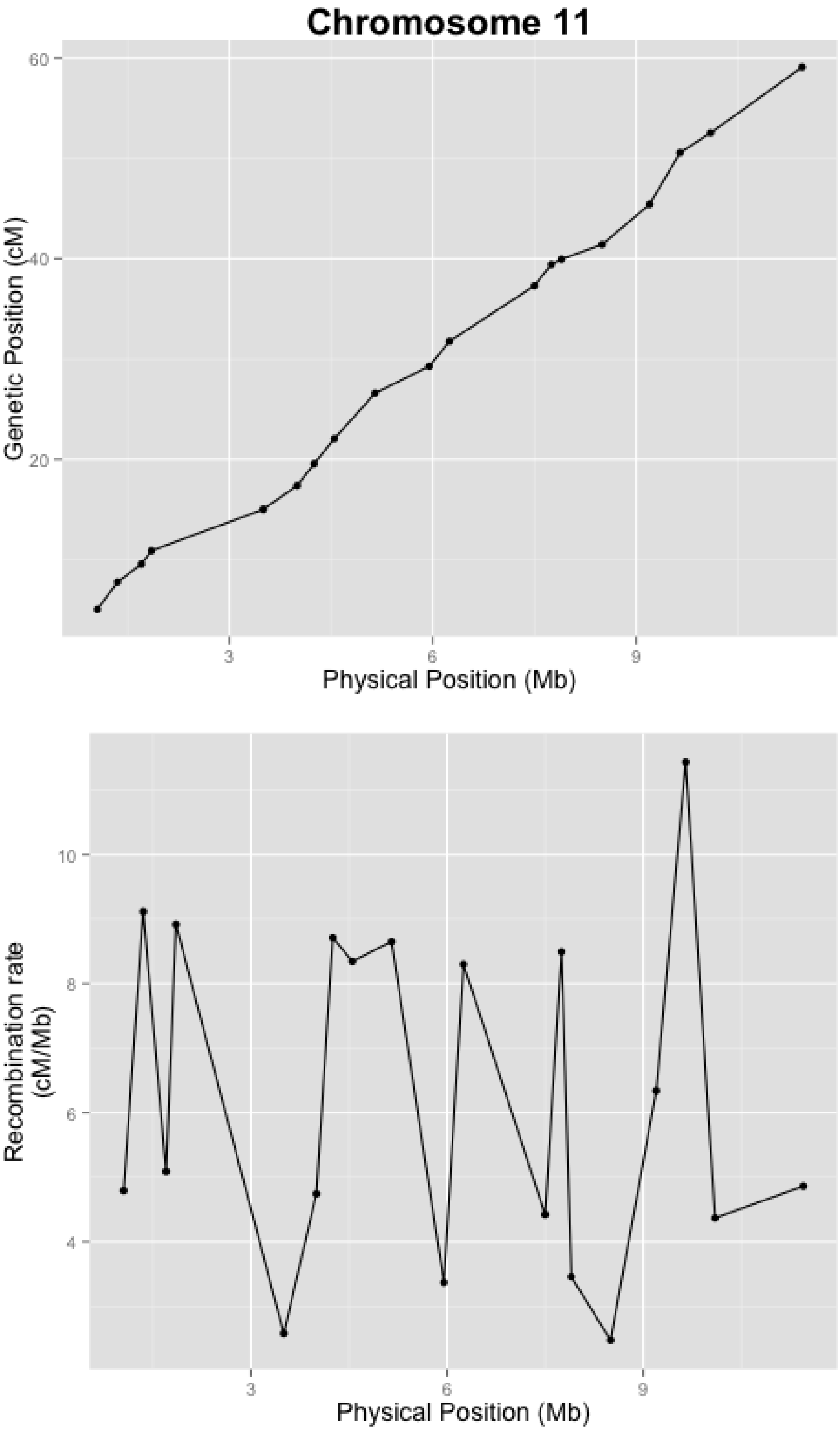

Chromosome 11
60
40
20
Genetic Position (cM)
3
6
9
Physical Position (Mb)
10
8
6
4
Recombination rate
(cM/Mb)
3
6
9
Physical Position (Mb)

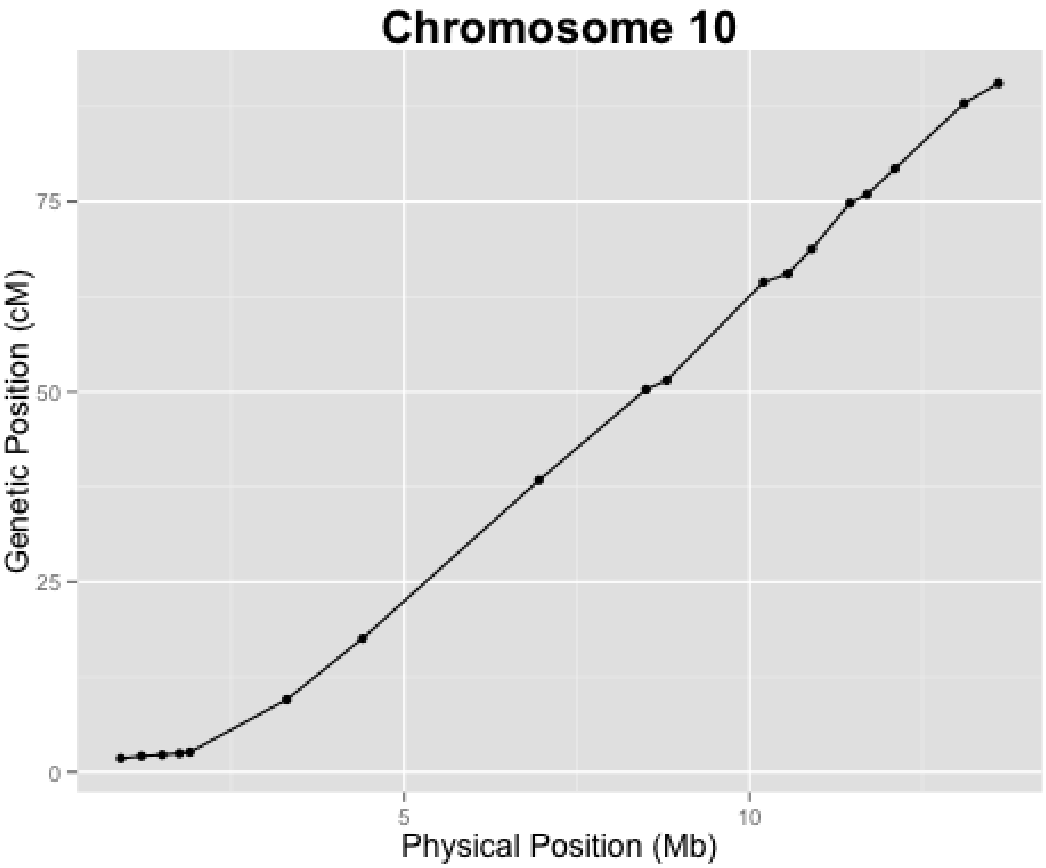
Chromosome 10
Genetic Position (cM)
75
50
25
0
5
10
Physical Position (Mb)

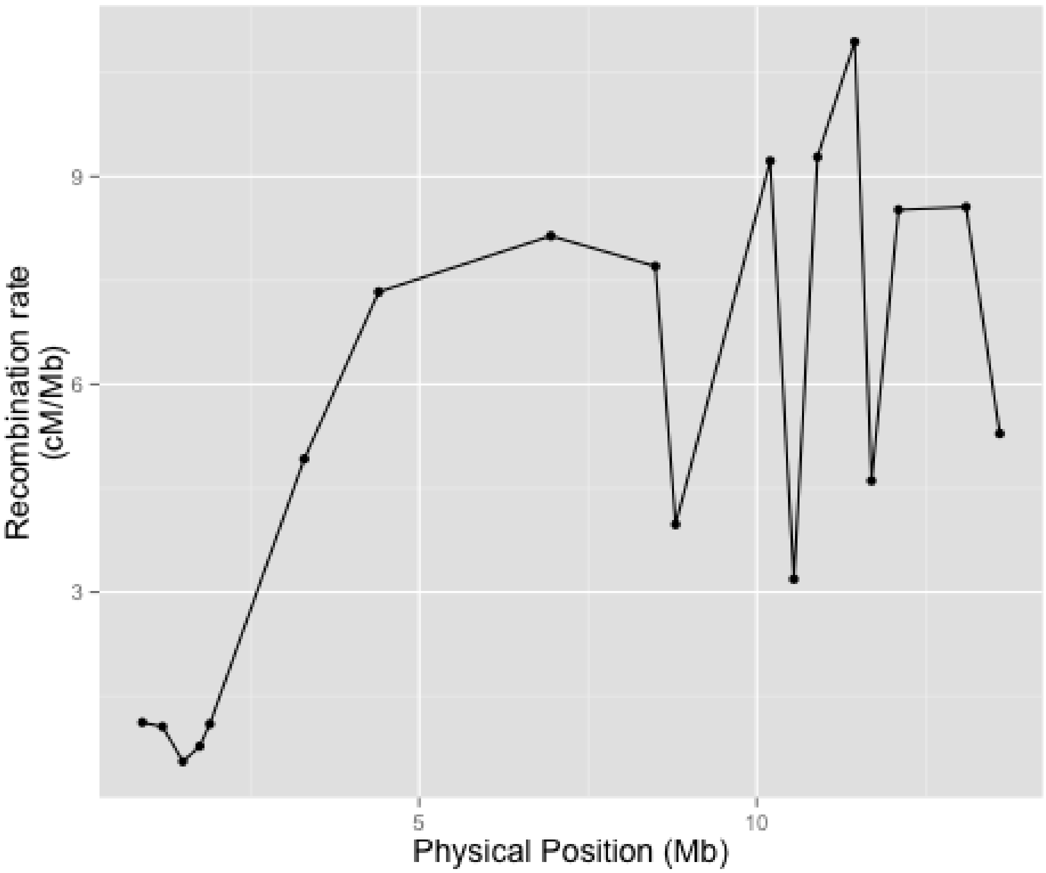
Recombination rate (cM/Mb)
9
6
3
5
10
Physical Position (Mb)

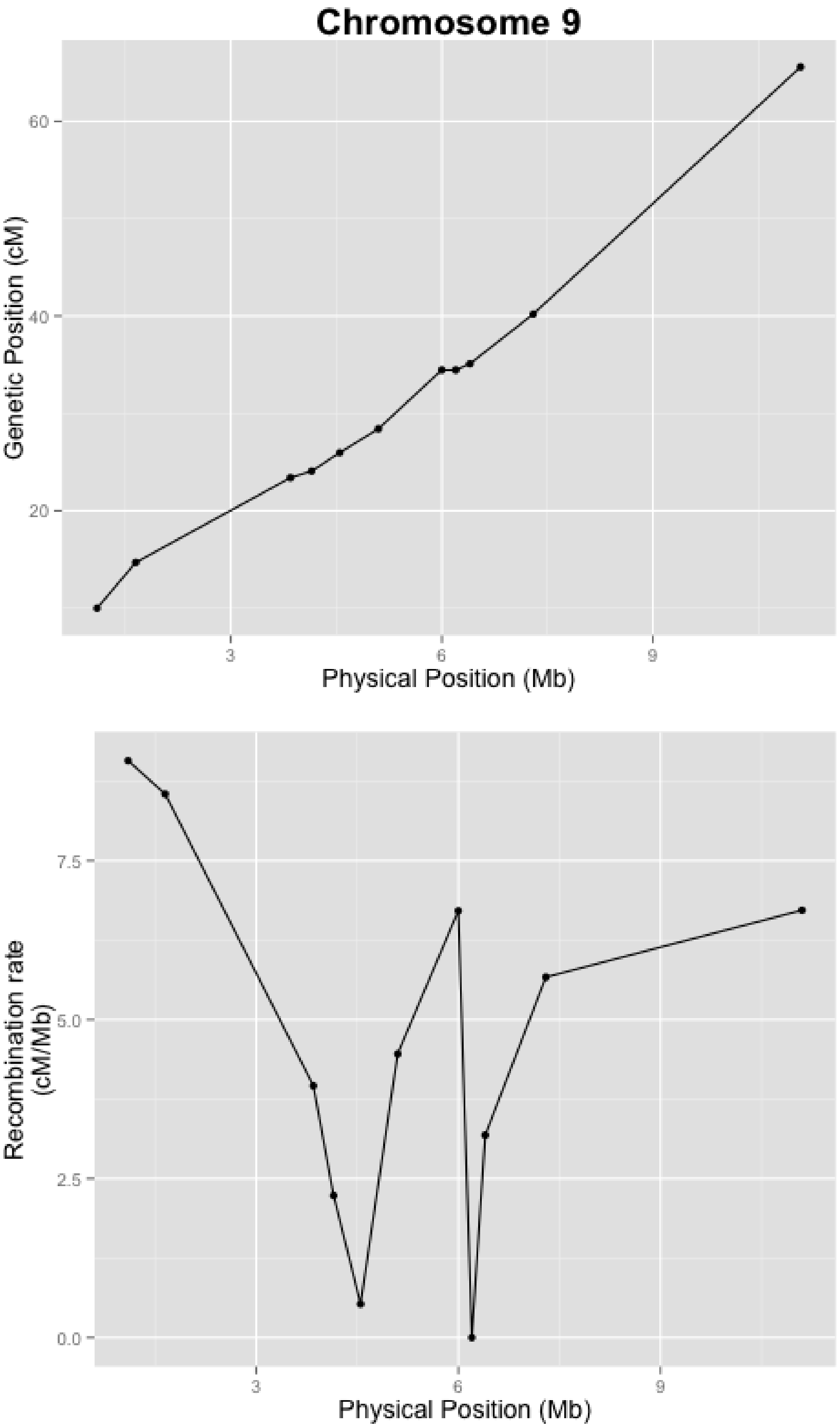
Chromosome 9
Genetic Position (cM)
60
40
20
3
6
9
Physical Position (Mb)
Recombination rate (cM/Mb)
7.5
5.0
2.5
0.0
3
6
9
Physical Position (Mb)

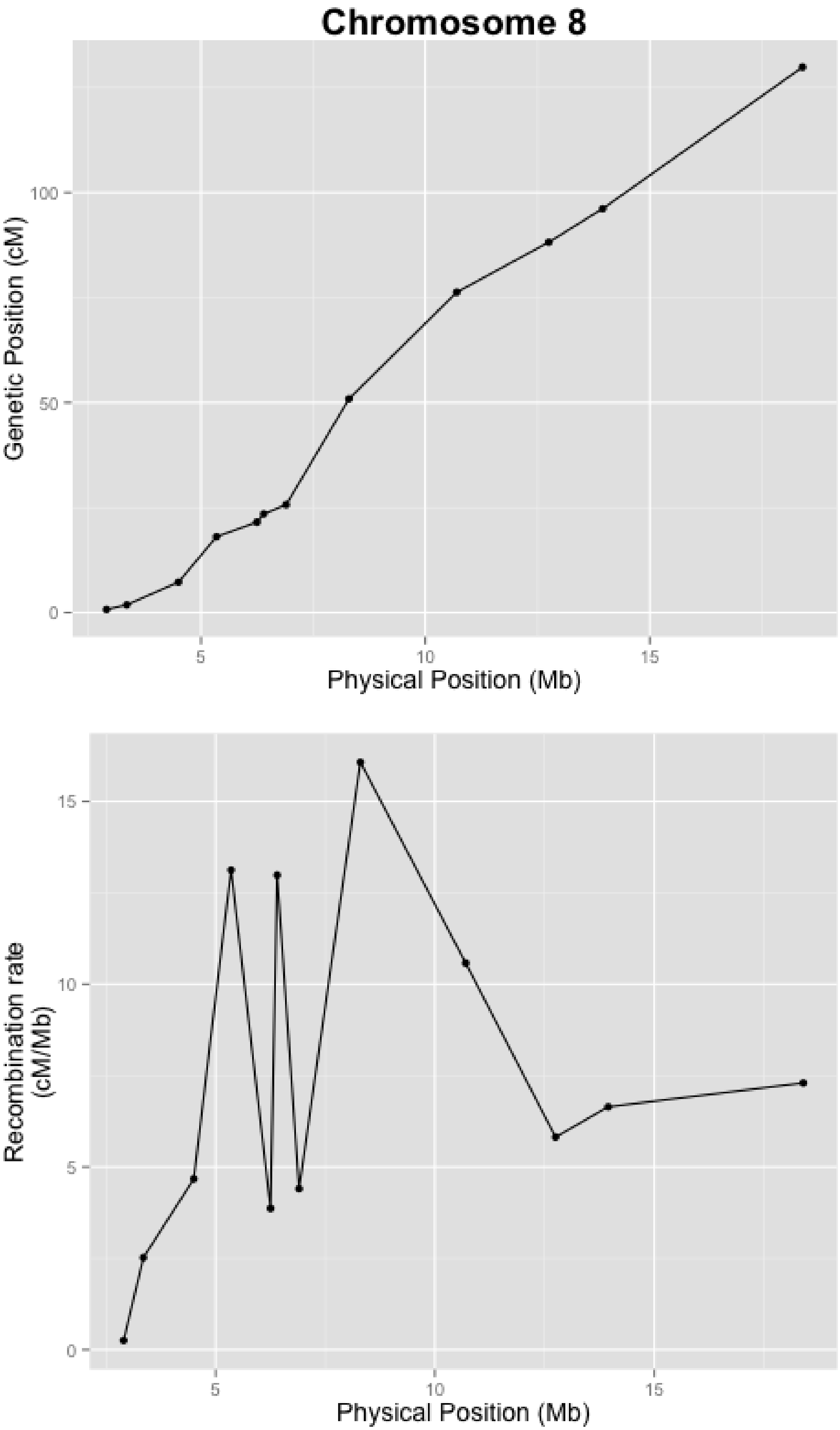
Chromosome 8
Genetic Position (cM)
0
50
100
5
10
15
Physical Position (Mb)
Recombination rate (cM/Mb)
0
5
10
15
5
10
15
Physical Position (Mb)

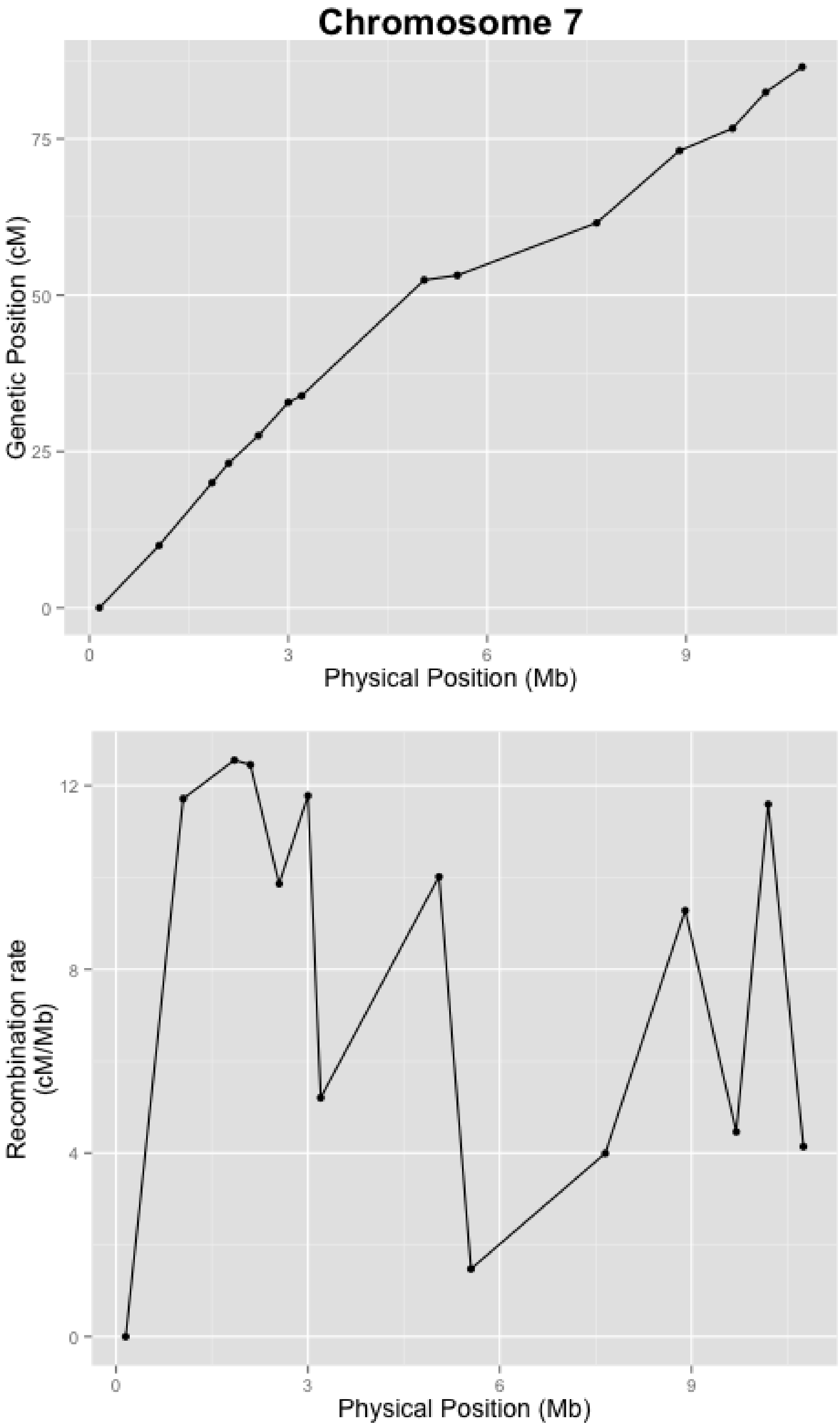
Chromosome 7
Genetic Position (cM)
0
25
50
75
0
3
6
9
Physical Position (Mb)
Recombination rate
(cM/Mb)
0
4
8
12
0
3
6
9
Physical Position (Mb)

Chromosome 6

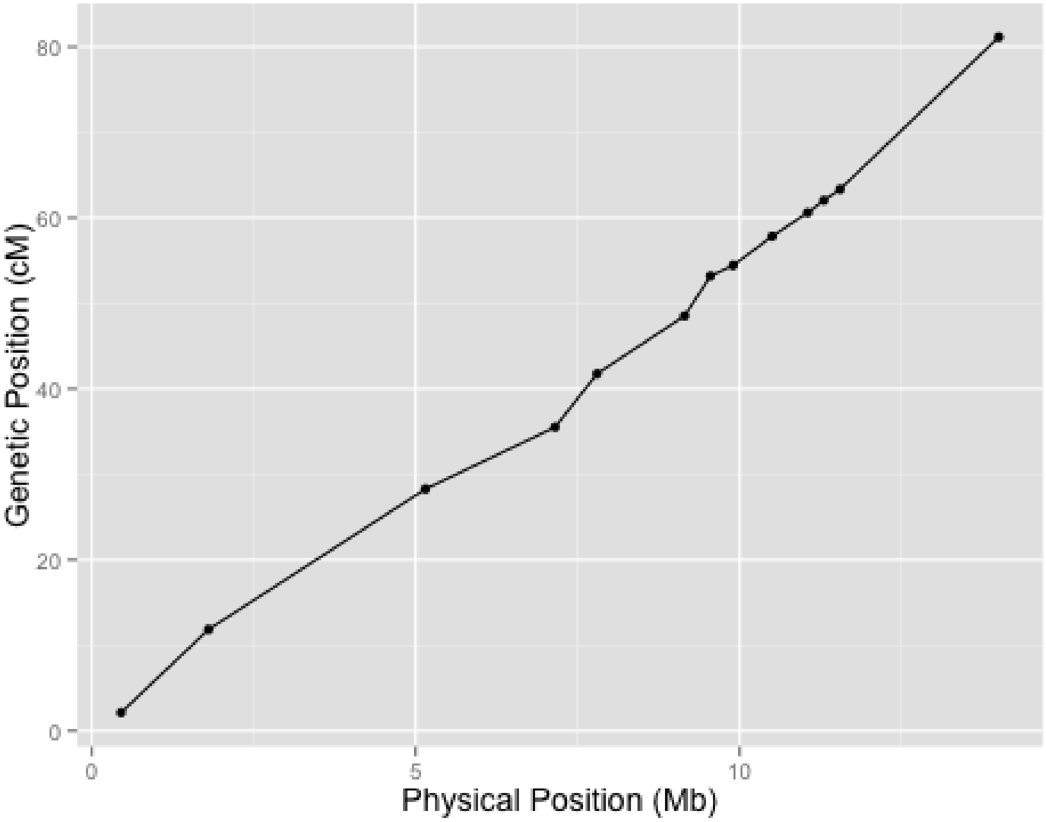

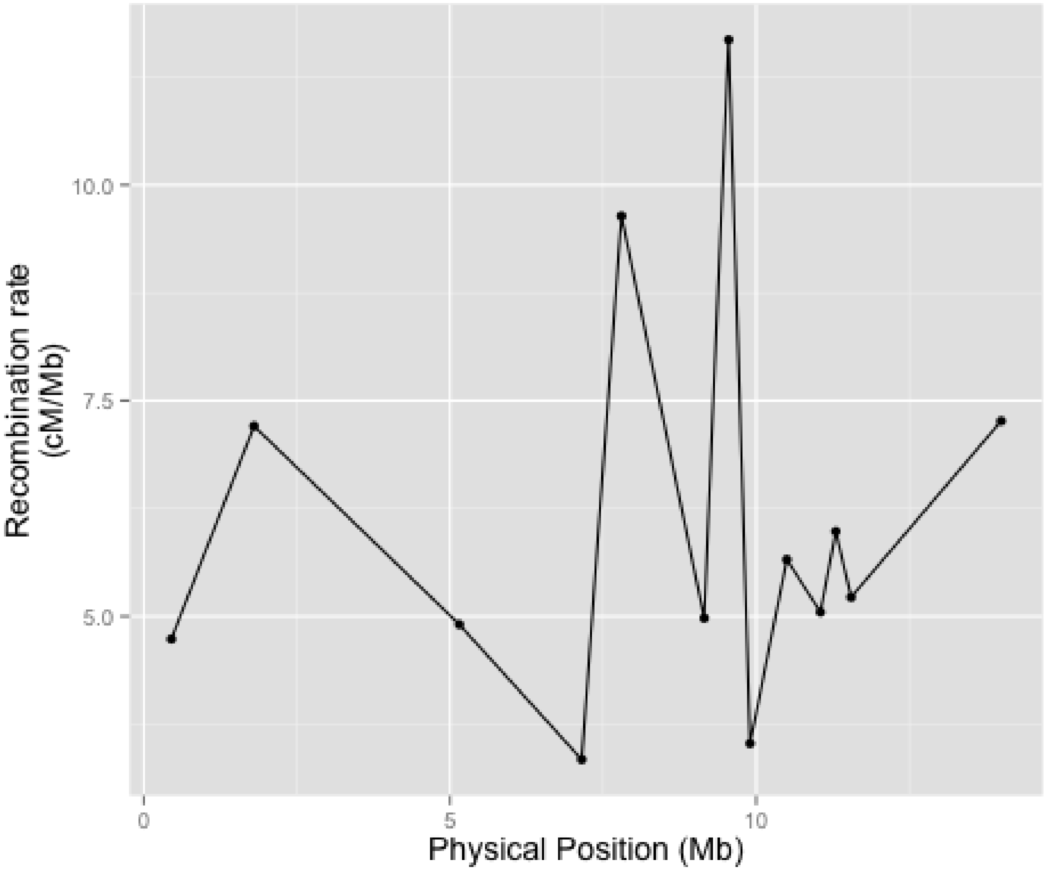

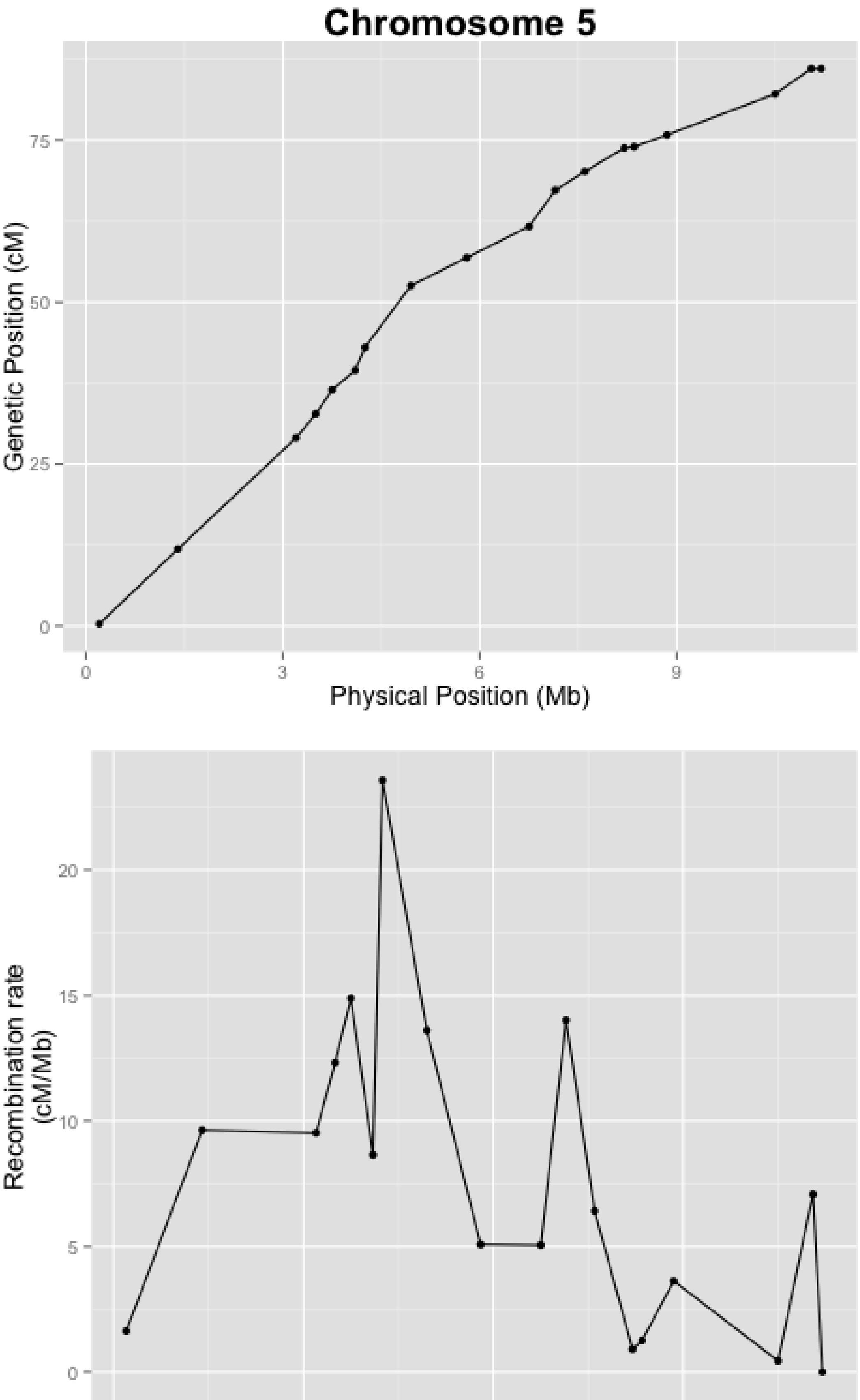
Chromosome 5
Genetic Position (cM)
0
25
50
75
Physical Position (Mb)
0
3
6
9
Recombination rate
(cM/Mb)
0
5
10
15
20
Physical Position (Mb)
0
3
6
9

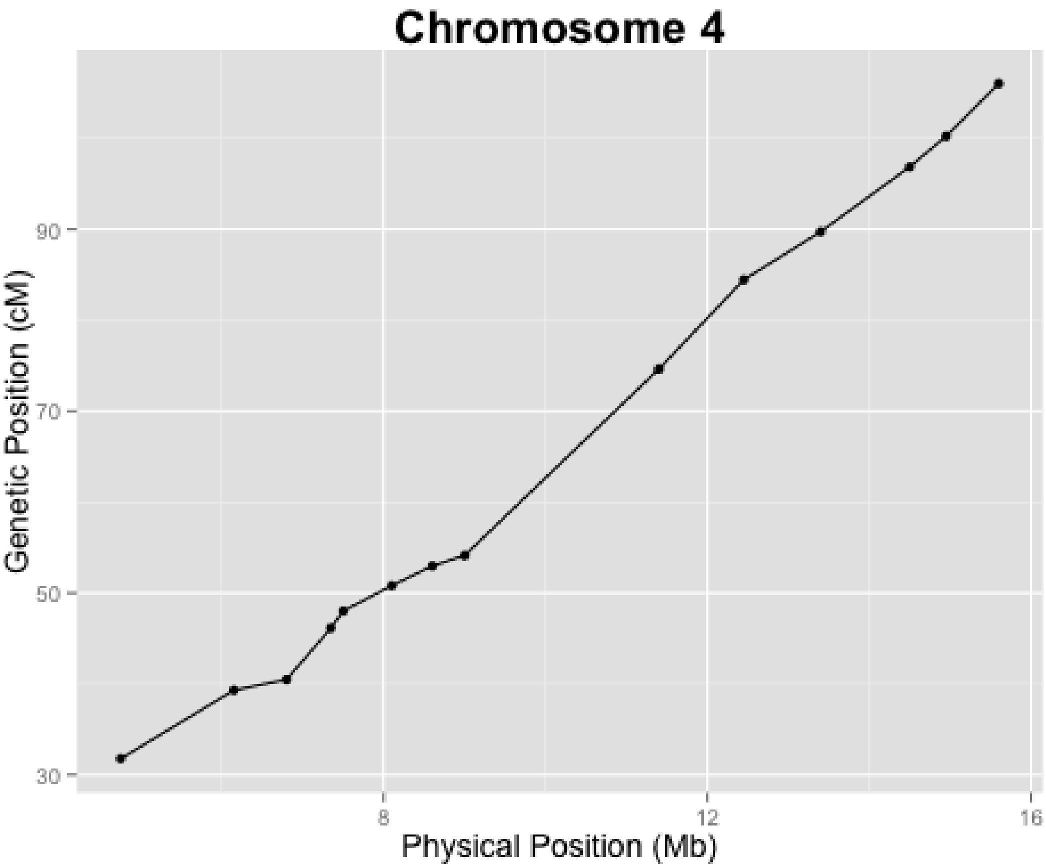
Chromosome 4
Genetic Position (cM)
90
70
50
30
8
12
16
Physical Position (Mb)

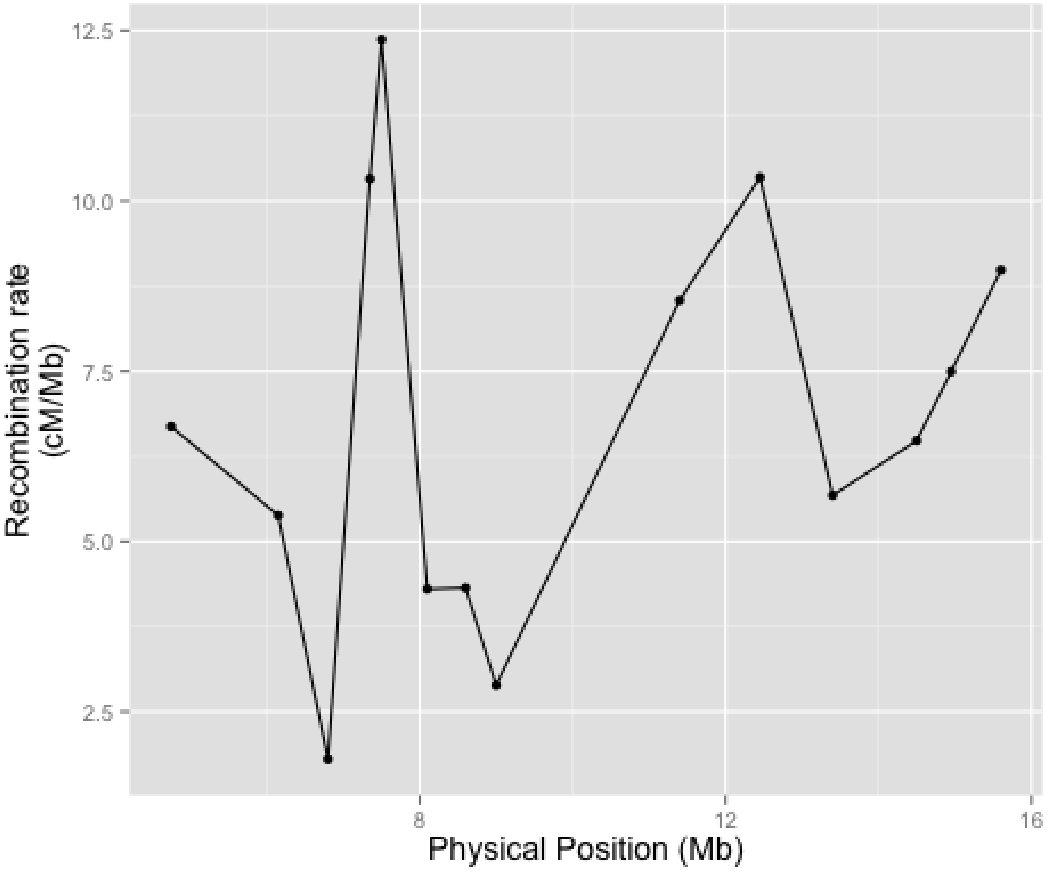
Recombination rate (cM/Mb)
12.5
10.0
7.5
5.0
2.5
8
12
16
Physical Position (Mb)

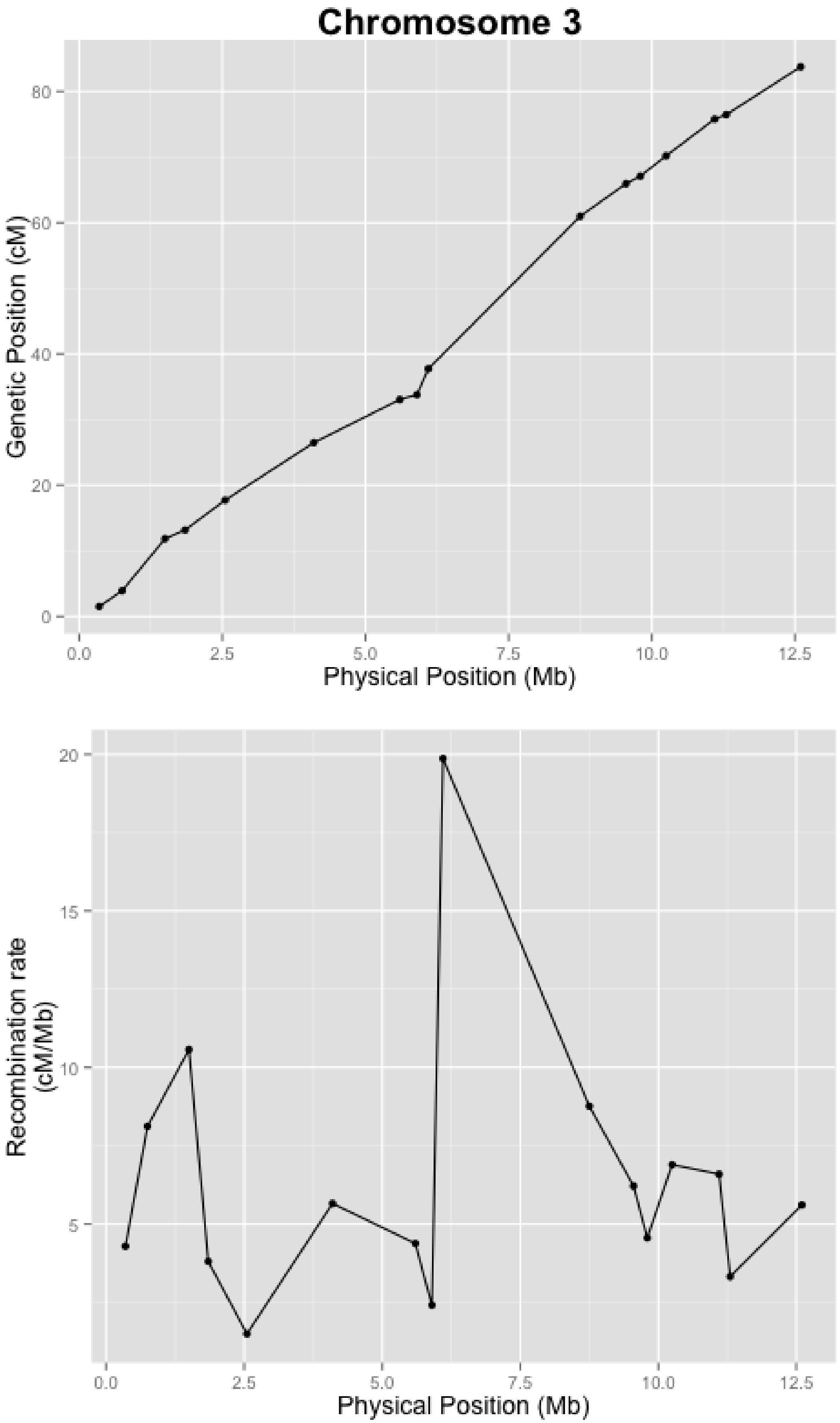
Chromosome 3
Genetic Position (cM)
0
20
40
60
80
0.0
2.5
5.0
7.5
10.0
12.5
Physical Position (Mb)
Recombination rate
(cM/Mb)
5
10
15
20
0.0
2.5
5.0
7.5
10.0
12.5
Physical Position (Mb)

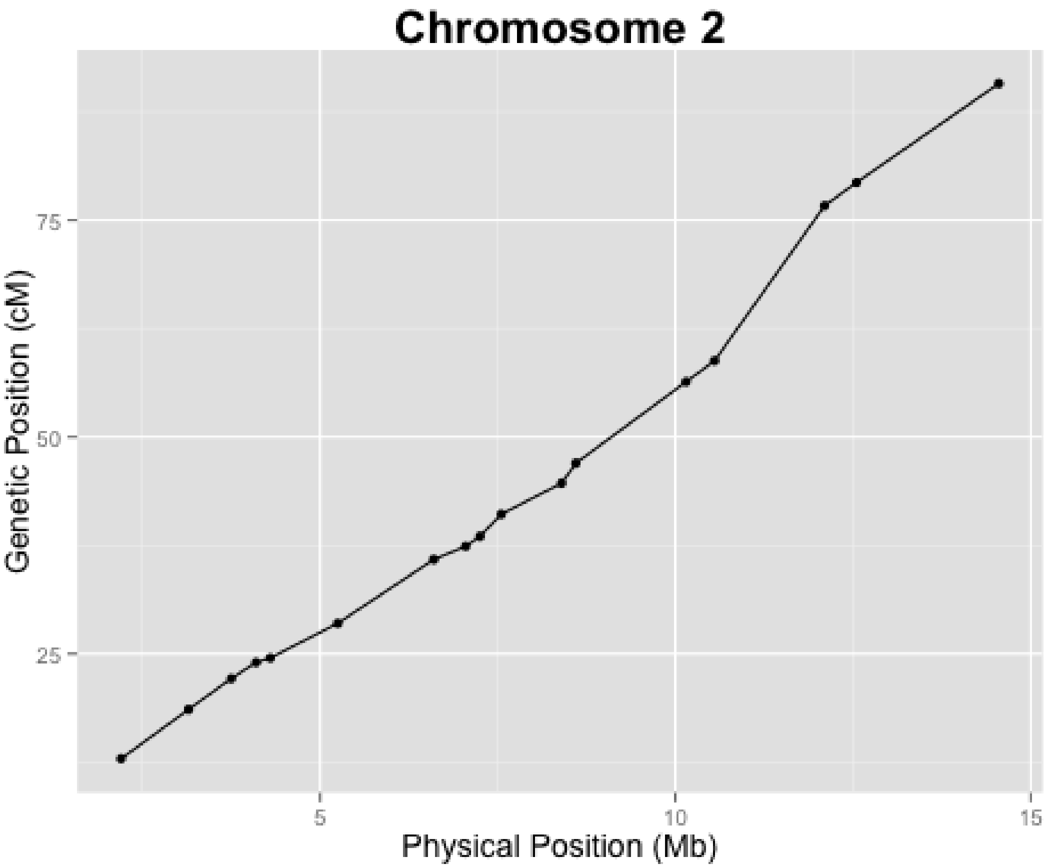
Chromosome 2
Genetic Position (cM)
75
50
25
5
10
15
Physical Position (Mb)

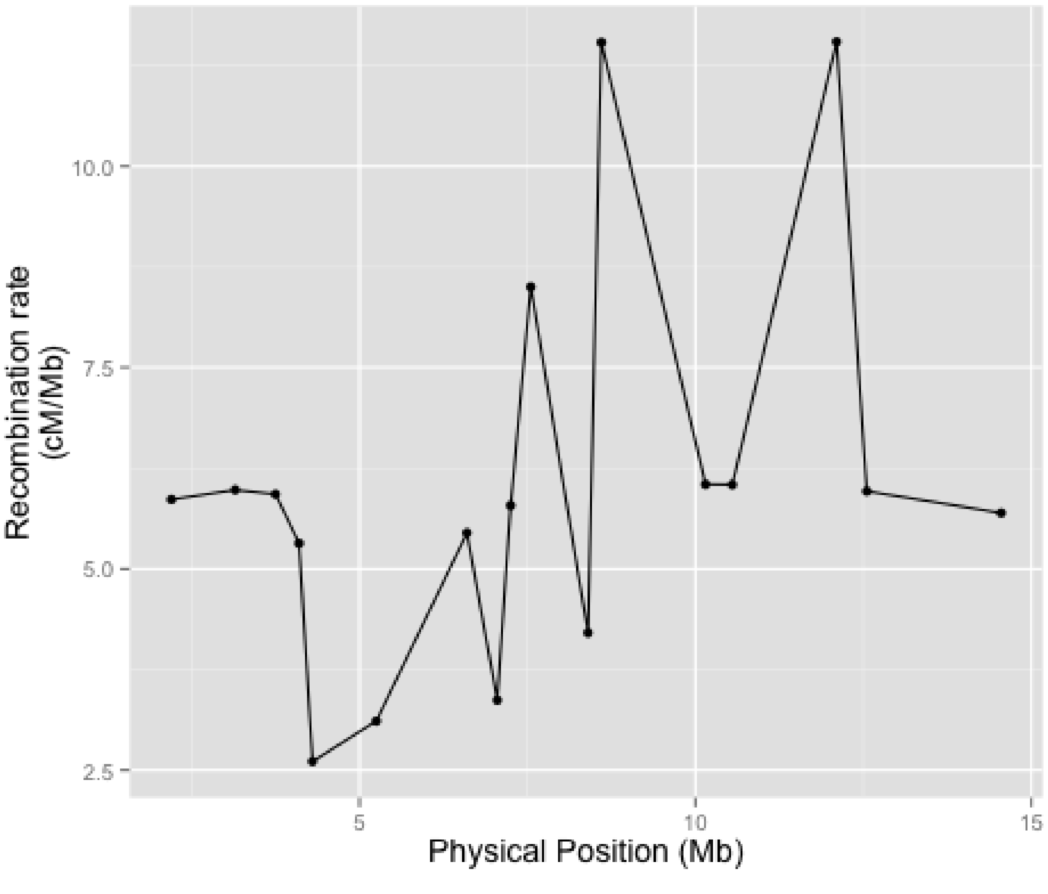
Recombination rate
(cM/Mb)
10.0
7.5
5.0
2.5
5
10
15
Physical Position (Mb)

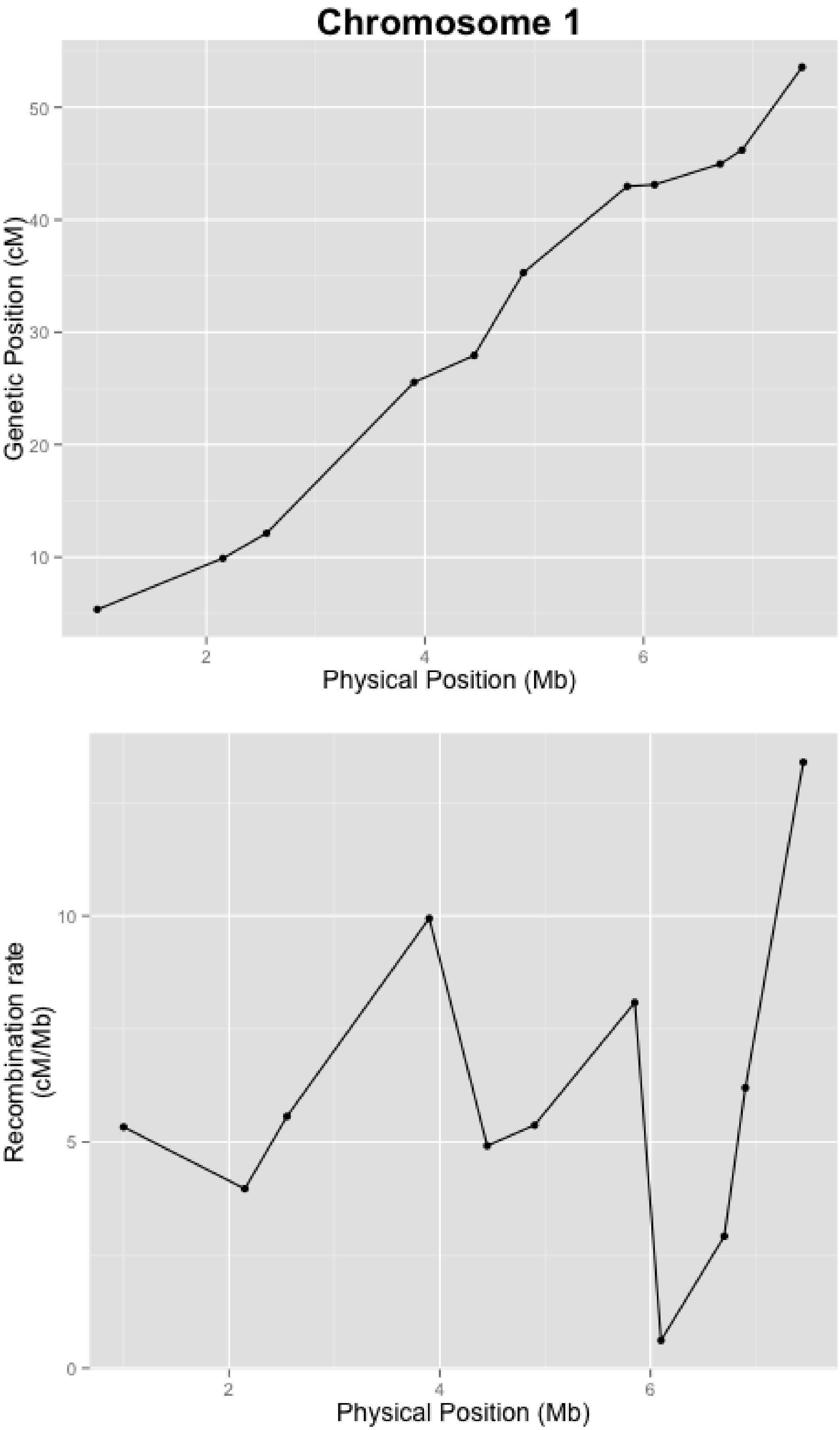
Chromosome 1
Genetic Position (cM)
50
40
30
20
10
2
4
6
Physical Position (Mb)
Recombination rate
(cM/Mb)
10
5
0
2
4
6
Physical Position (Mb)

Figure S1 Depiction of recombination rate variation along each chromosome. Each point is an estimate from an entire scaffold. Scaffolds were aligned according to their order in the linkage map. Note that the apparently adjacent scaffolds are not physically adjacent as we do not account for distance between scaffolds. The distance between points corresponds to the length of the scaffolds. Only scaffolds >100 kb are depicted, and extreme observations were removed for better visualization.
