# Supplementary material for: A High-Resolution Genetic Map of Yellow Monkeyflower Identifies Chemical Defense QTLs and Recombination Rate Variation
Source: G3 (Bethesda). 2014 Mar 13;4(5):813–21. doi: 10.1534/g3.113.010124 (PMC4025480; doi:10.1534/g3.113.010124)
Supplement: Supporting Information [file supp_g3.113.010124_FigureS2.pdf]

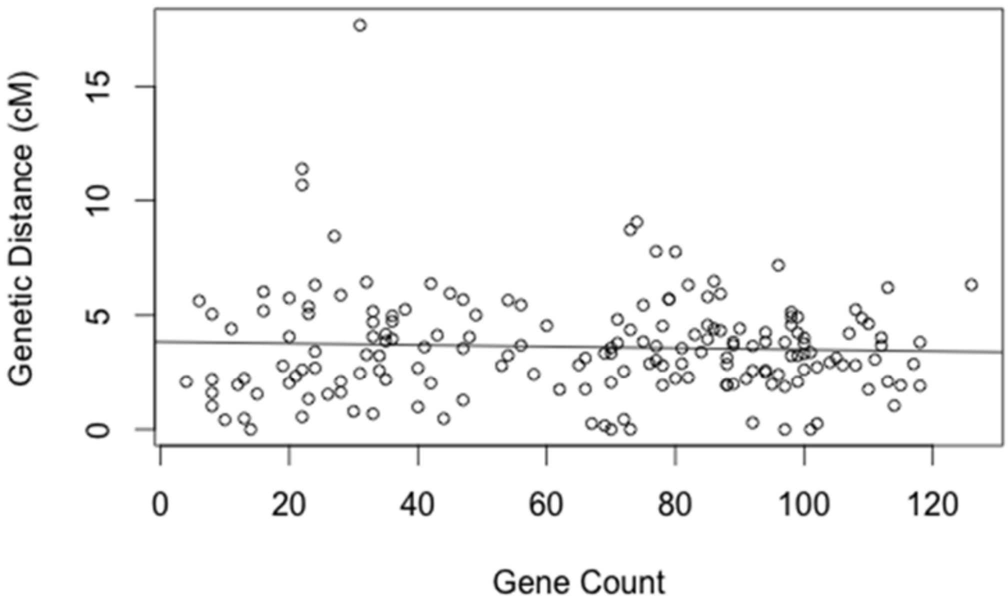

Figure S2 The number of genes plotted against the genetic distance for windows of ~500 kb. Only scaffolds >500 kb were included in the figure. The solid line is a least squares regression line indicating little evidence of a relationship.
