# Supplementary material for: A High-Resolution Genetic Map of Yellow Monkeyflower Identifies Chemical Defense QTLs and Recombination Rate Variation
Source: G3 (Bethesda). 2014 Mar 13;4(5):813–21. doi: 10.1534/g3.113.010124 (PMC4025480; doi:10.1534/g3.113.010124)
Supplement: Supporting Information [file supp_g3.113.010124_FigureS3.pdf]

Chromosome 1:

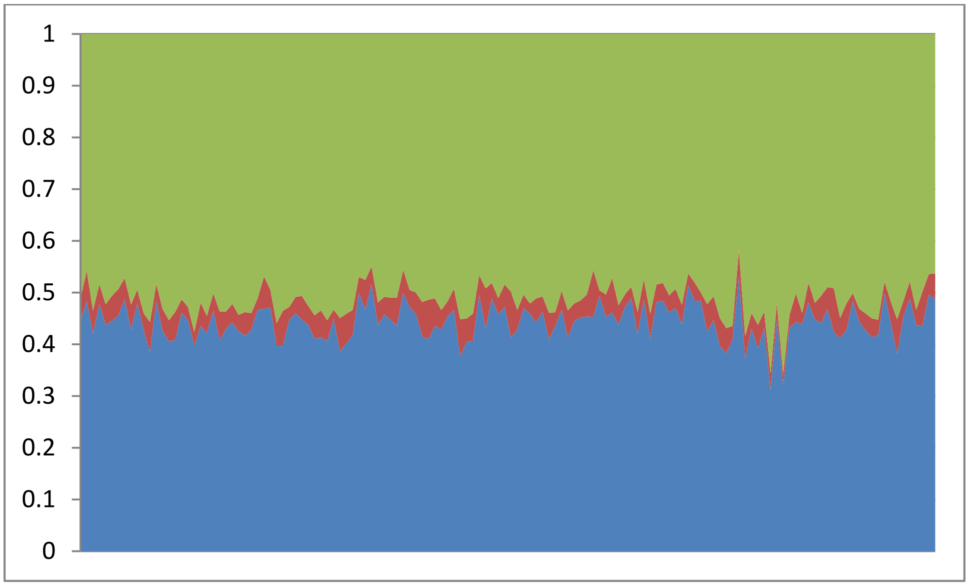

Chromosome 2:

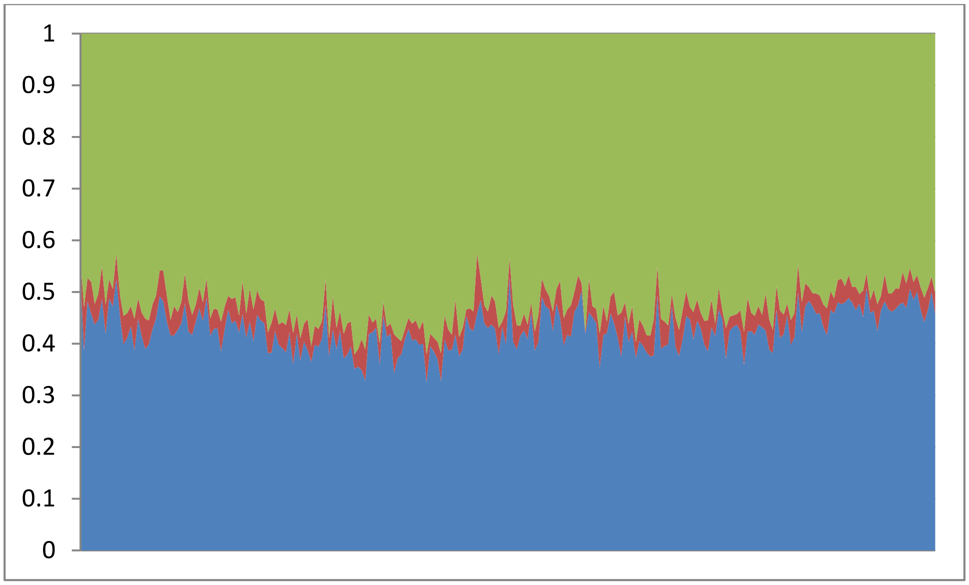

Chromosome3:

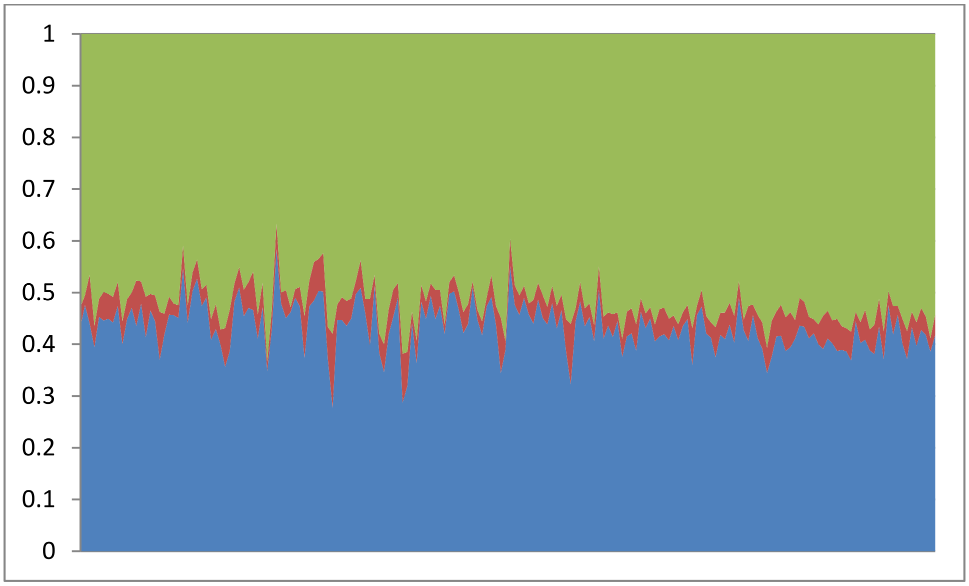

Chromosome 4:

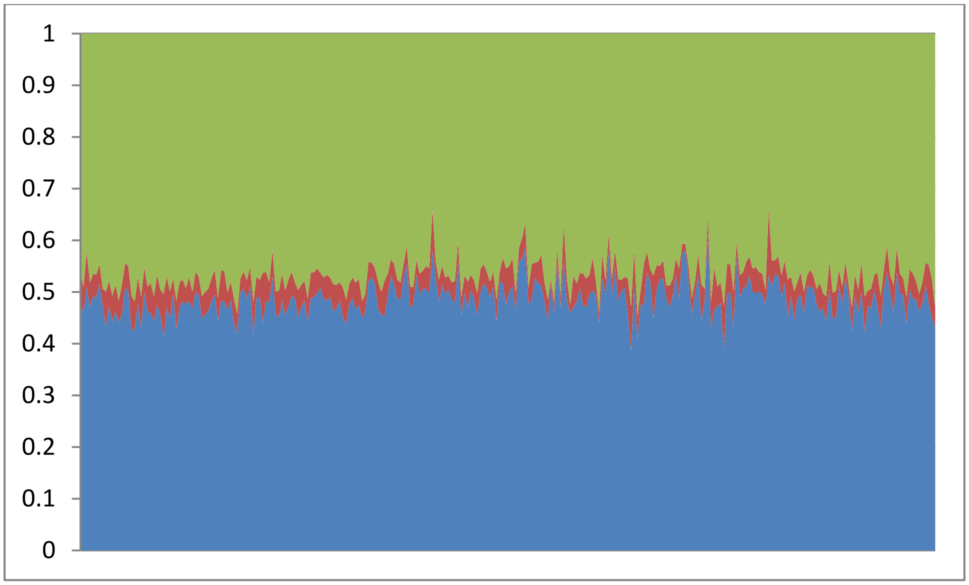

Chromosome 5:

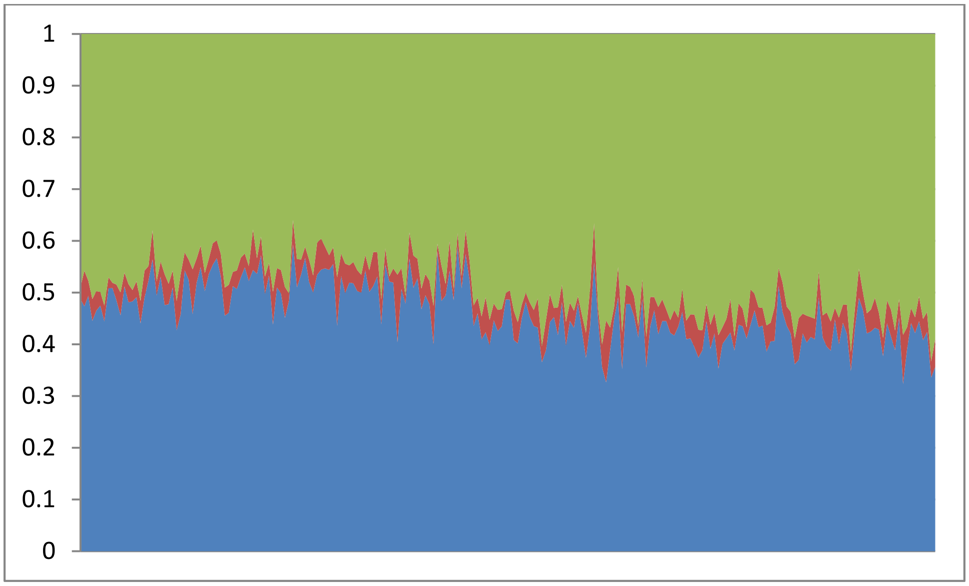

Chromosome 6:

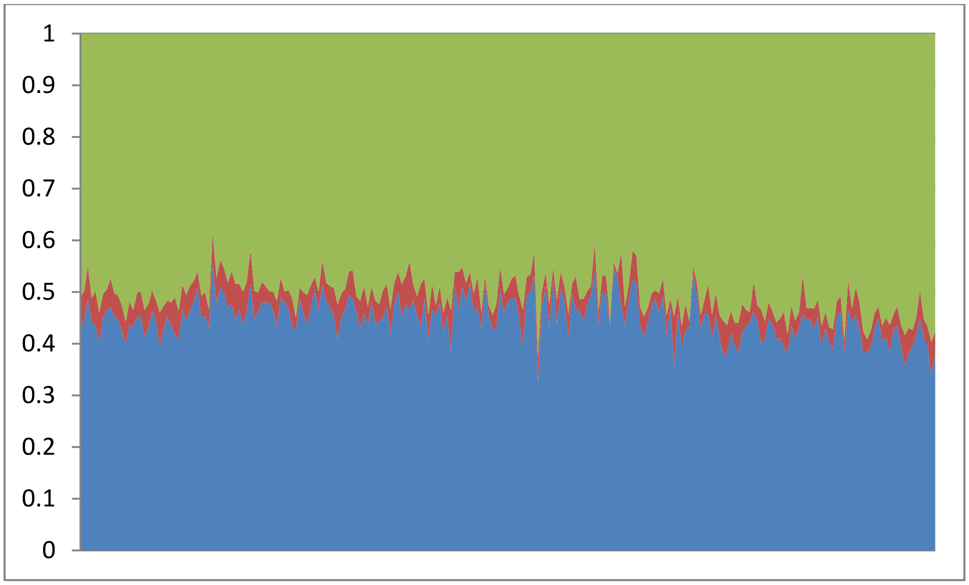

Chromosome 7:

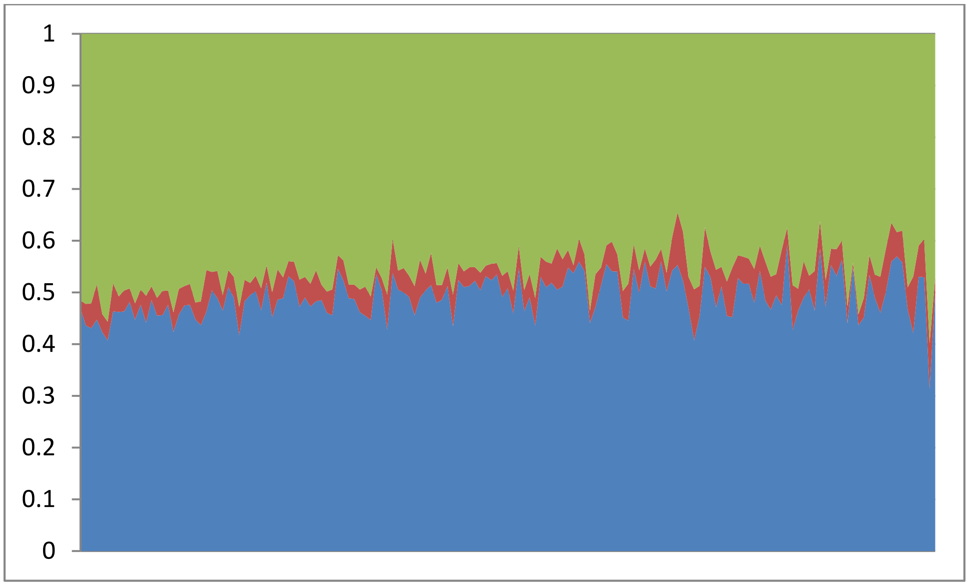

Chromosome 8:

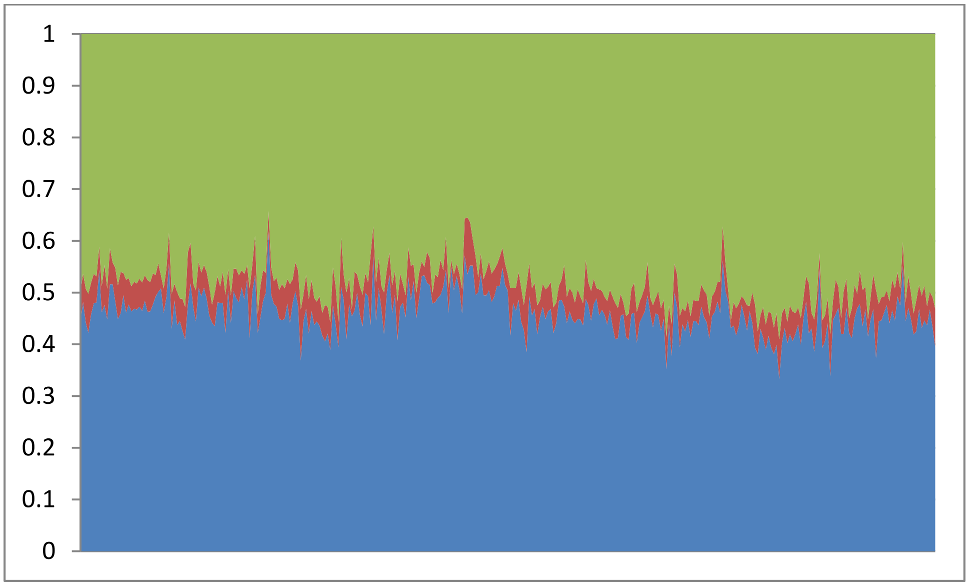

Chromosome 9:

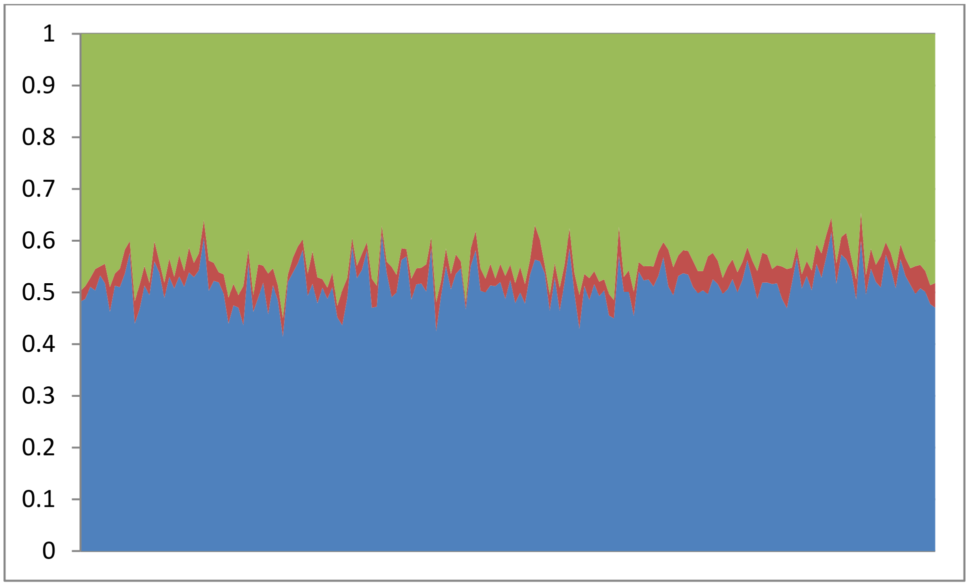

Chromosome 10:

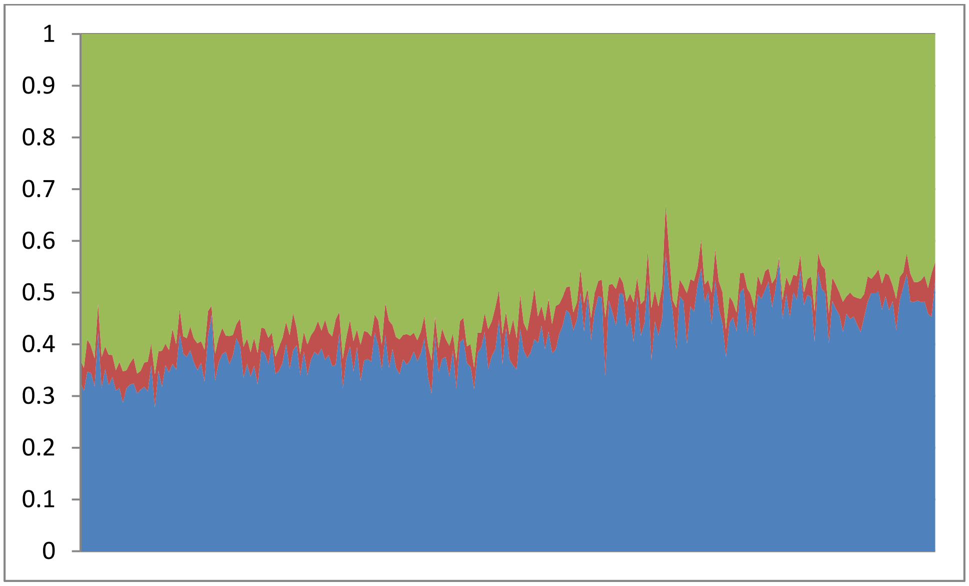

Chromosome 11:

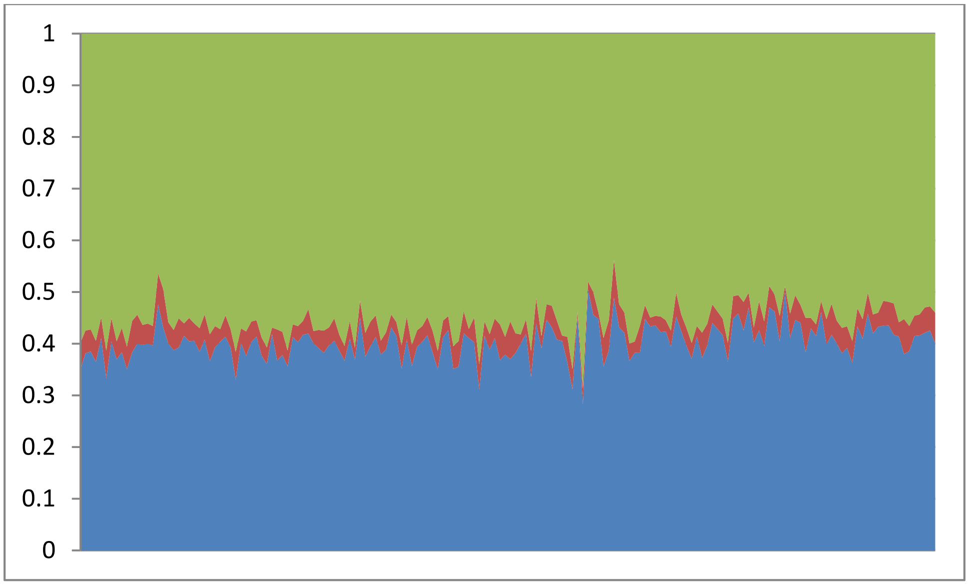

Chromosome 12:

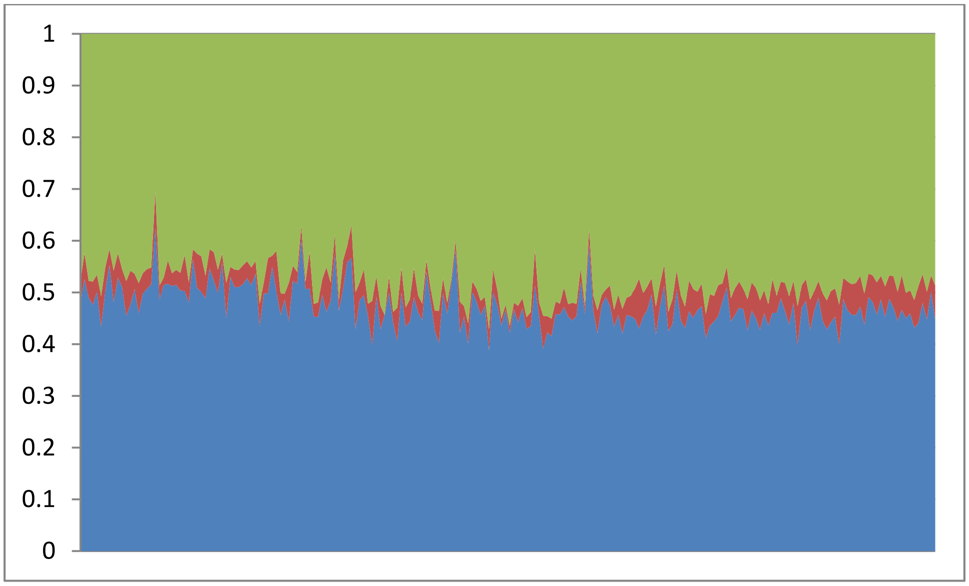

Chromosome 13:

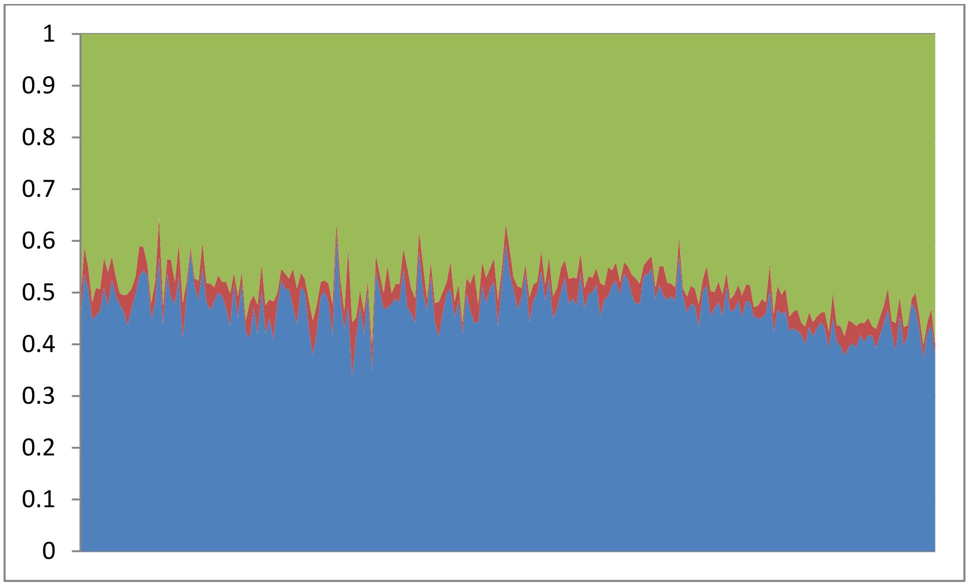

Chromosome 14:

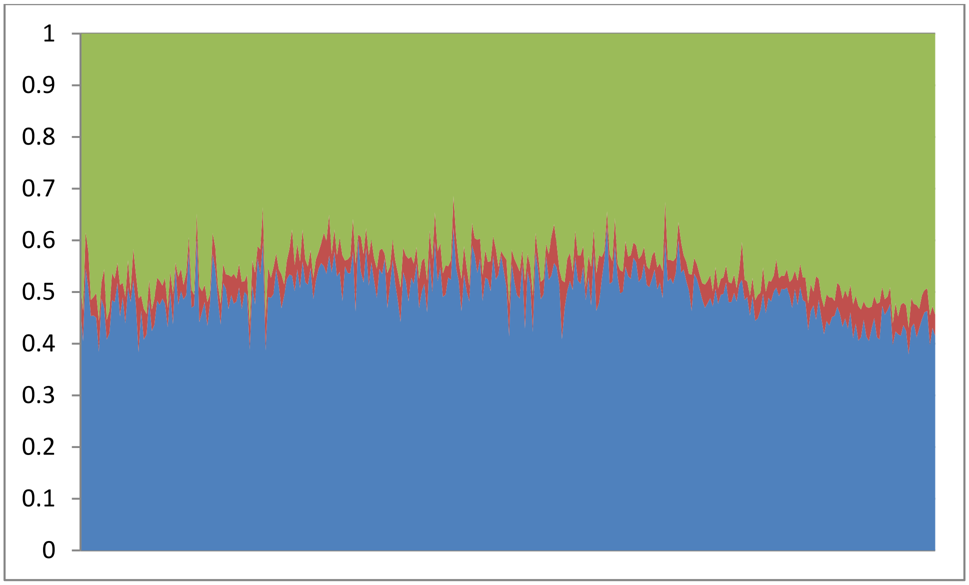

Figure S3 A graphical depiction of RIL Genotype proportions across the 14 chromosomes. Here blue = IM/IM, red = IM/PR (H), and green = PR/PR.
