# Supplementary material for: A High-Resolution Genetic Map of Yellow Monkeyflower Identifies Chemical Defense QTLs and Recombination Rate Variation
Source: G3 (Bethesda). 2014 Mar 13;4(5):813–21. doi: 10.1534/g3.113.010124 (PMC4025480; doi:10.1534/g3.113.010124)
Supplement: Supporting Information [file supp_g3.113.010124_TableS2.pdf]

Table S2 Results of GLM ANOVA. Factors with a significant effect on levels of a particular PPG are in bold type.

**PPG**	**Factor**	**F**	**df numerator, df denominator**	**p-value**
Conandroside	**RIL (Grow-up)**	6.69	207, 571	<0.001
	Damage?	3.51	1, 571	0.061
	**Grow-up**	10.23	2, 571	<0.001
	**Quant. method**	8.25	1, 571	0.004
PPG 5	**RIL (Grow-up)**	2.45	207, 571	<0.001
	Damage?	0.10	1, 571	0.756
	**Grow-up**	3.17	2, 571	0.044
	Quant. method	0.22	1, 571	0.641
PPG 7	**RIL (Grow-up)**	5.32	149, 193	<0.001
	Damage?	0.01	1, 193	0.944
	**Grow-up**	8.02	2, 193	<0.001
	Quant. method	---	---	---
